# Targeting peroxiredoxin 2 prevents hepatocarcinogenesis in metabolic liver disease models

**DOI:** 10.1172/JCI169395

**Published:** 2025-09-11

**Authors:** Emilie Crouchet, Eugénie Schaeffer, Marine A. Oudot, Julien Moehlin, Cloé Gadenne, Frank Jühling, Hussein El Saghire, Naoto Fujiwara, Shijia Zhu, Fahmida Akter Rasha, Sarah C. Durand, Anouk Charlot, Clara Ponsolles, Romain Martin, Nicolas Brignon, Fabio Del Zompo, Laura Meiss-Heydmann, Marie Parnot, Nourdine Hamdane, Danijela Heide, Jenny Hetzer, Mathias Heikenwälder, Emanuele Felli, Patrick Pessaux, Nathalie Pochet, Joffrey Zoll, Brian Cunniff, Yujin Hoshida, Laurent Mailly, Thomas F. Baumert, Catherine Schuster

**Affiliations:** 1 University of Strasbourg, INSERM, Institute for Translational Medicine and Liver Disease (ITM), UMR_S1110, Strasbourg, France.; 2 Liver Tumor Translational Research Program, Department of Internal Medicine, Division of Digestive and Liver Diseases, Simmons Comprehensive Cancer Center, University of Texas Southwestern Medical Center, Dallas, Texas, USA.; 3 Department of Gastroenterology and Hepatology, Mie University, Mie, Japan.; 4 UR 3072 Mitochondrion, Oxidative Stress and Muscle Plasticity, Biomedicine Research Center of Strasbourg (CRBS), University of Strasbourg, Strasbourg, France.; 5 Division of Chronic Inflammation and Cancer, German Cancer Research Center, Heidelberg, Germany.; 6 Cluster of Excellence iFIT (EXC 2180) “Image-Guided and Functionally Instructed Tumor Therapies,” Eberhard-Karls University of Tübingen, Tübingen, Germany.; 7 Hospital Group Saint Vincent, Strasbourg, France.; 8 Liver Transplant and Surgery Department, Trousseau Hospital, Tours, France.; 9 Department of Visceral and Digestive Surgery, Unit of Hepato-Bilio Pancreatic Surgery, Nouvel Hospital Civil, University Hospital of Strasbourg, Strasbourg, France.; 10 Broad Institute of Harvard and Massachusetts Institute of Technology, Cambridge, Massachusetts, USA.; 11 Department of Neurology, Harvard Medical School, Boston, Massachusetts, USA.; 12 Service de Physiologie et explorations fonctionnelles, University Hospital of Strasbourg, Strasbourg, France.; 13 Faculty of Medicine, University of Strasbourg, Strasbourg, France.; 14 Department of Pathology and Laboratory Medicine, University of Vermont Cancer Center, Larner College of Medicine, Burlington, Vermont USA.; 15 Gastroenterology and Hepatology Service, Strasbourg University Hospitals, Strasbourg, France.; 16 Institut Universitaire de France (IUF), Paris, France.

**Keywords:** Hepatology, Oncology, Carbohydrate metabolism, Preventative medicine, Therapeutics

## Abstract

Treatment options for advanced liver disease and hepatocellular carcinoma (HCC) are limited, and strategies to prevent HCC development are lacking. Aiming to discover therapeutic targets, we combined genome-wide transcriptomic analysis of liver tissues from patients with advanced liver disease and HCC and a cell-based system predicting liver disease progression and HCC risk. Computational analysis predicted peroxiredoxin 2 (*PRDX2*) as a candidate gene mediating hepatocarcinogenesis and HCC risk. Analysis of tissues from patients with HCC confirmed a perturbed expression of PRDX2 in cancer. In vivo perturbation studies in mouse models for hepatocarcinogenesis driven by metabolic dysfunction–associated steatohepatitis showed that specific *Prdx2* KO in hepatocytes improved metabolic liver functions, restored AMPK activity, and prevented HCC development by suppressing oncogenic signaling. Perturbation studies in HCC cell lines, a cell line–derived xenograft mouse model, and patient-derived HCC spheroids revealed that PRDX2 also mediates cancer initiation, cancer cell proliferation, and survival through its antioxidant activity. Targeting PRDX2 may therefore be a strategy to prevent HCC development in metabolic liver disease.

## Abstract 2

Peroxiredoxin 2 was identified as a candidate target for the prevention of liver cancer in patients with chronic liver disease induced by metabolic disorders.

## Graphical abstract

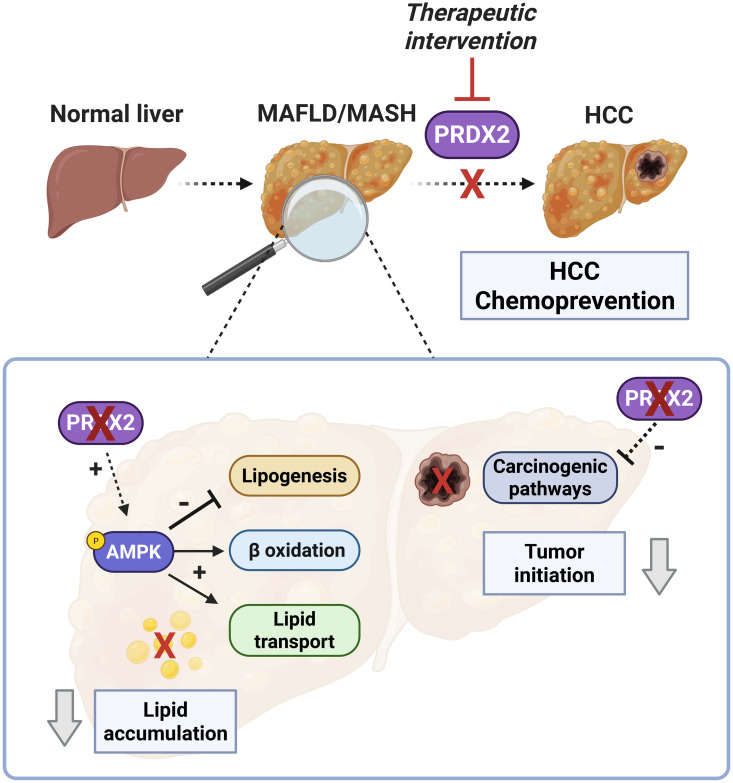

## Introduction

Chronic liver disease and hepatocellular carcinoma (HCC) are major public health burdens responsible for more than 2 million deaths per year worldwide (1). HCC accounts for the majority of liver cancer and is a leading and fast rising cause of cancer-related death globally (2). The major causes of HCC are chronic hepatitis B and C, alcohol abuse, and metabolic dysfunction–associated steatohepatitis (MASH). Although viral hepatitis has been a major cause of liver disease and HCC in the past, metabolic liver disease such as MASH will be the major cause of HCC in the future due to changes in lifestyle leading to increasing obesity and type II diabetes (3).

Despite tremendous efforts, current treatment options for HCC are still unsatisfactory. Only 30%–40% of patients with HCCs are eligible for curative surgical approaches and about 70% of them experience tumor recurrence within 5 years (2). Recently approved combinations of VEGF-targeting agents with immune checkpoint inhibitors targeting programmed cell death 1 (PD-1) have changed standard of care, but overall response rates and improvement of survival remain low (4). Prevention of HCC development and recurrence and treatment of the underlying etiology in patients at risk have therefore emerged as important strategies to decrease the overall HCC burden (5). However, discovery of chemopreventive approaches has been challenging due to the complexity of the disease biology and the absence of tangible models reflecting disease progression and HCC risk.

A prognostic liver signature (PLS) comprising 186 genes (73 poor-prognosis genes and 113 good-prognosis genes) has been shown to predict clinical progression of liver disease to HCC across all the main etiologies (6–9). A reduced version of this signature comprising 32 genes has been computationally determined and validated in multiple pan-etiologic patient cohorts (8, 9) and implemented in an FDA-approved diagnostic platform for clinical use (10). Recently, we developed a simple and robust human cell culture system recapitulating the clinical PLS in an inducible and reversible manner. This system, termed cPLS for cell culture PLS, has been shown to model the cell circuits relevant for liver disease progression and HCC risk and has allowed the discovery of potentially novel targets and compounds for liver disease treatment and HCC prevention (11–14).

To identify clinically relevant targets for HCC prevention, we combined transcriptomic analysis of liver tissues from patients with fibrosis/cirrhosis and the cPLS system. By performing computational analyses, followed by validation in mouse models, cell-based systems, and patient-derived models, we identified peroxiredoxin 2 (PRDX2) as a candidate target for HCC prevention in MASH.

## Results

### PRDX2 is associated with liver disease progression and HCC risk.

**Figure 1:**
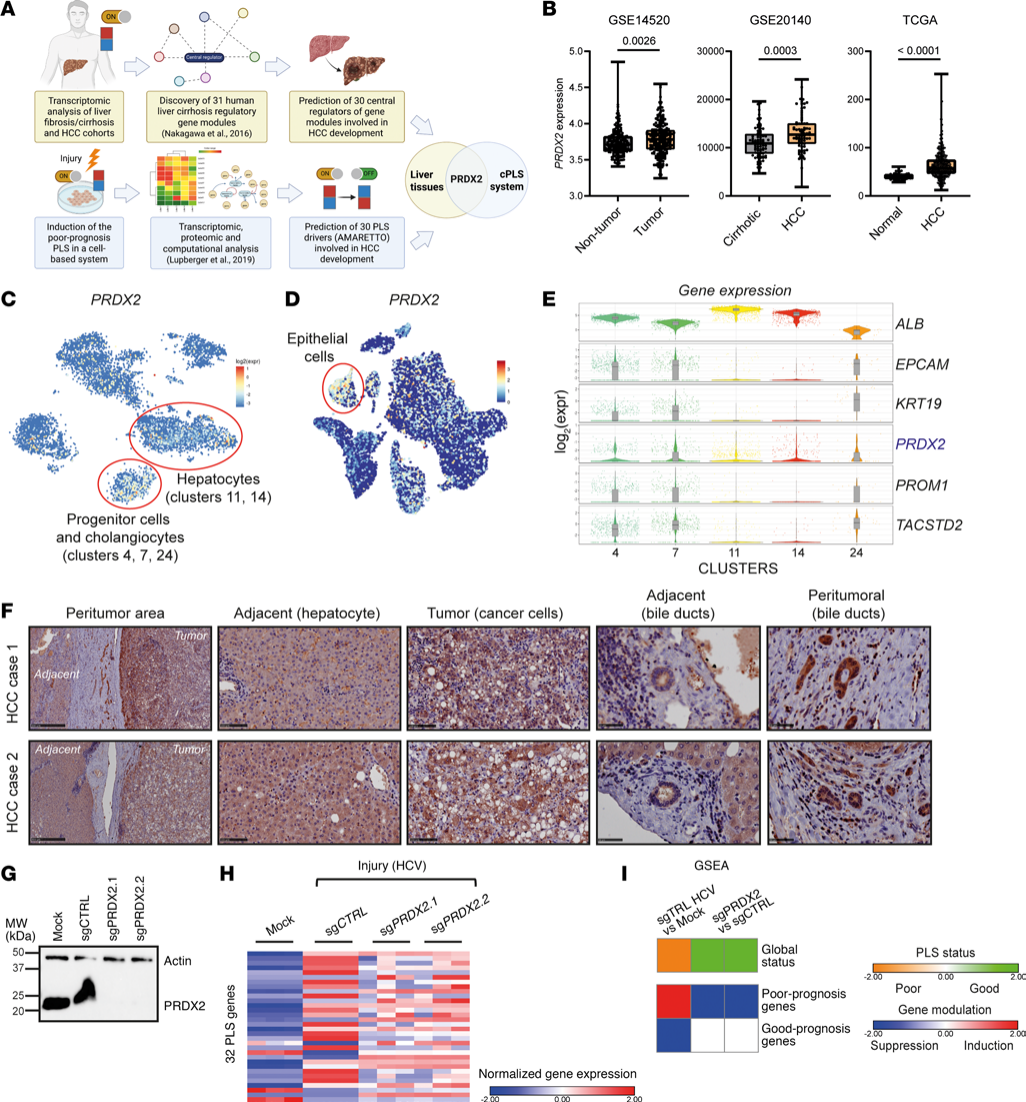
PRDX2 expression is associated with HCC in patients. (**A**) Identification of HCC chemoprevention candidate targets using genome-wide transcriptomic analyses of patient liver tissues and the cPLS system. (**B**) Analysis of *PRDX2* expression in liver tissues of clinical cohorts at transcriptomic level (GSE14520: nontumor *n* = 220; tumor *n* = 225. GSE20140, cirrhotic *n* = 82; HCC *n* = 80. The Cancer Genome Atlas, TCGA: normal *n* = 49; HCC *n* = 358). Exact *P* values are indicated on the graphs; Mann-Whitney *U* test. (**C** and **D**) Expression *t-*SNE map of *PRDX2* from single-cell transcriptomes of patient liver tissues (**C**, 9 nondiseased human livers; **D**, 5 nondiseased and 5 cirrhotic human livers). Cells sharing similar transcriptome profiles are grouped by clusters, and each dot represents 1 cell. The color bar indicates log_2_ normalized expression. Red circles indicate the epithelial cell compartments. Data extracted from **C** (29) and **D** (30). (**E**) Violin plots showing *PRDX2* expression in the epithelial cell compartments (clusters 4, 7, 11, 14, and 27). *ALB* = mature hepatocytes; *EPCAM*, *KRT19*, *PROM1,* and *TACSTD2* = markers of progenitor cells. (**F**) PRDX2 protein expression in HCC tumor tissues. Representative IHC images showing PRDX2 expression in hepatocytes and cholangiocytes in adjacent nontumoral tissue and HCC tumoral tissues (see Supplemental Figure 2 and Supplemental Table 1). Scale bars: 500 μm for peritumor area, 100 μm for adjacent and tumor and 50 μm for bile ducts. (**G**) Generation of Huh7.5.1 cells KO for *PRDX2*. *PRDX2* KO was assessed by Western blot analysis. (**H** and **I**) KO of *PRDX2* in the cPLS system reverses the poor-prognosis PLS induced by persistent HCV infection. (**H**) Detailed PLS gene expression profiles (32-gene signature) are shown. Heatmaps show the mean expression of the genes normalized to housekeeping genes (*z* scores of log_2_ normalized data). (**I**) Simplified heatmaps showing the PLS global status (top) and the global variation of the PLS poor- and good-prognosis genes (bottom), calculated using gene set enrichment analysis (GSEA).

To identify HCC chemoprevention targets, we applied 2 complementary strategies (Figure 1A). First, we used transcriptomic analyses of liver tissues of patient cohorts with advanced liver disease progressing to HCC. Based on regulatory gene network modeling performed in a previous study by using genome-wide transcriptome profiles of liver tissue from 523 patients with fibrosis/cirrhosis (9), gene coexpression meta-analysis followed by planar filtered network analysis (PFNA) (15) identified 31 coregulated gene modules associated with liver disease progression and HCC development (NCBI’s Gene Expression Omnibus, GEO GSE64520) (9). Key regulatory genes in each gene module were determined by key driver analysis, which prioritizes driver genes by measuring the impact on the downstream genes (9, 16) (Table 1).

Second, we applied our cPLS model system as a functional liver disease cell circuit assay to identify candidate targets involved in liver disease progression to cancer (11) (Figure 1A). DMSO-differentiated Huh7.5.1 (Huh7.5.1^dif^) cells were subjected to persistent injury to model the clinical poor-prognosis PLS. Then, we performed transcriptomic analyses (GEO GSE126831) and used the AMARETTO algorithm to infer the regulatory networks underlying the poor-prognosis PLS development (14, 17). The algorithm subsequently identified a list of candidate regulators by connecting known regulatory driver genes with coexpressed target genes (see Methods). The list was narrowed down to 30 candidates according to (a) high expression in liver tissues, (b) association with liver disease in patients, and (c) ability of the encoded protein to be targeted by drugs (Table 2).

We identified known pathways involved in liver disease progression toward HCC development, such as the TNF-α/NF-κB pathway (*RELA*, *FOS*, *CREB5*, *TNFA*) (11, 18, 19), the proto-oncogene *MYC* (20), histone modifiers (*HDAC9*) (13), the IL-6/STAT3 pathways (14), and the *PPAR* gene family (*ACSL3*, *NR1H3*, *PPAR*A) (21), confirming the validity of our approach (Tables 1 and 2). Among the candidates, we focused our interest on *PRDX2*. *PRDX2* encodes for a soluble cellular and secreted enzyme, which detoxifies H_2_O_2_ generated during oxidative stress, that could be targeted by small molecules. PRDX2 was reported to promote tumor growth in different solid cancers, including colorectal, non–small cell lung, ovarian, and breast cancers (22). However, its role in liver disease progression and HCC development is still controversial (23–27). Considering the importance of oxidative stress in liver disease progression (28), we explored the role of PRDX2 in hepatocarcinogenesis.

We first investigated *PRDX2* expression in patient cohorts. Analysis of *PRDX2* expression using publicly available data (GEO and The Cancer Genome Atlas) revealed that *PRDX2* expression was increased in tumor liver tissues compared with adjacent nontumoral tissues (Figure 1B). At the single-cell level, *PRDX2* was expressed in different cell compartments, with the highest expression level in epithelial cells, including hepatocytes and cholangiocytes in normal and fibrotic liver tissues (29, 30) (Figure 1, C–E, and Supplemental Figure 1 for detailed analysis). Figure 1E shows that *PRDX2* expression was higher in cell clusters expressing high levels of *EPCAM*, *PROM1*, and *TACSTD2*/*TROP2*, 3 markers of stem cells (29), indicating that *PRDX2* is expressed in progenitor cells and may be associated with stemness (Figure 1E).

To validate these findings at the protein level, we analyzed PRDX2 expression in HCCs and paired adjacent nontumoral tissues from our Strasbourg HCC cohorts (Figure 1F, Supplemental Figure 2, and Supplemental Table 1). We observed that PRDX2 expression was increased in tumor tissues of 6 out of 9 patients, with the highest expression in HCC cells and peritumoral cholangiocytes (Figure 1F and Supplemental Figure 2A). PRDX2 protein levels were higher in the peritumoral area where the cancer cells were exposed to abnormal extracellular matrix or immune cell infiltration, with a gradient from the periphery to the core of the tumor (Figure 1F and Supplemental Figure 2B). This heterogeneity may explain the difference observed in previous studies (23–27). Of note, PRDX2 protein expression was also increased in cancer cells compared with isolated primary human hepatocytes (PHHs) (Supplemental Figure 3).

A previous study demonstrated that the molecular pathways/functions that have been involved in the pathogenesis of liver disease and carcinogenesis can be grouped in 31 different gene modules (9). *PRDX2* was identified as a key regulatory gene in central module 8, coregulated with the tumor suppressor *HINT1* (Supplemental Figure 4), which is the only module associated with increased HCC risk independently from cirrhosis (9). Altogether, these results support a role of PRDX2 in carcinogenesis.

To further validate the role of PRDX2 in carcinogenesis, we performed a loss-of-function study in the cPLS model. Huh7.5.1^dif^ cells were injured using persistent HCV infection, a well-described inducer of the poor-prognosis PLS associated with cancer risk (11). *PRDX2* was then knocked down, and we evaluated the status of the cPLS (Figure 1, G and H, and Supplemental Tables 2). The global induction or suppression of the cPLS genes was determined by gene set enrichment analysis (GSEA) using noninfected cells as reference and by comparing *PRDX2-*KO cells to control cells (11) (Figure 1I and Supplemental Table 2). *PRDX2* KO reversed the poor-prognosis cPLS associated with HCC risk, indicating a functional role of PRDX2 in the cell circuits driving liver disease progression to cancer.

### Prdx2 is involved in metabolic liver disease progressing to cancer in vivo.

**Figure 2:**
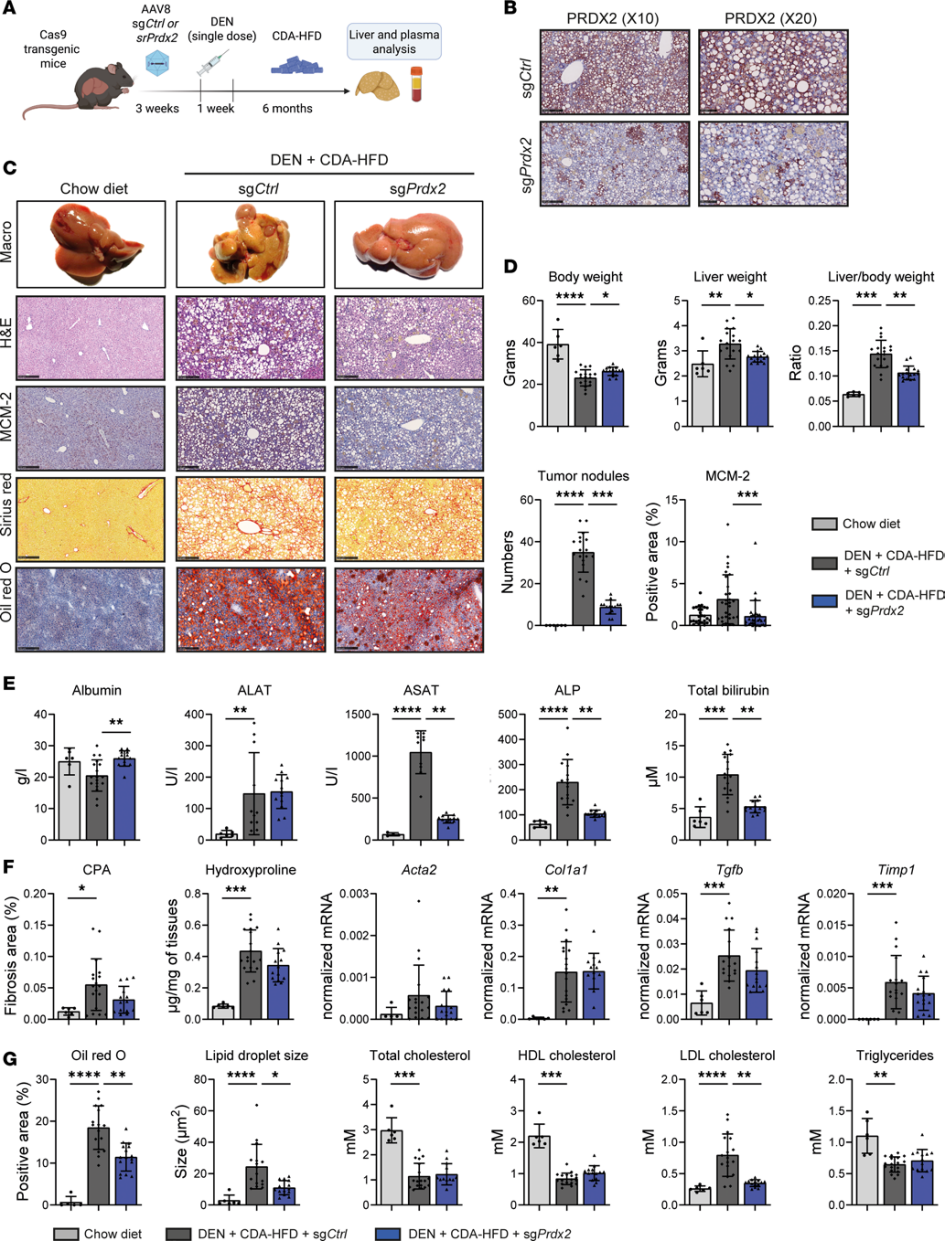
*Prdx2* KO improves liver steatosis and prevents HCC development in a MASH/HCC mouse model. (**A**) AlbCre-Cas9 mice were injected with AAV8 vectors coding for a control or a *Prdx2*-specific sgRNA. Liver disease was induced by single-dose DEN injection and CDA-HFD (chow diet *n* = 6, sg*Ctrl* *n* = 18, sg*Prdx2* *n* = 16). (**B**) IHC analysis of PRDX2 expression in mouse liver tissues validating *Prdx2* KO in hepatocytes. Positive islets in KO animals correspond to nontransduced areas. Scale bars: 250 μm (×10) and 100 μm (×20). (**C** and **D**) *Prdx2* KO prevents HCC development in vivo. (**C**) Representative morphometric analysis, H&E coloration, and IHC analyses of mouse livers. Scale bar: 250 μm. (**D**) Body weight, liver weight and liver-to-body weight ratios, the number of surface tumor nodules, and quantification of the MCM-2 cell proliferation marker are reported. (**E**) *Prdx2* KO improves liver function. Analysis of liver function by measurement of albumin, aspartate, and alanine aminotransferases (ASAT, ALAT), alkaline phosphatase (ALP), and total bilirubin. (**F**) *Prdx2* KO does not modulate liver fibrosis. Fibrosis levels were evaluated through quantification of collagen proportionate area (CPA) of Sirius red staining performed in **C**, hydroxyproline quantification, and by fibrotic gene expression (qRT-PCRs). (**G**) *Prdx2* KO improves liver steatosis. Lipid accumulation and lipid droplet size were evaluated through quantification of Oil Red O staining performed in **C**. Serum lipid profile analysis of cholesterol (total, HDL, and LDL) and triglycerides are shown. The graphs show mean ± SD, *P < 0.05, **P < 0.01, ***P < 0.001, ****P < 0.0001 (Kruskal-Wallis test followed by Dunn’s multiple-comparison test).

To further investigate the functional role of PRDX2 in carcinogenesis in vivo, we performed a hepatocyte-specific *Prdx2* KO in a Cas9 transgenic mouse model for hepatocarcinogenesis induced by a single dose of diethylnitrosamine (DEN) and a choline-deficient L-amino acid–defined high-fat diet (CDA-HFD) (Figure 2A) (31). To generate the KO, we applied a sgRNA validated for functional KO in a mouse cell line (Supplemental Figure 5). *Prdx2* was specifically targeted in hepatocytes by combining a Cre-dependent expression of the Cas9 endonuclease in the liver and the hepatic delivery of sgRNA to hepatocytes using an adeno-associated virus 8 (AAV8) vector (32). *Prdx2* KO was confirmed by suppression of protein expression in hepatocytes (Figure 2B). PRDX2-positive islets were most likely nontransduced cells (Figure 2B).

*Prdx2* KO improved liver weight and decreased tumor burden, tumor size, and cell proliferation, as shown at macroscopic observations and by the decreased expression of the proliferation marker minichromosome maintenance complex component 2 (MCM2) (Figure 2, C and D). Importantly, *Prdx2* KO improved liver function as shown by serum albumin, liver enzymes, and total bilirubin levels, confirming the absence of toxicity in mouse liver (Figure 2E). The safety of *PRDX2* targeting in human hepatocytes was also confirmed by the absence of toxicity in PHHs (Supplemental Figure 6). We then assessed the effect of *Prdx2* KO on liver fibrosis and steatosis. Collagen proportionate area (CPA), hydroxyproline quantification, and assessment of fibrogenic gene expression (*Acta2*, *Col1a*, *Tgfb1*, and *Timp1*) showed a minor effect of *Prdx2* KO on liver fibrosis (Figure 2, C and F). In contrast, we observed a decrease in liver steatosis (Figure 2, C and G) and LDL cholesterol levels in *Prdx2*-KO animals, supporting a functional role for *Prdx2* in lipid metabolism regulation. Finally, we assessed the expression of the other *Prdx*s family members and of thioredoxin (*Txn*) in mouse livers. TXN is involved in PRDXs protein recycling and was already described as an HCC marker (33, 34). *Prdx2* KO had no significant impact on other *Prdx*s’ expression, indicating that the effect on liver disease is not due to compensatory effect (Supplemental Figure 7). Of note, only *Prdx2* expression showed a significant increase in MASH/HCC animals. A trend was observed for *Prdx6*, which is also described to play a role in MASH and HCC development (35, 36). *Txn* expression was increased in CDA-HFD mouse livers, and a downward trend was observed for *Txn* expression in *Prdx2*-KO animals, in line with disease improvement and reduction of carcinogenesis.

Altogether, these data demonstrate that targeting PRDX2 in diseased livers improves liver steatosis and prevents HCC development, most likely independently from fibrosis.

### Prdx2 KO prevents HCC development by improving lipid metabolism and regulating procarcinogenic signaling pathways.

**Figure 3:**
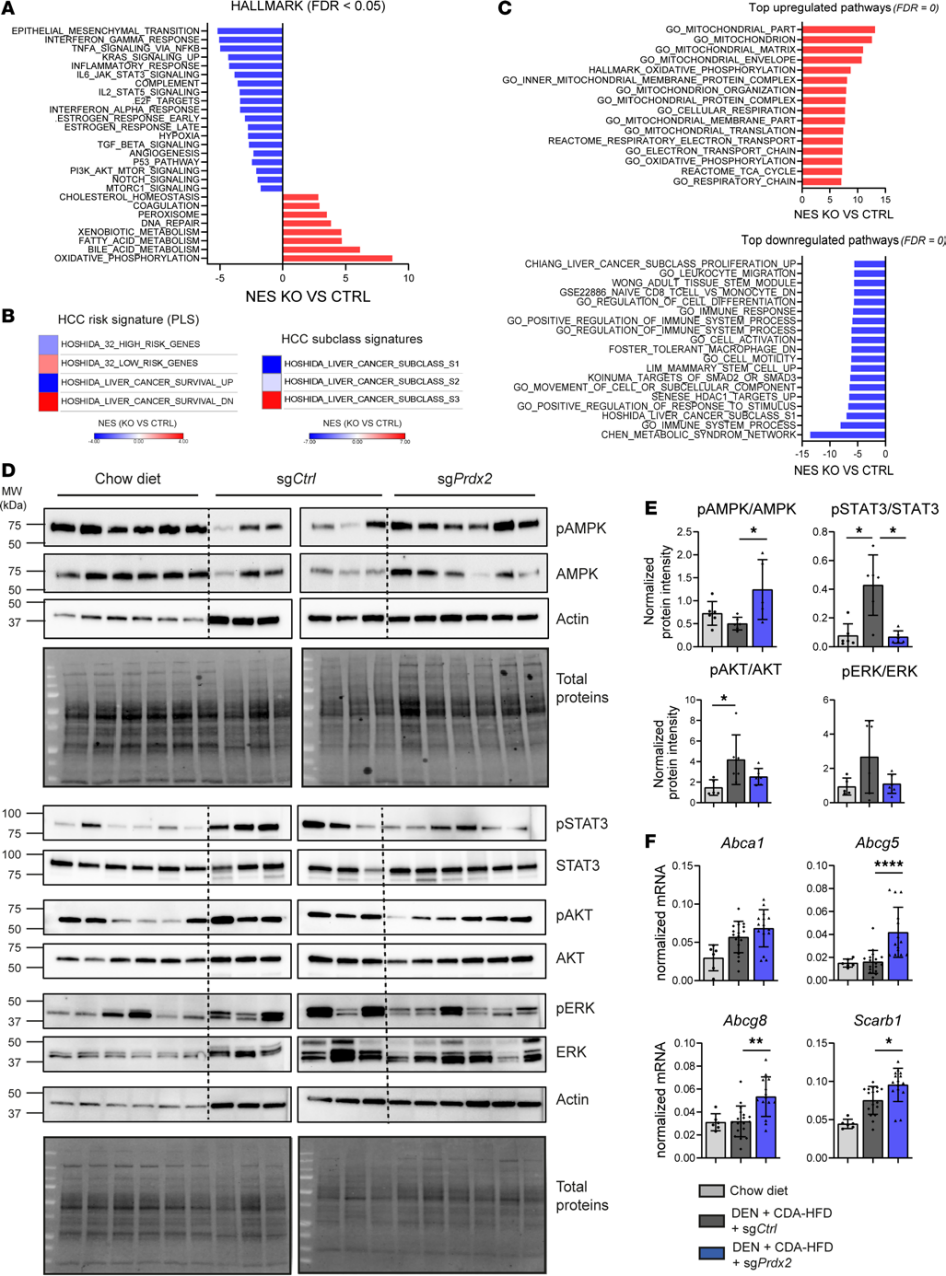
PRDX2 is involved in HCC development by improving lipid metabolism and transport and regulating procarcinogenic signaling pathways. (**A**) RNA-Seq analysis of mouse liver tissues. Graph shows enrichment of HALLMARK gene sets relevant for liver disease expressed as normalized enrichment score (NES) obtained from GSEA (*Prdx2* KO vs. control mice). *n* = 3 per group. FDR < 0.05. (**B**) *Prdx2* KO reverses poor-prognosis PLS and modulates HCC subclass signature in MASH/HCC mouse model. Left panel: heatmaps show modulation of 32-gene PLS (HOSHIDA_32) and 186-gene PLS (HOSHIDA_LIVER_CANCER) (7). Right panel: Heatmap shows modulation of the liver cancer subclass gene sets (37). (**C**) Top upregulated and downregulated pathways enriched in *Prdx2*-KO mice. Graphs show unbiased selection of top enriched gene sets relevant for liver disease. FDR = 0. (**D**) *Prdx2* KO restores AMPK activity in mouse livers. Western blot analysis of pAMPK subunit α (T172) and total AMPKα. Graph shows protein quantification as mean ± SD of normalized protein intensity (normalization to total proteins), *P < 0.05 (Kruskal-Wallis test, followed by Dunn’s multiple-comparison test). *n* = 6 per group. (**E**) *Prdx2* KO improves lipid transport. Expression of genes involved in lipid and bile acid transport measured by qRT-PCR. Graphs show mean ± SD of normalized mRNA (to *Gapdh*), *P < 0.05, **P < 0.01, ***P < 0.001, ****P < 0.0001 (Kruskal-Wallis test followed by Dunn’s multiple-comparison test). *Scarb1*, scavenger receptor class B type 1*; Abca1,* *Abcg5*, *Abcg8*, ATP binding cassette subfamily A member 1 and subfamily G members 5 and 8. (**F**) *Prdx2* KO suppresses procarcinogenic pathways in mouse livers. Western blot analysis of p-STAT3 (Y705) and total STAT3, p-Akt (S473) and total Akt, p-Erk1 (T202/Y204)/Erk2 (T185/Y187), and total Erk1/2 in mouse livers. Graph shows protein quantification as mean ± SD of normalized protein intensity (normalization to total proteins), *P < 0.05 (Kruskal-Wallis test, followed by Dunn’s multiple-comparison test). *n* = 6 per group.

To decipher the mechanism of action, we performed RNA-Seq analysis of mouse liver tissues. GSEA analysis (HALLMARK) showed that *Prdx2* KO suppresses epithelial-mesenchymal transition (EMT), pathways related to liver inflammation (e.g., TNF-α signaling, inflammatory response) and to carcinogenesis such as KRAS, cell cycle, MAPK/ERK, IL-6/STAT3, and to a lesser extent PI3K/AKT signaling (Figure 3A and Supplemental Table 3) (5). We also observed a general improvement of hepatic metabolism, in particular on bile acid and fatty acid metabolism (Figure 3A), in line with the decrease of liver steatosis observed in *Prdx2*-KO mice (Figure 2). In accordance with our cell-based cPLS data (Figure 1), *Prdx2* KO reversed the PLS poor-prognosis status to a good-prognosis status, supporting the hypothesis that targeting *Prdx2* in hepatocytes reduces the HCC risk (Figure 3B). HCCs are usually classified into different subclasses, including proliferative and nonproliferative HCCs (37, 38). Interestingly, *Prdx2* KO induced a shift from HCC proliferative signatures to nonproliferative HCC signatures associated with a well-differentiated and less-aggressive phenotype (Figure 3B). In line with this finding, we observed a decrease in the stemness marker CD44 in *Prdx2*-KO mouse livers (Supplemental Figure 8).

Unbiased selection of the top upregulated pathways showed an increase in mitochondria response, oxidative phosphorylation, and TCA cycle, indicating that the decrease in lipid accumulation is mediated by the increased mitochondrial function in *Prdx2*-KO mice (Figure 3C) (39). In contrast, the most important downregulated pathways related to liver disease were associated with metabolic syndrome (40), macrophages, immune responses, and inflammation (Figure 3C). These observations were confirmed by a decrease in some key cytokines and secreted factors mediating macrophage recruitment and inflammation (e.g., CD14, CCL2, CCL5), and metabolic syndrome (IGF-BP1) (Supplemental Figure 9).

AMPK, the primary sensor of cellular energy, is a gatekeeper of hepatic metabolism, bridging inflammation, oxidative stress, mitochondrial functions, and lipid metabolism (41, 42). Because AMPK function is often impaired in metabolic liver disease and liver cancer (43–45), we hypothesized that AMPK activity is impaired in DEN/CDA-HFD and that *Prdx2* KO may restore AMPK functions. Immunoblot analysis of mouse livers confirmed that activation of AMPKα (the catalytic subunit) was decreased in DEN/CDA-HFD control animals and restored in *Prdx2-*KO mice (Figure 3, D and E). Activation of AMPK in mouse livers translated into an increase in degradation of fatty acids associated with an increase in lipid export and transport, as reflected by expression of key genes involved in lipid transport, cholesterol efflux (scavenger receptor class B member 1, *Scarb1*, and the ATP binding cassette, *Abca1*), and biliary excretion of cholesterol (*Abcg8*, *Abcg5*) (46) (Figure 3F).

Regarding the carcinogenic pathways, we confirmed by Western blot analysis that the STAT3, MAPK/ERK, and AKT pathways were suppressed in *Prdx2*-KO mouse livers, with the strongest effect on STAT3 signaling (Figure 3, D and E). Of note, expression of the tumor suppressor p53 and related pathways were decreased in *Prdx2*-KO mice, which reflects an improvement of liver functions (Supplemental Figure 10).

In addition to peroxide detoxification, PRDX2 is known to have different functions such as chaperone, binding partner, and enzyme activator (47). PRDX2 functions are dictated by its conformation and redox state; PRDX2 dimers act preferentially as antioxidant enzymes, whereas decamers or high molecular weight forms (HMWs) act as chaperone (47). We therefore analyzed PRDX2 conformation in mouse and patient livers (Supplemental Figures 11 and 12). Although HMW forms were detected, the vast majority of PRDX2 protein was present as dimers, suggesting that PRDX2 antioxidant activity and regulation of redox state functions may be predominant in the liver (Supplemental Figures 11 and 12) (47).

**Figure 4:**
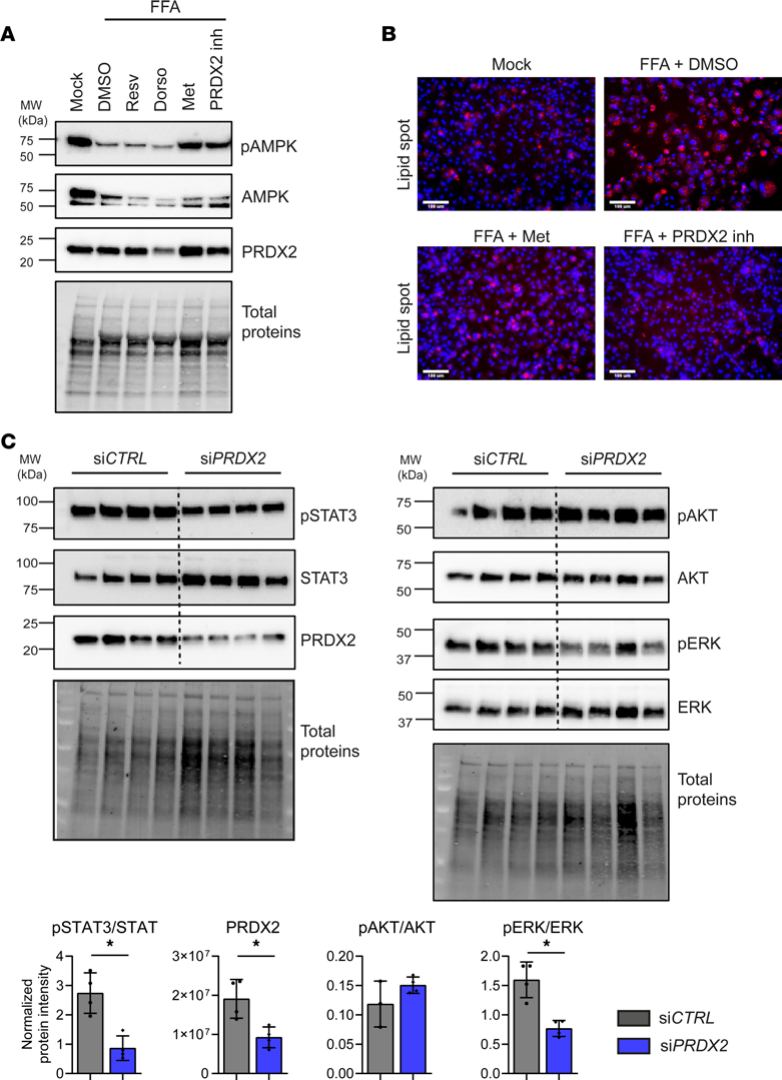
Targeting PRDX2 improves lipid metabolism and suppresses procarcinogenic signaling pathways in primary human hepatocytes. (**A** and **B**) PRDX2 inhibitor restores AMPK activity and decreases lipid accumulation in free fatty acid–treated primary human hepatocytes (PHHs). (**A**) Western blot analysis of p-AMPKα (T172), total AMPKα, and PRDX2 in treated PHHs. (**B**) Representative images of lipid accumulation in treated PHHs. Neutral lipids are shown in red. Nuclei were counterstained in blue (DAPI). Scale bar: 100 μm. One representative experiment out of 2 is shown. (**C**) *Prdx2* KD suppress procarcinogenic pathways in PHHs. Western blot analysis of p-STAT3 (Y705) and total STAT3, p-Akt (S473) and total Akt, p-Erk1 (T202/Y204)/Erk2 (T185/Y187), and total Erk1/2 and PRDX2 in PHHs. The graph shows protein quantification as mean ± SD of normalized protein intensity (normalization to total proteins), *P < 0.05 (Mann-Whitney *U* test). One representative experiment out of 2 performed in quadruplicate is shown.

To validate the clinical translatability of our findings, we performed perturbation studies in PHHs isolated from patient livers. As observed in animals, treatment of PHHs with free fatty acids impaired AMPK activation, which was restored by an inhibitor of PRDX2 enzymatic activity. AMPK activation resulted in decreased lipid accumulation, at the same extent as metformin, a well-described AMPK activator (Figure 4, A and B). Moreover, *PRDX2* KD or inhibition significantly suppressed STAT3 and ERK pathways (Figure 4C and Supplemental Figure 13). AKT activation was unchanged in *PRDX2*-KD PHHs (Figure 4C), indicating that the decrease in AKT pathway observed in mice is most likely due to a general improvement of liver functions.

Altogether, these results indicate that targeting PRDX2 in hepatocytes prevents HCC development in MASH by (a) improving metabolic liver disease and (b) suppressing procarcinogenic signaling pathways.

### PRDX2 KD using GalNac siRNAs prevents hepatocarcinogenesis in a preclinical mouse model of MASH/HCC.

**Figure 5:**
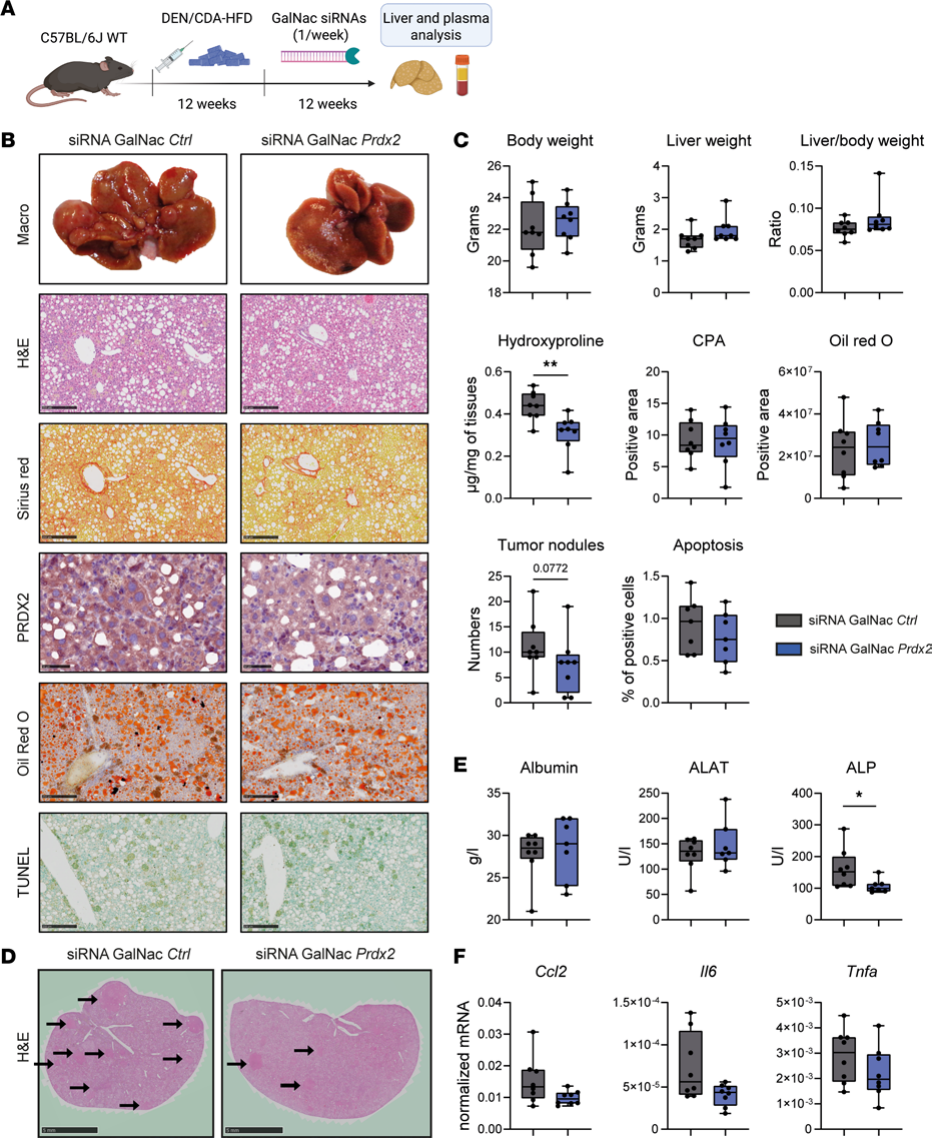
*Prdx2* KD in hepatocytes prevents HCC development in a preclinical mouse model for MASH/HCC. (**A**) C57BL/6 WT mice were injected with DEN (single dose) and fed with CDA-HFD for 12 weeks before injection of GalNac siRNAs targeting *Prdx2* expression or nontargeting control (weekly injection for 12 weeks). *n* = 8 for each group. (**B** and **C**) *Prdx2* KO prevents HCC development in vivo. (**B**) Representative morphometric analysis, H&E, Sirius red, and Oil Red O coloration and TUNEL assay of mouse livers are shown. Scale bar: 250 μm. *Prdx2* KD was assessed by IHC analysis. Scale bar: 50 μm. As the mice were euthanized 1 week after the last siRNA GalNac injection, the level of expression of PRDX2 re-increased in the livers. (**C**) Body weight, liver weight, and liver-to-body weight ratios and the number of surface tumor nodules are reported. Fibrosis levels were evaluated through quantification of collagen proportionate area (CPA) of Sirius red staining performed in **B** and hydroxyproline quantification. Lipid accumulation was evaluated through quantification of Oil Red O staining performed in **B**. Levels of apoptosis were evaluated by TUNEL assay (**B**) and quantification of positive cells. (**D**) *Prdx2* KO decreases the number of nodules in mouse livers. Representative images of H&E coloration are shown. Arrows indicate tumor nodules. Scale bars: 5 mm. (**E**) Analysis of liver function by measurement of albumin, alanine aminotransferases (ALAT), and alkaline phosphatase (ALP). (**F**) Effect of *Prdx2* KD on liver inflammation. Expression levels of C-C motif chemokine ligand 2 (*Ccl2*), IL-6 (*Il6*), and TNF-α (*Tnfa*) were assessed by qRT-PCR in mouse livers. In box-and-whisker plots, boxes represent the 75th and 25th percentiles, the whiskers represent the most extreme data points within IQR × 1.5, and the horizontal bar represents the median. The circles indicate observation for each sample. *P < 0.05, **P < 0.01 (Mann-Whitney *U* test).

To assess whether targeting PRDX2 in hepatocytes may be a therapeutic approach to prevent HCC in MASH, we performed a *Prdx2* KD within therapeutic windows after MASH development. To mimic a therapeutic approach, we designed a *Prdx2*-specific siRNA covalently linked to a ligand containing 3 N-acetylgalactosamine residues (GalNAc siRNAs). Efficacy of *Prdx2* GalNac siRNA was validated in a Hepa1.6 mouse cell line (Supplemental Figure 14). After 12 weeks of diet, mice were randomized into 2 groups receiving either GalNac si*Ctrl* or GalNac si*Prdx2* (Figure 5A). Animal livers were collected 1 week after the last injection. As observed for the *Prdx2-*KO model, *Prdx2* KD in hepatocytes had only a minor effect on fibrosis levels (Figure 5, B and C). Moreover, *Prdx2*-KD animals developed less and smaller tumors (Figure 5, B–D). *Prdx2* KD induced a reduction in ALP, indicating a decrease in liver and bile duct damage (Figure 5E). In contrast to the previous model, we observe neither a reduction in lipid accumulation nor an increase in AMPK activation, most likely because MASH was already robustly established after 12 weeks of diet (Figure 5, B and C, and Supplemental Figure 15A). Nevertheless, Oil Red O staining showed a reduced number of foam cells in mouse livers, suggesting improvement of lipid transport and reduction of inflammation (Supplemental Figure 15B). In line with this observation, we observed a decrease in *Ccl2*, *Il6*, and *Tnfa* expression in KD animals (Figure 5F). Although additional studies are needed, these results indicate that targeting PRDX2 in hepatocytes prevents HCC development.

### Prdx2 KO prevents tumor initiation and progression.

**Figure 6:**
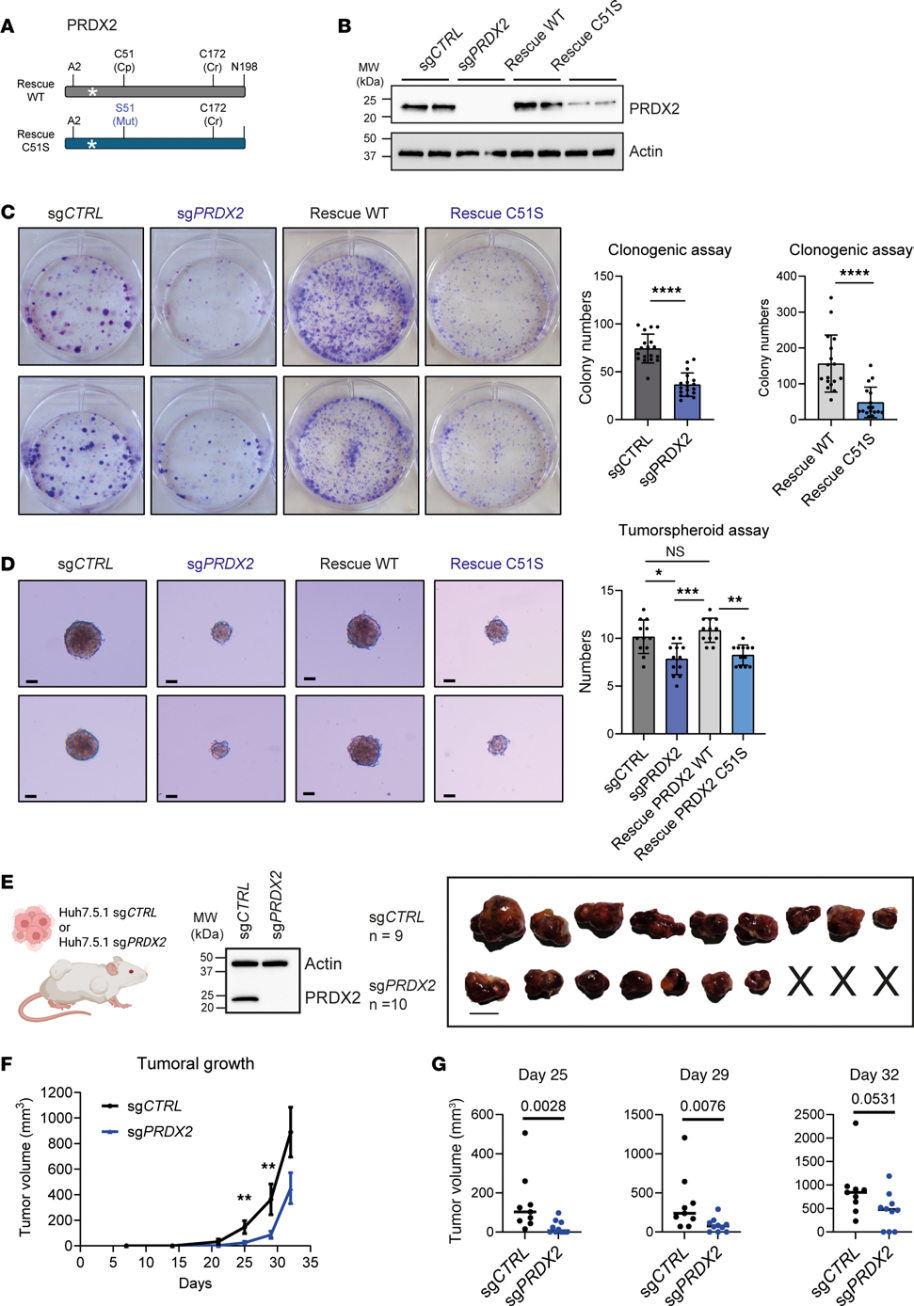
Targeting PRDX2 prevents tumor initiation and progression. (**A** and **B**) Generation of a PRDX2 inactive mutant. (**A**) WT PRDX2 (in gray) is a thiol peroxidase of 198 amino acids. The catalytic activity is mediated by the active peroxidatic cysteine (Cp) C51 and the resolving cysteine (Cr) C172. To generate an inactive protein, the active cysteine C51 was substituted by a serine (in blue). To allow reexpression of WT and mutant PRDX2 in sg*PRDX2* cells, the target site of the sgRNA was mutated in both forms (white star). The expression of WT and C51S PRDX2 (rescue) was assessed by Western blot analysis (**B**). (**C** and **D**) PRDX2 enzymatic activity is required for tumor initiation. Clonogenic and tumor spheroid assays were performed on sg*CTRL*, sg*PRDX2* cells, and KO cells with rescued expression of WT or C51S PRDX2 mutants. Representative images are shown from 3 independent experiments performed in 6 replicates (*n* = 18). Numbers of colonies or of tumor spheroids were assessed using Celigo Image Cytometer (Nexcelcom Biosciences). The graphs show mean ± SD of colony of tumor spheroid numbers. For **C**, ****P < 0.0001 (Mann-Whitney *U* test); for **D**, *P < 0.05, **P < 0.01, ***P < 0.001 (Kruskal-Wallis test followed by Dunn’s multiple-comparison test). (**E**–**G**) *PRDX2* KO slows down tumor growth in a CDX mouse model. (**E**) Huh-7.5.1 sg*CTRL* or sg*PRDX2* cells were subcutaneously injected in immunodeficient NRG mice (sg*CTRL* *n* = 9; sg*PRDX2* *n* = 10). In sg*PRDX2* group, 3 out of 10 tumors did not grow. Tumoral development was assessed by repeated measures of tumor size, and representative morphometric analysis of the tumors is shown. Scale bar: 1 cm. (**F**) The graph shows mean ± SD of tumor volume in relation to time. (**G**) To appreciate the difference between the 2 groups, individual graphs for days 25, 29, and 32 are shown. Exact *P* values are indicated (Mann-Whitney *U* test).

RNA-Seq data showed that *Prdx2* KO suppressed procarcinogenic pathways and cell cycle–related pathways (Figure 3A). We hypothesize that targeting PRDX2 may have an impact on tumor initiation through its antioxidant function. First, to link the antioxidant function of PRDX2 to HCC initiation, we generated a mutation in the PRDX2 catalytic site by substituting the peroxidatic cysteine residue (Cp) by a serine (C51S), resulting in peroxidase inactive mutant expression (Figure 6A) (48). Then, we rescued PRDX2 WT or C51S mutant expression in *PRDX2*-KO cancer cells (Figure 6, A and B) and assessed cancer cell phenotype by performing clonogenic and tumor spheroid assays (Figure 6, C and D). We observed a reduced number of colonies and tumor spheres in KO cells (Figure 6, C and D), indicating that *PRDX2* KO impaired tumor initiation and self-renewal properties of cancer cells. Moreover, while the rescue with WT PRDX2 restored tumor spheroid and colony formation, the peroxidase-inactive C51S mutant showed the same profile as *PRDX2*-KO cells (Figure 6, C and D), indicating that the antioxidant function of PRDX2 is involved in tumor initiation.

**Figure 7:**
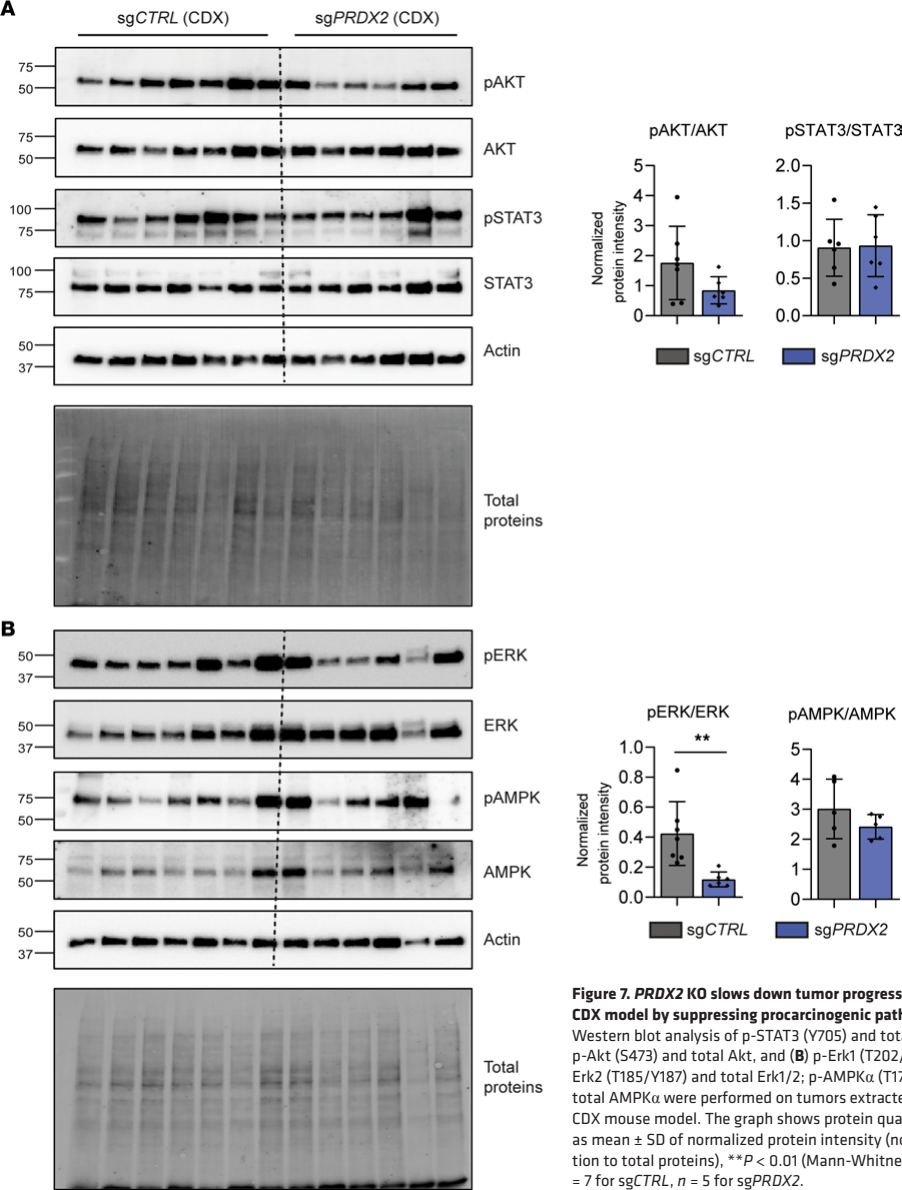
*PRDX2* KO slows down tumor progression in a CDX model by suppressing procarcinogenic pathways. (**A**) Western blot analysis of p-STAT3 (Y705) and total STAT3, p-Akt (S473) and total Akt, and (**B**) p-Erk1 (T202/Y204)/Erk2 (T185/Y187) and total Erk1/2; p-AMPKα (T172) and total AMPKα were performed on tumors extracted from the CDX mouse model. The graph shows protein quantification as mean ± SD of normalized protein intensity (normalization to total proteins), **P < 0.01 (Mann-Whitney *U* test), *n* = 7 for sg*CTRL*, *n* = 5 for sg*PRDX2*.

Second, we assessed the effect of *PRDX2* KO on tumor growth in a cell line–derived xenograft (CDX) mouse model. *PRDX2*-KO or control cells were subcutaneously injected into nonobese diabetic NRG mice (Figure 6E). *PRDX2* KO reduced tumor growth, with 3 out of 10 *PRDX2*-KO mice not showing any tumor formation (Figure 6, E–G). Analysis of the signaling pathways in the tumors showed a decrease in AKT and ERK signaling, but not of STAT3 and AMPK signaling. These results highlight that the role of PRDX2 in human hepatocytes and transformed cancer cells is different and that the direct antitumor effect is not mediated by AMPK (Figure 7, A and B). However, the impact of *PRDX2* KO on ERK activation seems to be consistent in all our models (Figures 4 and 7). Finally, *PRDX2* KO reduced the expression of the CD44 stem cell marker in tumors, supporting the role of PRDX2 in stemness and tumor initiation (Supplemental Figure 16).

**Figure 8:**
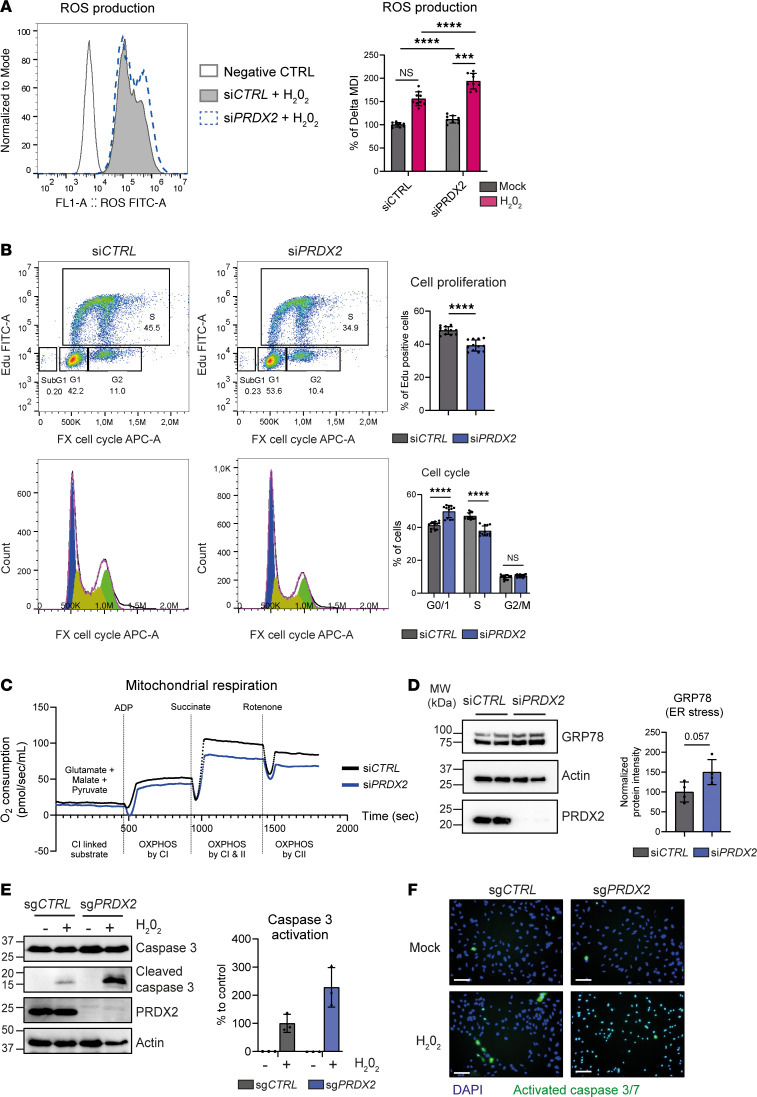
Targeting PRDX2 in cancer cells increases oxidative stress, reduces cell proliferation, and sensitizes cancer cells to apoptosis. (**A**) *PRDX2* KD increases ROS production in Huh7 cells. ROS generation was measured in Huh7 cells upon oxidative stress induced by H_2_0_2_ (300 μM, 4 hours), by flow cytometry. One representative histogram is shown. The graph shows mean ± SD of the percentage of delta mean of fluorescence from 3 independent experiments performed in triplicate (*n* = 9). ***P < 0.001; ****P < 0.0001 (ordinary 1-way ANOVA followed by Tukey’s multiple-comparison test). (**B**) *PRDX2* KD reduces cell proliferation. Huh7 cell proliferation and cell cycle profile were assessed by flow cytometry and costaining with Edu (cell proliferation) and Fx cell cycle (total DNA). One representative example of cell cycle profile is shown (blue = G_0_/G_1_; yellow = S, and green = G_2_/M). The graphs show mean ± SD of the percentage of proliferative cells (Edu^+^ cells) and of the percentage of cells in the different cell cycle steps of 3 independent experiments performed in 4 replicates (*n* = 12). ****P < 0.0001 (Mann-Whitney *U* test). (**C**) *PRDX2* KD impairs mitochondrial function. Mitochondrial respiration in Huh7 cells was assessed in a 2-chamber respirometer Oroboros Oxygraph-2k at 37°C. The graph shows time course of oxygen consumption upon successive activation/inhibition of the different mitochondria complexes. One representative experiment out of 3 is shown. (**D**) *PRDX2* KD increases ER stress. ER stress was assessed by Western blot analysis of the GRP78 marker. The graph shows mean ± SD of normalized protein intensity (normalization to total proteins) of 2 independent experiments performed in duplicates (*n* = 4). Exact *P* value is indicated (Mann-Whitney *U* test). (**E** and **F**) Targeting *PRDX2* sensitizes cancer cells to apoptosis upon oxidative stress (H_2_O_2_ 300 μM, 6 hours). Apoptosis was assessed by (**E**) Western blot analysis of caspase-3 and cleaved caspase-3 (graph shows mean ± SD of normalized protein intensity — normalization to total proteins — of 3 independent experiments, *n* = 3) and (**F**) by detecting activated caspase-3/7 using CellEvent caspase-3/7 detection reagent. Activated caspase is shown in green. Nuclei were counterstained in blue (DAPI). Scale bar: 200 μm. One representative experiment out of 3 is shown.

Finally, we performed perturbation studies in Huh7 cancer cells. As expected, a decrease in *PRDX2* expression led to an increase in oxidative stress and ROS production (Figure 8A) associated with a reduction in cell proliferation and cell division and a reduced invasive capacity (Figure 8B and Supplemental Figures 17, A–D). As a consequence of oxidative stress, we observed impairment of mitochondrial functions (Figure 8C) and an increase in ER stress (Figure 8D) but no impact on DNA damage (Supplemental Figure 17B). Given that Huh7 cells express a mutated and nonfunctional p53 protein, these effects are most likely p53 independent (49). Furthermore, Western blot analysis and fluorescence assay showed that *PRDX2* loss of function led to an increase in activated caspase-3 levels upon oxidative stress induced by H_2_O_2_ (Figure 8, E and F), indicating that targeting PRDX2 may help to restore sensitivity to oxidative stress and programmed cell death in tumors. These results highlight, again, the different mechanisms of action that are at play in cancer cells and hepatocytes, in which depletion of *PRDX2* has no impact on cell viability (Figure 2E and Supplemental Figure 6). The difference may be due to absence of compensation by other antioxidant systems in cancer cells compared with hepatocytes (Supplemental Figure 18).

**Figure 9:**
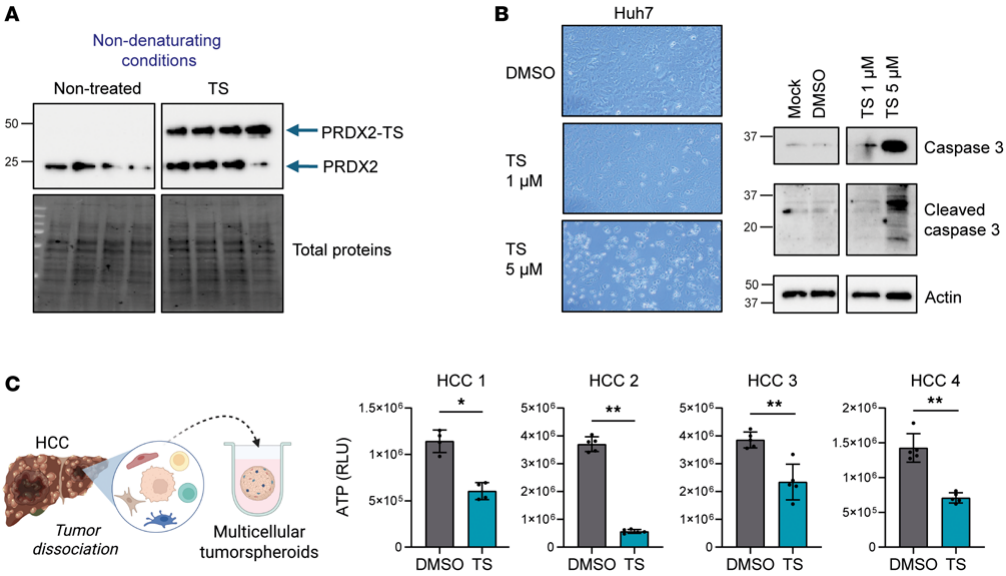
Targeting PRDX2 using thiostrepton induces Huh7 cancer cell apoptosis. (**A**) Validation of the effect of thiostrepton (TS) on PRDX2 in Huh7 by using nondenaturating PAGE after short-term TS treatment (4 hours). The gel shows the binding of TS on PRDX2. (**B**) Apoptosis was assessed by microscopy and Western blot analysis of caspase-3 and cleaved caspase-3 after TS treatment (24 hours). One representative experiment out of 3 is shown. (**C**) TS decreases HCC cell viability in a 3D patient-derived tumor spheroid model. HCC spheroids were generated from 4 patient HCC tissues with different etiologies. Cell viability was assessed 3 days after treatment by measuring ATP levels (RLU, relative light unit). Each experiment shows mean ± SD of RLU in treated spheroids. HCC 1, *n* = 4; HCC 2 to 5, *n* = 5. *P < 0.05; **P < 0.01 (Mann-Whitney *U* test).

To confirm these results by pharmacological intervention, we assessed the effect of the PRDX inhibitor thiostrepton, which did not show toxicity for PHHs (Figure 4), on cancer cell viability. Thiostrepton bound to PRDX2 and impaired its enzymatic activity (Figure 9A) (50), leading to apoptosis in Huh7 cells (Figure 9B). Finally, thiostrepton induced cancer cell death in multicellular patient-derived tumor spheroids generated from authentic HCC tumors (Figure 9C), confirming that PRDX2 plays a role in apoptosis resistance in HCC.

## Discussion

In this study, we identified PRDX2 as a therapeutic target for HCC chemoprevention. By combining computational analyses and animal and cell-based models, we demonstrate that (a) PRDX2 expression is associated with HCC in patients, (b) targeting PRDX2 in hepatocytes prevents HCC development in MASH by improving lipid metabolism and regulating procarcinogenic pathways, and (c) PRDX2 is involved in tumor initiation and progression.

Previous studies have shown that *PRDX2* expression is associated with the development of different solid cancers, including colorectal cancer (51, 52), lung adenocarcinoma (53), cervical cancer (54), ovarian cancer (55), and gastric cancer (56). Overexpression of *PRDX2* may either promote cancer growth or inhibit cancer development depending on the cancer context and the different mutations cooccurring in the cancer cells, among which are the melanomas (57). However, the role of PRDX2 in HCC development was unclear. Our study demonstrated that hepatocyte PRDX2 plays a multifaceted role in HCC development by regulating oxidative stress, lipid metabolism, cell cycle, procarcinogenic pathways, and apoptosis. Our results are in line with different studies showing that KD/low expression of *PRDX2* suppresses proliferation and induces senescence in HCC (25, 26). Zhang et al. also found high levels of secreted PRDX2 in patients with HCC and suggested that secreted PRDX2 may be used as an HCC biomarker (27). However, other studies were recently published with different conclusions by showing that high expression of *PRDX2* is associated with good prognosis in patients (23, 24). The discrepancy between these studies may be explained by heterogeneity in *PRDX2* expression among patients, a differential *PRDX2* expression during disease stage, and several factors such as disease etiology, HCC grade, immune cell infiltration, and cooccurrence of mutations in patients (22). Another possibility is that, while PRDX2 may act as a procarcinogenic factor early in carcinogenesis, its expression in later stages of HCC may be decreased by intensive and persistent oxidative stress in aggressive tumors.

Although we demonstrated that PRDX2 plays a role in HCC development in MASH and in HCC progression across our models, more investigations are needed to make conclusions about the driver role of PRDX2 in HCC. Alteration of *PRDX2* expression in liver disease and HCC may be a bystander effect due to progressive increase in oxidative stress that exacerbates cell damage and transformation. In addition, the potential mutations in the *PRDX2* gene in HCC are not documented (58).

Our study also indicates that the role of PRDX2 in hepatocytes and cancer cells is different. In mouse livers, we observed that *Prdx2* KO improves mitochondrial function and lipid metabolism through an AMPK-dependent mechanism. AMPK can be activated by different stimuli, including nutrient deprivation and exposure to hydrogen peroxide (59). Our mechanistic data suggest that targeting PRDX2 in hepatocytes restores AMPK function by increasing the level of peroxides, as it was demonstrated for other pathologies (60). In contrast, *PRDX2* KO in cancer cells induces mitochondrial and ER stress, leading to reduced cell proliferation and an increase in apoptosis upon oxidative stress in an AMPK-independent manner. The difference may be due to a different balance of antioxidant/oxidant in the two cell types. The effect of antioxidants in MASH is highly dependent on the disease stage. At the beginning of metabolic dysfunction–associated steatotic liver disease (MASLD) development, antioxidants are protective. However, in advanced stages, antioxidants generally exacerbate the damage and promote disease development. Finally, antioxidant supplements promote tumor formation and growth by reducing intracellular ROS (61, 62).

Our study reveals that targeting PRDX2 in hepatocytes may be a candidate strategy for HCC prevention. Although previous studies have shown that *Prdx2* homozygous null mice have anemia and enlarged spleens, due to the major antioxidant role of PRDX2 in erythrocytes, no liver or kidney toxicity was reported (63). In line with these observations, *Prdx2* KO and KD in vivo did not result in major detectable liver toxicity, indicating that targeting PRDX2 in patients may be well tolerated.

Our findings demonstrate that the role of PRDX2 in HCC is independent from fibrosis. Since 38% of HCCs develop in nonfibrotic liver in patients with metabolic dysfunction–associated fatty liver disease (MAFLD) (3), this finding may open perspectives for the development of HCC chemopreventive strategies. Our conclusion is supported by a chemopreventive effect observed in mice treated with GalNac siRNA targeting *Prdx2* expression. However, more investigations will be needed to adjust GalNac siRNA treatment (timing, number of injections per week, doses) for therapeutic use and its translation into patients in future studies. With the global epidemic of obesity, MAFLD/MASH-induced HCC is sharply increasing as a high-risk condition for HCC. Besides ongoing efforts to reduce obesity and metabolic disorders, HCC chemoprevention in at-risk patients may have a significant impact on the poor HCC prognosis (64).

## Methods

For reagents, primers, antibodies, and other resources, see Supplemental Table 4 and Supplemental Methods.

### Sex as a biological variable

Human liver samples from both females and males were involved in this research. Sex was not considered as a biological variable for human samples. To study liver disease and HCC development in vivo, we exclusively examined male mice because they developed more severe liver fibrosis compared with female mice. Moreover, liver cancer is more prevalent in men. It is unknown whether the findings are relevant for female mice. For the CDX immunocompromised model, both sexes were used.

### Study participants

RNA-Seq data from patients were obtained from publicly available datasets. The following public databases were used in the study: HBV-related liver cancer patient cohort (GEO GSE14520) (65), HCV-related liver cancer patient cohort (GEO GSE20140) (66), and The Cancer Genome Atlas (67) patient cohort. Representative liver sections from patients with HCC stained with PRDX2 antibodies and PHHs were obtained from patients undergoing liver resection with informed consent from all patients for deidentified use at the Institute for Translational Medicine and Liver Disease (ITM) Strasbourg, France (DC-2016-2616 and RIPH2 LivMod IDRCB 2019-A00738-49, ClinicalTrials.gov NCT04690972). The protocols were approved by the local Ethics Committee of the University of Strasbourg Hospitals. All material was collected during a medical procedure strictly performed within the medical treatment of the patient. Informed consent is provided according to the Declaration of Helsinki. Detailed patient information and informed consent procedures are implemented by the Strasbourg University Hospital Biological Resources Center (HUS CRB).

### Research experiments on live vertebrates

C57BL/6J mice were purchased from Charles River Laboratories. NRG (NOD.Cg-*Rag1^tm1Mom^Il2rg^tm1Wjl^*/SzJ), Alb-Cre (B6.Cg-*Speer6-ps1^Tg(Alb-cre)21Mgn^*/J), and Cas9 (B6J.129(B6N)-*Gt*(ROSA)26*Sor*^tm1(CAG-cas9*,-EGFP)Fezh/^J) mice were purchased from The Jackson Laboratory.

#### MASH-HCC mouse model.

Alb-Cre and Cas9 mice were crossed to generate the Alb-Cre/Cas9 F1 generation. Only male mice from the F1 generation were used in subsequent experiments. AAV8-sgCtrl and AAV8-sg*Prdx2*-3 (1 × 10^11^ genome equivalent per mouse) were delivered through intravenous injection in 4- to 5-week-old Alb-Cre/Cas9 mice. Three weeks later, mice were intraperitoneally injected with 100 mg/kg DEN, and after 1 week they were fed a CDA-HFD (Research Diets, A06071302) for 6 months. At the time of euthanization, animals were anesthetized, weighed, and a terminal blood collection was performed by cardiac puncture. The plasma fraction was kept at –80°C until further analyses. Livers were weighed, fixed in formalin or mounted in optimal cutting temperature (OCT) compound, and kept at –80°C until further analyses.

#### Prdx2 KD using GalNAc technology.

The following siRNA sequences were designed to target mouse *Prdx2* or used as nontargeting siRNA (control): GalNac *Prdx2*: 5′-AAAUCAAGCUUUCGGACUATT-3′; siRNA CTRL: 5′-UGGUUUACAUGUCGACUAATT-3′. siRNAs were then coupled to GalNac group (MicroSynth) and 3′-overhang dTdT groups for hepatocyte in vivo delivery. To validate the efficacy of GalNac siRNA, Hepa 1.6 mouse cells were transfected with 12 pmol of regular siRNAs or GalNac siRNAs by using lipofectamine RNAi Max (Invitrogen, 13778-150) following the manufacturer’s instructions. KD was validated by qRT-PCR (see Supplemental Figure 14). Eight-week-old C57BL/6J mice were injected with 100 mg/kg DEN, and after 1 week they were fed with CDA-HFD (Research Diets, A06071302) for 12 weeks. Then, they were intraperitoneally injected weekly with 3 mg/kg of GalNac siRNAs for 12 weeks. At the time of euthanization, animals were anesthetized, weighed, and a terminal blood collection was performed by cardiac puncture. The plasma fraction was kept at –80°C until further analyses. Livers were weighed, fixed in formalin or mounted in OCT compound, and kept at –80°C until further analyses.

#### CDX mouse model.

Six- to eight-week-old NRG mice were subcutaneously transplanted with 5 × 10^6^ of either Huh7.5.1_Cas9-sg*CTRL* or Huh7.5.1_Cas9-sg*PRDX2* cells and monitored weekly for tumor growth for 5 weeks (males and females). Tumor volume was determined by caliper measurement and calculated using the formula (L × W^2^) × 0.5, where L and W represent length and width of the tumors, respectively.

### Candidate HCC chemopreventive target prediction in human tissues

The candidate targets for HCC chemoprevention were predicted as described previously (9). Functionally coregulated gene modules in human fibrotic/cirrhotic liver tissues were determined in genome-wide transcriptome profiles of 523 patients with cirrhosis from 3 independent patient cohorts reported in previous studies (cohort 1: 82 Japanese patients with HCCs with mixed etiologies [GSE10140], cohort 2: 225 European and US patients with HCCs with mixed etiologies [GSE10142], and cohort 3: 216 European patients with early-stage HCV-related cirrhosis [GSE15654]) (9). Gene coexpression was first determined in each dataset by using random permutation-based correlation testing, and resulting nominal *P* values were synthesized using Fisher’s inverse χ^2^ statistic (ICS). The significance of Fisher’s ICS was evaluated based on its null distribution generated by iterative random resampling of the nominal *P* values from the correlation test (*n* = 10,000) and adjusted by Benjamini-Hochberg FDR. Subsequently, using gene-gene pairs with significant connection (FDR < 0.05), cirrhosis regulatory gene modules were determined by using the PFNA algorithm (9), which identifies tightly coregulated multiscale gene subnetworks that fulfill the biological scale-free property. Functional annotation of each PFNA gene module was performed by a hypergeometric test using a comprehensive collection of 10,295 annotated gene sets in the Molecular Signature Database (68). Key regulatory genes in each gene module were determined by key driver analysis (16), which prioritizes driver genes by measuring the impact on the downstream genes such that the downstream genes were defined by *n*-layer neighborhood in a coexpression network with optimal n that maximizes the enrichment statistic. The raw data can be found in the NCBI GEO (GSE64520).

### Candidate HCC chemopreventive target prediction in the cPLS system

The candidate targets for HCC chemoprevention in the cPLS system were predicted as described (14). To infer the regulatory networks underlying the PLS induction, 21,950 genes from the RNA-Seq time course data were analyzed (GSE126831) using the AMARETTO algorithm (69). First, AMARETTO starts by selecting the top 50% most varying genes across the samples in an unsupervised manner. A predefined list of candidate regulators was included in the analysis. The AMARETTO algorithm subsequently identified regulators as those putatively controlling the target genes in 150 modules of coexpressed target genes genome-wide using regularized regression. These modules were assessed for their enrichments in known functional categories from the Molecular Signatures Database Hallmark and C2CP Collections (68, 70). AMARETTO’s source code in R is available from GitHub (https://github.com/gevaertlab/AMARETTO). The algorithm subsequently identified a list of candidate regulators by connecting known regulatory driver genes with coexpressed target genes. The list was narrowed down to 30 candidates according to (a) high expression in liver tissues, (b) association with liver disease in patients, and (c) ability of the encoded protein to be targeted by drugs.

### Generation of PRDX2 WT and mutant C51S for rescue experiments

The human *PRDX2* sequence carrying 6 silent mutations in the sgRNA *PRDX2* target sequence and mutation in the codon for cysteine C51 for substitution to S51 (TGC > AGC) were purchased from IDT DNA (see Supplemental Methods for the complete sequences). DNA was amplified by PCR using Platinum Taq DNA Polymerase, High Fidelity (Invitrogen) following the manufacturer’s instructions and the primer sequences 5′-aaaaACTAGTGCCGCCATGGCCTCCGGTAAC-3′ and 5′-aaaaGTTTAAACTTACTAATTGTGTTTGGA-3′. Amplified DNA fragment was cloned in pLenti-puro plasmid (Addgene, 39481) (71) for reexpression of the WT PRDX2 resistant to sgRNA *PRDX2* or the C51S mutant resistant to sgRNA *PRDX2* in Huh7.5.1 cancer cell KO for *PRDX2* using lentiviral vectors (see Supplemental Methods).

### Clonogenic and tumor spheroid assays

A total of 5,000 cells per well in a 6-well plate format were seeded in complete DMEM supplemented with 1× B-27 Supplement (Gibco) and FGF and EGF (20 ng/mL each; PeproTech) in regular plates for clonogenic assay or low attachment plates for tumor spheroid assay (Corning). After 10 days, colonies were stained using crystal violet, and the number of colonies was assessed using Celigo Image Cytometer (Nexcelcom Biosciences). The number of tumor spheres was assessed an average of 20 days after seeding.

### Patient-derived tumor spheroids

Patient-derived tumor spheroids were generated from patient HCC liver tissues undergoing surgical resection as described in Crouchet et al. (72). Briefly, HCC tissues were dissociated using a Human Tumor Dissociation kit (Miltenyi Biotec), following the manufacturer’s instructions. Total cell populations, including epithelial (i.e., cancer cells/hepatocytes) and non-parenchymal cells, were used to generate multicellular tumor spheroids in a Corning 96-well black/clear bottom low flange ultra-low-attachment microplate in complete Mammocult medium supplemented with 20% of patient autologous serum. After tumor spheroid formation, HCC-derived spheroids were treated with 5 μM thiostrepton or DMSO vehicle control for 3 days. Cell viability was assessed using CellTiter-Glo (Luminescent Cell Viability assay), according to the manufacturer’s instructions.

### Statistics

For in vivo experiments, the sample size estimate was based on a *P* value of 0.01 at 90% power assuming a 50% difference in means in tumor burden with 33% standard deviation between control and drug-treated animals. In vitro and in vivo data are presented with mean ± SD. Two-tailed Student’s *t* test, 2-tailed Mann-Whitney *U* test, ordinary 1-way ANOVA test followed by Tukey’s multiple-comparison test, or Kruskal-Wallis test followed by Dunn’s multiple-comparison test were performed after sample distribution was determined with the Shapiro-Wilk normality test. All in vitro experiments were performed at least in triplicate and repeated 3 times, except for PHH (2 times) and HCC tissues (*n* = 1, repeated for several patients) due to the rarity of the samples. In vivo and in vitro experiments were considered significant at *P* less than 0.05. Statistical analyses were performed with GraphPad Prism 10.4.1 software.

### Study approval

Mice breeding and experiments were carried out at the INSERM UMR_S1110 animal facility Animal Experimentation Platform Infection and Cancer (approval F-67-482-7). The experiments are compliant with the relevant ethical regulations regarding animal research and were reviewed and approved by the local ethical committee and authorized by the French Ministry of Research and Higher Education (authorization APAFIS 29390-2021012912304998_v3, 27709-2020101514256404_v4, and 43242-2023050317311826_v2). Patient samples were obtained from patients undergoing liver resection for deidentified use at the Institute for Translational Medicine and Liver Disease (ITM), Strasbourg, France (DC-2016-2616 and RIPH2 LivMod IDRCB 2019-A00738-49, ClinicalTrials.gov NCT04690972). Written informed consent was provided according to the Declaration of Helsinki. The patients maintain the right to withdraw their consent at any time and to request the destruction of their biological material, which is strictly respected. While there was clinical descriptive data available, the identity of the patients was protected by internal coding. The protocols were approved by the local Ethics Committee of the University of Strasbourg Hospitals.

### Data availability

The authors declare that the data supporting the findings of this study are available within this article, its supplemental information, and the Supporting Data Values file. RNA-Seq data were deposited in the NCBI’s GEO database with the accession number GSE199320. GSEA of the RNA-Seq data are available in Supplemental Table 3. Further information and requests for resources and reagents should be directed to Catherine Schuster (catherine.schuster@unistra.fr) and Emilie Crouchet (ecrouchet@unistra.fr). The illustrations presented in Figure 1A, Figure 2A, Figure 5A, Figure 6E, Figure 9C, and the graphical abstract were generated using BioRender (https://BioRender.com/9vjy72c).

## Author contributions

EC, CS, and TFB initiated and coordinated the study. EC, ES, LM, MH, JZ, YH, TFB, and CS designed experiments and analyzed the data. EC, ES, MAO, CG, SCD, AC, CP, RM, NB, LMH, MP, DH, JH, and LM performed experiments and/or analyzed data. JM, FJ, HES, NF, SZ, and FAR performed bioinformatic analyses. NP and YH performed and supervised the computational analyses. LM supervised animal experiments. NH participated in implementation of the CRISPR technology. EF and PP provided patient liver tissues. BC provided PRDX2 inhibitor and shared the procedure for determination of PRDX2 conformation. FDZ performed pathology analyses. EC, ES, LM, CS, and TFB wrote the manuscript.

## Funding support

This work is the result of NIH funding, in whole or in part, and is subject to the NIH Public Access Policy. Through acceptance of this federal funding, the NIH has been given a right to make the work publicly available in PubMed Central.

- This work was supported by the European Union (ERC-AdG-2020-FIBCAN 101021417 to TFB and YH, EU H2020-667273-HEPCAR to TFB and MH; Agence nationale de recherches sur le sida et les hépatites virales, Paris 2013/108 and ECTZ103701 to TFB); ARC Foundation Paris and IHU Strasbourg (TheraHCC2.0 IHUARC2019 to TFB); NIH (CA233794, CA255621, CA282178, CA288375, and CA283935 to YH; CA209940, R21CA209940, and R03AI131066 to NP and TFB); the Foundation of the University of Strasbourg (to TFB); Cancer Prevention and Research Institute of Texas (RR180016 and RP200554 to YH); the Alsace Cancer Foundation (to EC, TFB, and CS); and the Institut Universitaire de France (to TFB).
- NF is supported by JSPS KAKENHI grant 24K11130 and Agency for Medical Research and Development under grants 25fk0210177 and 25fk0310537.
- MH is supported by an European Research Council Consolidator Grant (HepatoMetaboPath) and Excellence of Science grant and by the Deutsche Forschungsgemeinschaft (DFG, German Research Foundation) project ID 272983813–TRR 179 and project ID 314905040 SFB TR209.
- This work has been published under the framework of the LABEX ANR-10-LABX-0028_HEPSYS and INSERM Plan Cancer and benefits from funding from the French state funds managed within the “Plan Investissements d’Avenir” and by the Agence nationale de la recherche (ANR) (references ANR-10-IAHU-02 and ANR-10-LABX-0028), along with French state funds managed by the ANR within the France 2030 program (reference ANR-21-RHUS-0001 DELIVER).
- The Animal Experimentation Platform Infection and Cancer was supported by University of Strasbourg IdEX 2024 Dispositifs plateformes (RDGGPJ2403M).
- This work of the Interdisciplinary Thematic Institute IMCBio, as part of the ITI 2021-2028 program of the University of Strasbourg, CNRS, and INSERM, was supported by IdEx Unistra (ANR-10-IDEX-0002) and by SFRI-STRAT’US project (ANR 20-SFRI-0012) and EUR IMCBio (ANR-17-EURE-0023) under the framework of the French Investments for the Future Program.
- Lukas Baumert participated in the early stage of the project during his internship as a student of the Rotteck-Gymnasium Freiburg and the “Jugend Forscht” program.

## Supplementary Material

Supplemental data

Unedited blot and gel images

Supplemental table 3

Supporting data values

## Figures and Tables

**Table 1:**
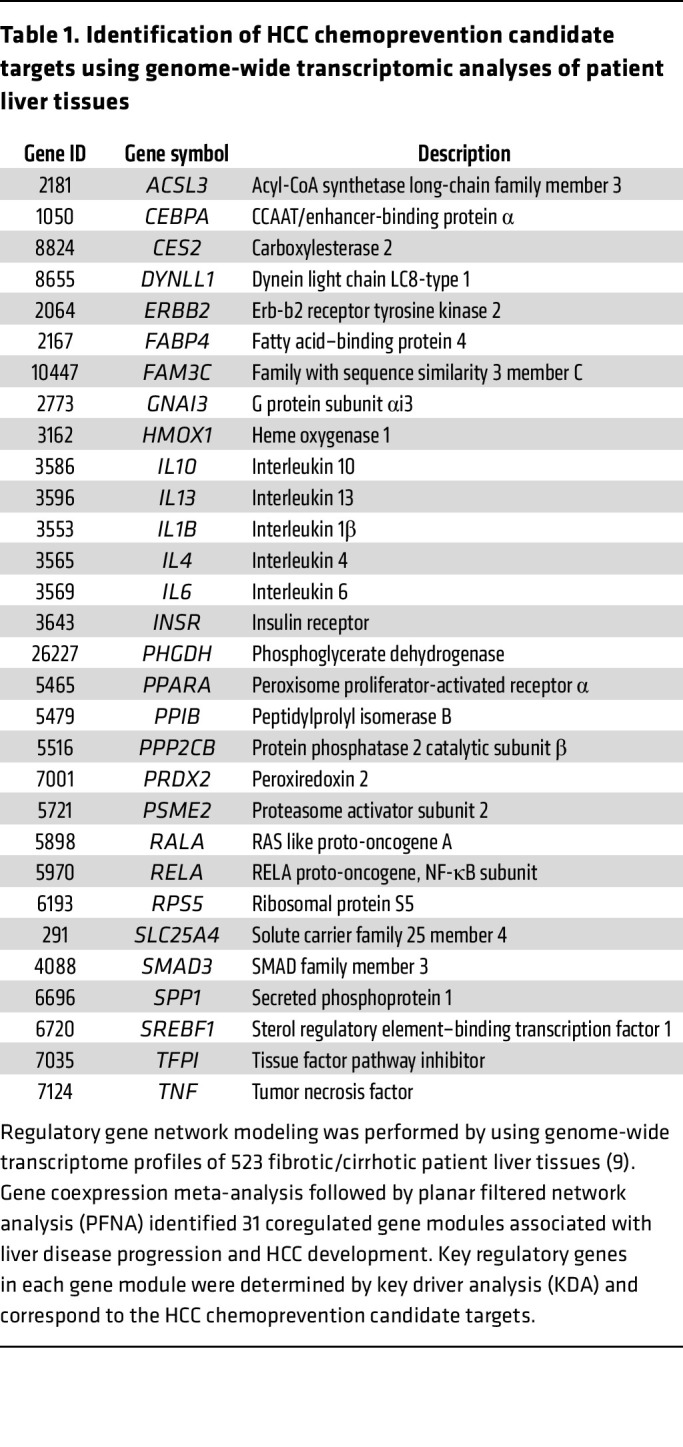
Identification of HCC chemoprevention candidate targets using genome-wide transcriptomic analyses of patient liver tissues

**Table 2:**
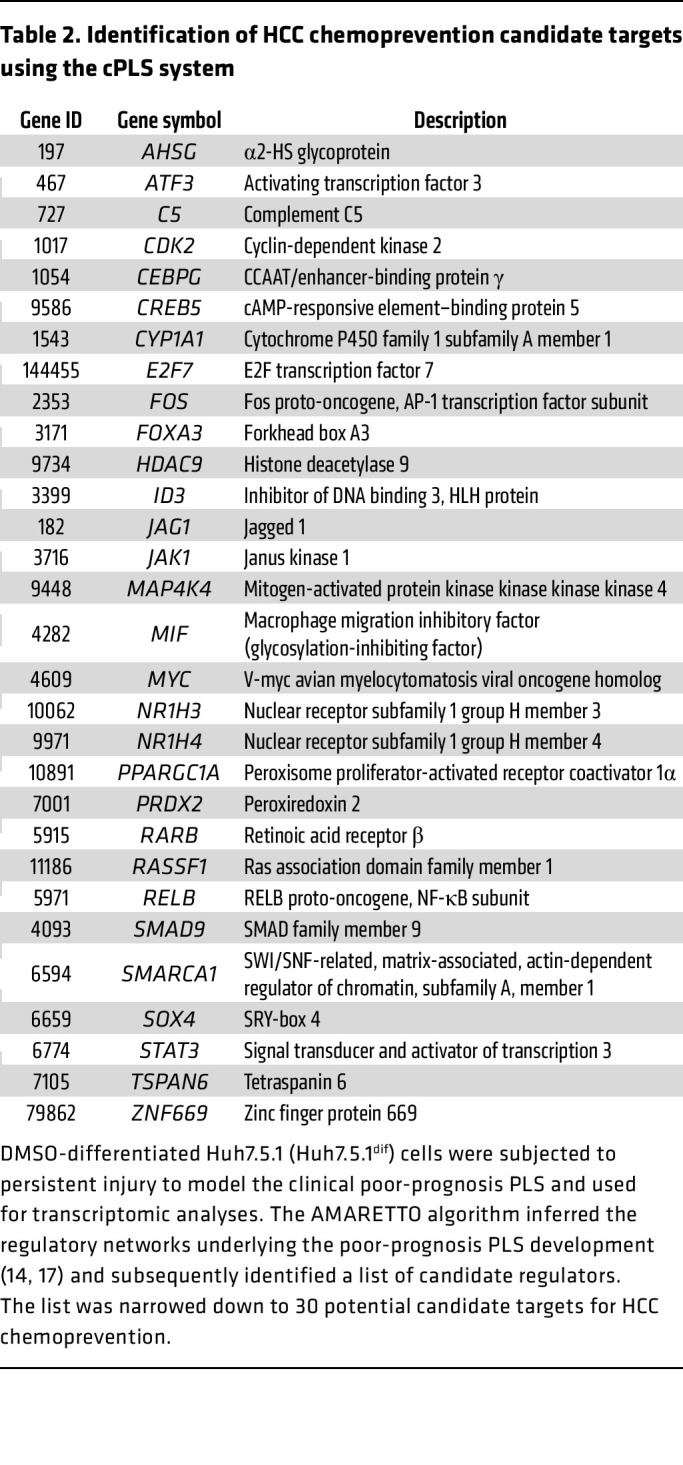
Identification of HCC chemoprevention candidate targets using the cPLS system

## Version 1 09/11/2025

In-Press Preview

## Version 2 11/03/2025

Electronic publication
