# Supplementary material for: Targeting peroxiredoxin 2 prevents hepatocarcinogenesis in metabolic liver disease models
Source: J Clin Invest. 2025 Sep 11;135(21):e169395. doi: 10.1172/JCI169395 (PMC12578407; doi:10.1172/JCI169395)
Supplement: Supplemental data [file jci-135-169395-s246.pdf]

Targeting peroxiredoxin 2 prevents hepatocarcinogenesis in metabolic liver disease models

Emilie Crouchet*$[1], Eugénie Schaeffer*[1], Marine A Oudot[1], Julien Moehlin[1], Cloé Gadenne[1], Frank Jühling[1], Hussein El Saghire[1], Naoto Fujiwara[2,3], Shija Zhu[2], Fahmida Akter Rasha[2], Sarah C Durand[1], Anouk Charlot[1,4], Clara Ponsolles[1], Romain Martin[1], Nicolas Brignon[1], Fabio del Zompo[1], Laura Meiss-Heydmann[1], Marie Parnot[1], Nourdine Hamdane[1], Danijela Heide[5], Jenny Hetzer[5], Mathias Heikenwälder[5,6], Emanuele Felli[1,7,8], Patrick Pessaux[1,9], Nathalie Pochet[10,11], Joffrey Zoll[3,12,13], Brian Cunniff[14], Yujin Hoshida[2], Laurent Mailly[1], Thomas F. Baumert$[1,15,16], Catherine Schuster$[1].

[1]University of Strasbourg, Inserm, Institute for Translational Medicine and Liver Disease (ITM), UMR_S1110, Strasbourg, France; [2]Liver Tumor Translational Research Program, Simmons Comprehensive Cancer Center, Division of Digestive and Liver Diseases, Department of Internal Medicine, University of Texas Southwestern Medical Center, Dallas, TX, USA ; [3]Department of Gastroenterology and Hepatology, Mie University, Mie, Japan; [4]University of Strasbourg, Biomedicine Research Center of Strasbourg (CRBS), UR 3072 Mitochondrion, Oxidative Stress and Muscle Plasticity, Strasbourg, France; [5]Division of Chronic Inflammation and Cancer, German Cancer Research Center, Heidelberg, Germany; [6]Cluster of Excellence iFIT (EXC 2180) "Image-Guided and Functionally Instructed Tumor Therapies," Eberhard-Karls University of Tübingen, Tübingen, Germany; [7]Hospital Group Saint Vincent, Strasbourg, France; [8]Liver Transplant and Surgery Department, Trousseau Hospital, Tours, France. [9]Unit of Hepato-Bilio Pancreatic Surgery, Department of Visceral and Digestive Surgery, Nouvel Hospital Civil, University Hospital of Strasbourg, Strasbourg, France. [10]Broad Institute of Harvard and Massachusetts Institute of Technology, Cambridge, MA, USA; [11]Department of Neurology, Harvard Medical School, Boston, MA, USA; [12]Service

de Physiologie et explorations fonctionnelles, University Hospital of Strasbourg, Strasbourg, France ;[13]Faculty of Medicine, University of Strasbourg, Strasbourg, France; [14]University of Vermont Cancer Center, Larner College of Medicine, Department of Pathology and Laboratory Medicine, Burlington, VT USA; [15]Gastroenterology and Hepatology Service, Strasbourg University Hospitals, Strasbourg, France; [16]Institut Universitaire de France (IUF), Paris, France.

* These authors contributed equally

$ corresponding authors

Table of contents

SUPPLEMENTARY MATERIAL AND METHODS: 4

SUPPLEMENTARY FIGURES: 13

Supplementary Figure 1 (related to Figure 1C-E): Single cell RNA-Seq analysis of normal and cirrhotic patient livers. 14

Supplementary Figure 2 (related to Figure 1F): PRDX2 expression in HCC patient tissues. 15

Supplementary Figure 3 (related to Figure 1): PRDX2 is overexpressed in HCC derived cell lines (Huh7) compared to primary human hepatocytes (PHH). 16

Supplementary Figure 4 (related to Figure 1): Co-regulatory cirrhosis gene module associated with elevated HCC risk in cirrhotic patients 17

Supplementary Figure 5 (related to Figure 2A). Selection of single guide RNA (sgRNA) targeting mouse *Prdx2* in Hepa 1-6 Cas9 cells. 18

Supplementary Figure 6 (related to Figure 2). *PRDX2* knock-down (KD) in primary human hepatocytes (PHH) showed no cytotoxic effect. 19

Supplementary Figure 7 (related to Figure 2B). *Prdx2* KO does not significantly affect expression of the other *Prdx* family members in mouse livers. 20

Supplementary Figure 8 (related to Figure 3B): *Prdx2* KO decreases CD44 expression in a MASH/HCC mouse model 21

Supplementary Figure 9 (related to Figure 3C): *Prdx2* KO decreases pro-inflammatory cytokine secretion in a MASH/HCC mouse model. 22

Supplementary Figure 10 (related to Figure 3D). Effect of *Prdx2* KO on p53 expression. 23

Supplementary Figure 11 (related to Figure 3): PRDX2 expression and conformation in mouse livers. 24

Supplementary Figure 12 (related to Figure 3): PRDX2 expression and conformation in human livers. 25

Supplementary Figure 13 (related to Figure 4C). Inhibition of PRDX2 decreases STAT3 activation in PHH 26

Supplementary Figure 14 (related to Figure 5A): Validation of GalNac siRNA targeting *Prdx2* efficacy....... 27

Supplementary Figure 15 (related to Figure 5B-C): AMPK expression, activation and lipid accumulation in mouse livers treated with GalNac siRNAs. 28

Supplementary Figure 16 (related to Figure 6E): *PRDX2* KO decreases CD44 expression in a CDX mouse model.. 29

Supplementary Figure 17 (related to Figure 8A-B): Role of PRDX2 in cancer development and progression. 30

Supplementary Figure 18 (related to Figures 8E-F): Expression of the main antioxidant defense systems in mouse livers and in *PRDX2* KO cancer cells. 31

SUPPLEMENTARY TABLES: 32

Supplementary Table 1 (related to supplementary Figure 1): Clinical information of the HCC cases. 32

Supplementary Table 2 (related to Figure 1H): The 32 gene PLS. 33

Supplementary Table 3 (related to Figure 3): Gene set enrichment analysis (GSEA) of RNA-Seq from mouse liver tissues (MASH/HCC mouse model). Refer to the excel table 34

Supplementary Table 4: Reagents and resources 34

SUPPLEMENTARY REREFENCES: 38

SUPPLEMENTARY MATERIAL AND METHODS:

Cell lines

Huh7.5.1 cells are a gift from Dr. F. Chisari (The Scripps Research Institute, La Jolla, CA), Huh7 cells are a gift from Prof. G. Cristofori (University of Basel) and Hepa1.6 were purchased from ATCC. Huh7.5.1, Huh7 and Hepa1.6 cells were cultured in Dulbecco's Modified Eagle Medium (DMEM) supplemented with 10% heat-decomplemented fetal bovine serum, gentamycin (0.05 mg/mL) and non-essential amino acids (except for Hepa1.6 cells) at 37°C with 5% CO_2. For proliferation arrest and differentiation (Huh-7.5.1^{dif} cells), Huh-7.5.1 cells were cultured in complete DMEM supplemented with 1% DMSO for 10 days before seeding (1). The cell lines were certified mycoplasma free.

Primary human hepatocytes (PHH)

PHH were obtained from patients undergoing liver resection with informed consent from all patients for de-identified use at the Institute for Translational Medicine and Liver Disease (ITM), Strasbourg, France (DC-2016-2616 and RIPH2 LivMod IDRCB 2019-A00738-49, ClinicalTrial NCT04690972). PHH were isolated as described (1). Briefly, liver specimens were perfused for 15 minutes with calcium-free 4-(2-hydroxyethyl)-1-piperazine ethanesulfonic acid buffer containing 0.5 mM ethylene glycol tetraacetic acid (Fluka) followed by perfusion with 4-(2-hydroxyethyl)-1-piperazine ethanesulfonic acid containing 0.5 mg/mL collagenase (Sigma-Aldrich) and 0.075% CaCl2 at 37°C for 15 min. Then the cells were washed with phosphate-buffered saline (PBS) and nonviable cells were removed by Percoll® (Sigma-Aldrich) gradient centrifugation. PHH were then seeded on collagen type I coated plates (Corning®) in complete William Medium.

In vitro CRISPR/Cas9 gene editing

Lentiviruses expressing single guide RNA (sgRNAs) were generated by transient transfection of HEK 293T cells. HEK 293T cells were adjusted to a density of 2.10^5 cells/mL and 10mL were added to a 10 cm diameter Petri dish. The day after, the cell culture media was replaced. In parallel, 8.1 µg of PS-PAX2 (#12260 Addgene), 2.7 µg of pMD2.G (#12259 Addgene), 8.1 µg of pXPR-BRD016-PRDX2, 62 µL of 2M CaCl2 (Clontech) are mixed to nuclease free water to a final volume of 500 µL. The mix was dripped on 500 µL of HEPES-Buffer Saline (Clontech) a polypropylene hemolysis tube. This mix was left 20 minutes at room temperature and homogeneously added on the HEK 293T cells. Two days after transfection, the supernatants were harvested, filtered through 0.45 µm, aliquoted and stocked at -80°C until use. Huh7.5.1 stably expressing Cas-9 endonuclease (Huh7.5.1-Cas9) were generated by transduction of a lentiviral vector expressing Cas9 (pXPR_BRD111, Broad Institute). For *PRDX2* KO, Huh7.5.1-Cas9 cells were then transduced with lentiviruses expressing single guide RNA (sgRNA) CTRL targeting *GFP* (sg*CTRL*) or sgRNA targeting *PRDX2* designed by the Broad Institute. After 48 h, transduced cells were selected under hygromycin treatment (125 µg/ml). *PRDX2* KO was determined by Western blot analysis.

Production of AAV vectors for *in vivo* gene editing

Recombinant adeno-associated virus serotype 8 (AAV8-sg*Ctrl* and AAV8-sg*Prdx2*) were produced by *PEI*-mediated transient *transfection of* a HEK293T-derived cell line (293T/17) with pAAV-sg*CTRL* or pAAV-sg*Prdx2* expression plasmids and the pDP8.ape helper plasmid for serotype 8 (N°478, Plasmidfactory). Two days after transfection, AAV vectors were purified from cell lysate by Iodixanol gradient ultracentrifugation. Cells were harvested in 24 mL lysis buffer per *HYPERFlask cell culture vessel (Corning).* The lysate was subjected two freeze-thaw cycles in dry ice/ethanol and 37 °C water baths, further treated with 100 U/mL Benzonase

(Merck) for 1 hour at 37 °C and clarified by centrifugation at 3,000 x g for 15 min. AAV vectors were purified by Iodixanol gradient ultracentrifugation as described in OptiPrep™ Application Sheet V14 (Axis Shield). Viruses were dialyzed and concentrated against AAV formulation buffer (Dulbecco's PBS with 0.5 mM $MgCl_2$) using centrifugal filters (Amicon Ultra-15, 100 KDa cutoff) and finally filtered through 0.22 µm. Viral titers were determined by qPCR using LightCycler480 SYBR Green I Master (Roche) with primers 5'-GACGACGGCAACTACAAGA-3' and 5'-GTGGCTGATGTAGTTGTACTC-3'). The standard curve was performed with 10-fold serial dilutions of pAAV that was freshly denaturated in 200mM NaOH for 45 min at 65°C and neutralized by addition of an equimolar amount of HCl. AAV8 viruses were diluted to a final concentration of 1x 10^{13} viral genome per ml (vg/ml) and stored at −80°C until use.

HCV infection

Cell culture-derived HCVcc Jc1(2) were produced in Huh7.5.1 cells by electroporation of viral RNA. The cell culture supernatants were harvested every 3 days for a total of 12 days and were concentrated 10 times using Vivaspin 20, 10,000 MWCO PES (Sartorius). In parallel, Huh7.5.1 were subjected to the electroporation protocol without viral RNA and the supernatants were collected and concentrated in the same conditions (mock-electroporated cells). HCV infectivity was determined by calculating the Tissue culture Infective Dose 50 ($TCID_{50}$). To analyze the PLS induction, Huh7.5.1^{dif} cells were infected with HCV Jc1 (TCID 10^6 infectious particle /mL), for a total of 10 days (1). Cell culture supernatants from mock-electroporated cells (without viral RNA) were used for control experiments. PLS induction in virus-infected cells was always determined using mock-infected cells as control references.

PLS calculation

The PLS 32 gene expression profiling was performed using 250-500 ng total RNA by using nCounter Digital Analyzer system (NanoString). For the PLS 32 gene list, refer to **Supplementary Table 3**. PLS gene expression was normalized according to 6 housekeeping gene expression using GenePattern genomic analysis toolkits (3, 4). Detailed PLS gene expression profiles are presented as heatmaps showing the mean expression of the 32 PLS genes using Morpheus software (z scores of log2 normalized data). Induction or suppression of the PLS signature was determined as previously reported by using Gene Set Enrichment Analysis (GSEA), implemented in GenePattern genomic analysis toolkits (3, 4). PLS was always determined by using CTRL cells as references. Results are presented as simplified heatmaps showing the classification of PLS global status as poor or good prognosis and the significance of induction/suppression of PLS genes (log10 of false discovery rate (FDR) values). Global status corresponds to the difference between low risk- and high risk-gene expression. PLS induction in virus-infected cells was always determined using mock-infected cells as control references.

RNA sequencing analysis and data processing

Total liver RNA (500 ng) from 3 CDA-HFD sg*Ctrl* and 3 CDA-HFD sg*Prdx2* mice liver were submitted to Next Generation Sequencing (Biomedical Sequencing Facility, CEMM Research Center for Molecular Medicine of the Austrian Academy of Sciences, Vienna (Austria)). Data were analyzed as previously described in (5).

Gene expression analysis

Total RNA from mouse livers were obtained using Tri Reagent (MRC) and Direct-zol RNA Miniprep kit (Zymo Research). Total RNA from cells in culture were obtained using the

ReliaPrep™ RNA Miniprep Systems kit (Promega). RNA quantity and quality was assessed using Nanodrop. Total liver RNA (500 ng) was used for cDNA synthesis using Maxima H Minus Reverse Transcriptase (ThermoFisher), cDNA was then used for quantitative real-time qPCR with iTaq Universal SYBR Green Supermix (Biorad) or iTaq Universal Probes Supermix (Biorad). Relative gene expression was calculated using the $2^{-\Delta CT}$ method, using *GAPDH* or *18S* as reference genes. The references for the primers are indicated in **Supplementary Table 5**.

Wound healing assay

Huh7 cells were reversed transfected with siRNA targeting *PRDX2* expression or non-targeting *CTRL* using Lipofectamine RNAi max (Invitrogen) according to manufacturer's instructions. After 2 days, the cells were treated with mitomycine C 5 µg/mL (Sigma) to block cell proliferation. Wound healing assay was performed using Wound Healing Assay kit (Abcam) following manufacturer's instructions. Pictures were taken at 0 hour and 24 hours post-scratching with a camera-equipped microscope (Motic AE2000). Distance between wound edges was measured with Image J.

Invasion assay

Huh7 cells were reversed transfected with siRNA targeting *PRDX2* expression or non-targeting CTRL using Lipofectamine RNAi max (Invitrogen) according to manufacturer's instructions. For invasion, 100 µL of Culturex Basement Membrane Extract at 3,33 mg/mL in serum free media was added to the upper chamber of transwell (Corning Costar Transwell cell culture inserts) and incubated at 37°C for 30 minutes. After the gel was formed, cells were detached and resuspended in serum free medium and 100 000 cells were transferred to transwell inserts (24 well plate format). Then 600 µL of complete medium was added to the lower chamber.

After 24h of incubation at 37°C, the residual cells on the surface and the gel were removed. Then cells, were fixed in 70% ethanol and stained with 0,2% crystal violet. The invading cells were photographed with a camera-equipped microscope (Motic AE2000) and Colored Area was evaluated in five different fields using ImageJ.

Lipid staining

PHH were incubated with free fatty acids (FFA): 800 µM oleic acid and 400 µM palmitic acid for 48 h. Intracellular neutral lipids were stained with HCS LipidTOX™ Deep Red Neutral Lipid Stain (ThermoFisher) according to manufacturer's instructions. Nuclei were counterstained with DAPI. Fluorescent imaging was performed using an Axio Observer Z1 microscope and ZEN software (Carl Zeiss, Germany).

Mitochondrial respiration

Mitochondrial respiration was assessed in a two-chamber respirometer Oroboros Oxygraph-2k (O2k; Oroboros Instruments, Innsbruck, Austria) at 37 °C, and data were acquired and analyzed using DataLab software (Oroboros Instruments, Innsbruck, Austria). Huh7 cells were reversed transfected with siRNA targeting *PRDX2* expression or non-targeting CTRL using Lipofectamine RNAi max (Invitrogen) according to manufacturer's instructions. After 3 days, cells were harvested, centrifuged at 2000 RPM for 5 min (20 °C) and re-suspended in 100 µL of the 2,1 mL MirO5 buffer placed in the chamber (0.5 mM EGTA, 3 mM MgCl2, 60mM Potassium lactobionate, 20 mM Taurine, 10 mM KH2PO4, 20mM HEPES, 110 mM Sucrose and 2mg/ml BSA) and containing 0,125 mg/mL saponin for cell permeabilization. The final cells concentration in the O2k-chamber was 10^6 cell/mL. The adapted SUIT-011 Oroboros protocol was used for the respiration measurement. Malate (2 mM) and glutamate (10 mM) were added and Complex I-linked substrate state was measured. Then, ADP (2mM) were added

to measure Complex-I OXPHOS, Succinate (10mM) to measure Complex I & II OXPHOS, and finally Rotenone (0.5 µM) to measure Complex II OXPHOS.

Native gels

After TS treatment, cells were incubated in warm PBS supplemented with 100 mM S-Methyl Methanethiosulfonate (MMTS, Sigma) for 20 min at 37°C and lysed in RIPA buffer + protease inhibitor (150 mM NaCl, 1% NP-40, 0.25% Na-deoxycholate, 50 mM Tris-HCl pH7.4, 1 mM EDTA, qsp H_20) supplemented with 100 mM MMTS. Tissues were lysed in RIPA buffer + protease inhibitor + 100 mM MMTS. PRDX2 conformation was assessed by SDS-PAGE electrophoresis in native conditions (without denaturating buffer).

Stain free and Western blot quantification

To measure total protein levels in Western blot analysis, we used the Stain Free Imaging Technology (Biorad) according to manufacturer's instructions. Data integration was performed using Image Lab software (Biorad). All the quantifications of the protein intensity were normalized to total proteins.

Proliferation and cell cycle assay

Huh7 cells were reverse-transfected with siRNA targeting *PRDX2* expression or non-targeting *CTRL* using Lipofectamine RNAi max (Invitrogen) according to manufacturer's instructions. After 2 days, cells were synchronized by incubating the cell in serum free medium overnight and then adding complete medium + Edu at 10 µM for 4h. Cells were harvested, fixed and co-stained with Click-iT® Edu Flow Cytometry FITC kit (ThermoFisher) for cell proliferation and FxCycle™ Far Red Stain (ThermoFisher) for DNA content and cell cycle according to the manufacturer instructions. Flow cytometry was performed using the CytoFLEX cytometer

(Beckman Coulter) and analyses were performed with FlowJo Data analysis software (BD Biosciences).

Apoptosis assay

Huh-7.5.1-Cas9 cells *CTRL* or KO for *PRDX2* were treated with 300 µM H_2O_2 for 6h. Caspase-3/7 activation was detected by immunoblotting or using CellEvent® Caspase-3/7 Green Detection Reagent (ThermoFisher) following manufacturer's instructions and using Celigo Image Cytometer (Nexcelcom Biosciences).

Histology, immunohistochemistry, plasma biochemistry

Macroscopic tumor nodules were counted from livers and their size was determined on liver pictures using ImageJ. For the MASH/HCC mice, formalin-fixed samples were embedded in paraffin, cut in 2 µm sections and used for IHC. For hematoxylin and eosin (H&E) or Picrosirius Red, 5 µm sections were performed. For patient samples and GalNac mouse model, formalin-fixed samples were embedded in paraffin, cut in 5 µm sections for H&E, IHC and Picrosirius Red. For TUNEL staining, sections were stained with the TUNEL Assay Kit – HRP DAB (Abcam #206386) following the manufacturer instructions. OCT- mounted samples were cut in 10 µm sections and stained with Oil Red O. Glass slide-mounted tissues were scanned with Nanozoomer scanner (Hamamatsu), images were analyzed using image processing software (NDP.view.2) and quantification using Image J (Sirius red) or Qu-Path (IHC).

Hydroxyproline quantification

Hydroxyproline was quantified in frozen liver tissues from the diet-based MASH/HCC mouse model, using the Hydroxyproline Assay Kit (Sigma) following manufacturer's instructions. Briefly, between 12 to 21 mg of frozen liver tissues were sampled from each mouse (Chow diet

n = 6, DEN/CDA-HFD sg*Ctrl* n =18, DEN/CDA-HFD sg*Prdx2* n =16), homogenized in 100 µL of water and hydrolyzed with 100 µL HCl (12M) at 120°C for 3 hours. Then 25µL of each sample were used in duplicates for hydroxyproline quantification.

Sequence of PRDX2 C51S and WT resistant to sg*PRDX2*

The yellow sequence corresponds to sg*PRDX2* target sequence, and the bold letters correspond to the silent mutation for guide resistance. TGC codon (cysteine) was mutated in AGC (serine) in the C51S sequence (indicated by a star).

WT resistant to sg*PRDX2*:
/5PHOS/AAAAACTAGTGCCGCC**ATG**GCCTCCGGTAACGCGCGCATCGGAAAGCCAGCCCC
TGACTTCAAGGCCACAGCGGTGGTTGATGGCGCCTTCAAAGAGGT**A**AA**A**CT**A**TC**A**GA**T**TA
TAAAGGGAAGTACGTGGTCCTCTTTTTCTACCCTCTGGACTTCACTTTTGTG**TGC***CCCAC
CGAGATCATCGCGTTCAGCAACCGTGCAGAGGACTTCCGCAAGCTGGGCTGTGAAGTGCT
GGGCGTCTCGGTGGACTCTCAGTTCACCCACCTGGCTTGGATCAACACCCCCCGGAAAGA
GGGAGGCTTGGGCCCCCTGAACATCCCCCTGCTTGCTGACGTGACCAGACGCTTGTCTGA
GGATTACGGCGTGCTGAAAACAGATGAGGGCATTGCCTACAGGGGCCTCTTTATCATCGA
TGGCAAGGGTGTCCTTCGCCAGATCACTGTTAATGATTTGCCTGTGGGACGCTCCGTGGA
TGAGGCTCTGCGGCTGGTCCAGGCCTTCCAGTACACAGACGAGCATGGGGAAGTTTGTCC
CGCTGGCTGGAAGCCTGGCAGTGACACGATTAAGCCCAACGTGGATGACAGCAAGGAAT
ATTTCTCCAAACACAATTAGTAAGTTT

C51S resistant to sg*PRDX2*:
/5PHOS/AAAAACTAGTGCCGCC**ATG**GCCTCCGGTAACGCGCGCATCGGAAAGCCAGCCCC
TGACTTCAAGGCCACAGCGGTGGTTGATGGCGCCTTCAAAGAGGT**A**AA**A**CT**A**TC**A**GA**T**TA
TAAAGGGAAGTACGTGGTCCTCTTTTTCTACCCTCTGGACTTCACTTTTGTG**AGC***CCCAC
CGAGATCATCGCGTTCAGCAACCGTGCAGAGGACTTCCGCAAGCTGGGCTGTGAAGTGCT
GGGCGTCTCGGTGGACTCTCAGTTCACCCACCTGGCTTGGATCAACACCCCCCGGAAAGA
GGGAGGCTTGGGCCCCCTGAACATCCCCCTGCTTGCTGACGTGACCAGACGCTTGTCTGA
GGATTACGGCGTGCTGAAAACAGATGAGGGCATTGCCTACAGGGGCCTCTTTATCATCGA
TGGCAAGGGTGTCCTTCGCCAGATCACTGTTAATGATTTGCCTGTGGGACGCTCCGTGGA
TGAGGCTCTGCGGCTGGTCCAGGCCTTCCAGTACACAGACGAGCATGGGGAAGTTTGTCC
CGCTGGCTGGAAGCCTGGCAGTGACACGATTAAGCCCAACGTGGATGACAGCAAGGAAT
ATTTCTCCAAACACAATTAGTAAGTTT

SUPPLEMENTARY FIGURES:

Supplementary Figure 1

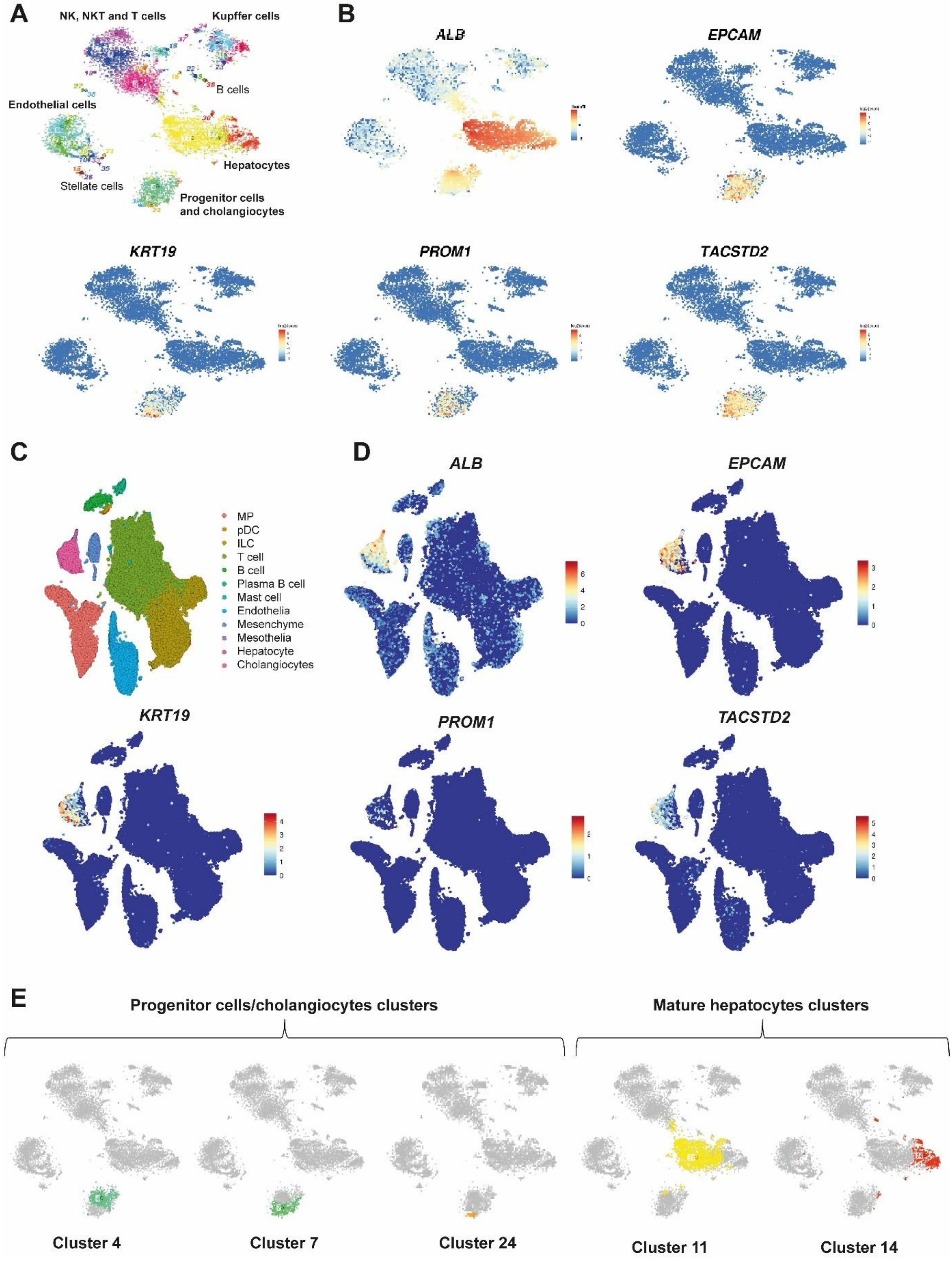

Supplementary Figure 1 (related to Figure 1C-E): Single cell RNA-Seq analysis of normal and cirrhotic patient livers. **A**. *t*-SNE map of single-cell transcriptomes from normal patient liver tissues (nine donors) highlighting the main liver cell compartments. Cells sharing similar transcriptome profiles are grouped by clusters and each dot represents one cell. **B.** Expression *t*-SNE maps of *ALB* (hepatocytes)*, ECPAM, KRT19, PROM1* and *TACSTD2* (progenitor cells and cholangiocytes) are shown. The color bar indicates log2 normalized expression. Data extracted from (6). **C**. Cell lineages from normal and cirrhotic patient liver tissues (5 non-diseased and 5 cirrhotic human livers) inferred from expression of marker gene signatures. MP: mononuclear phagocyte; pDC: plasmacytoid dendritic cell; ILC: innate lymphoid cell. **D.** Expression *t*-SNE maps of *ALB* (hepatocytes)*, ECPAM, KRT19, PROM1* and *TACSTD2* (progenitor cells and cholangiocytes) are shown. The color bar indicates log2 normalized expression. Data extracted from (7). **E**. *t*-SNE map showing the different cell clusters presented in Figure 1E corresponding to progenitor cells/cholangiocytes and mature hepatocytes clusters. Data extracted from (6).

Supplementary Figure 2

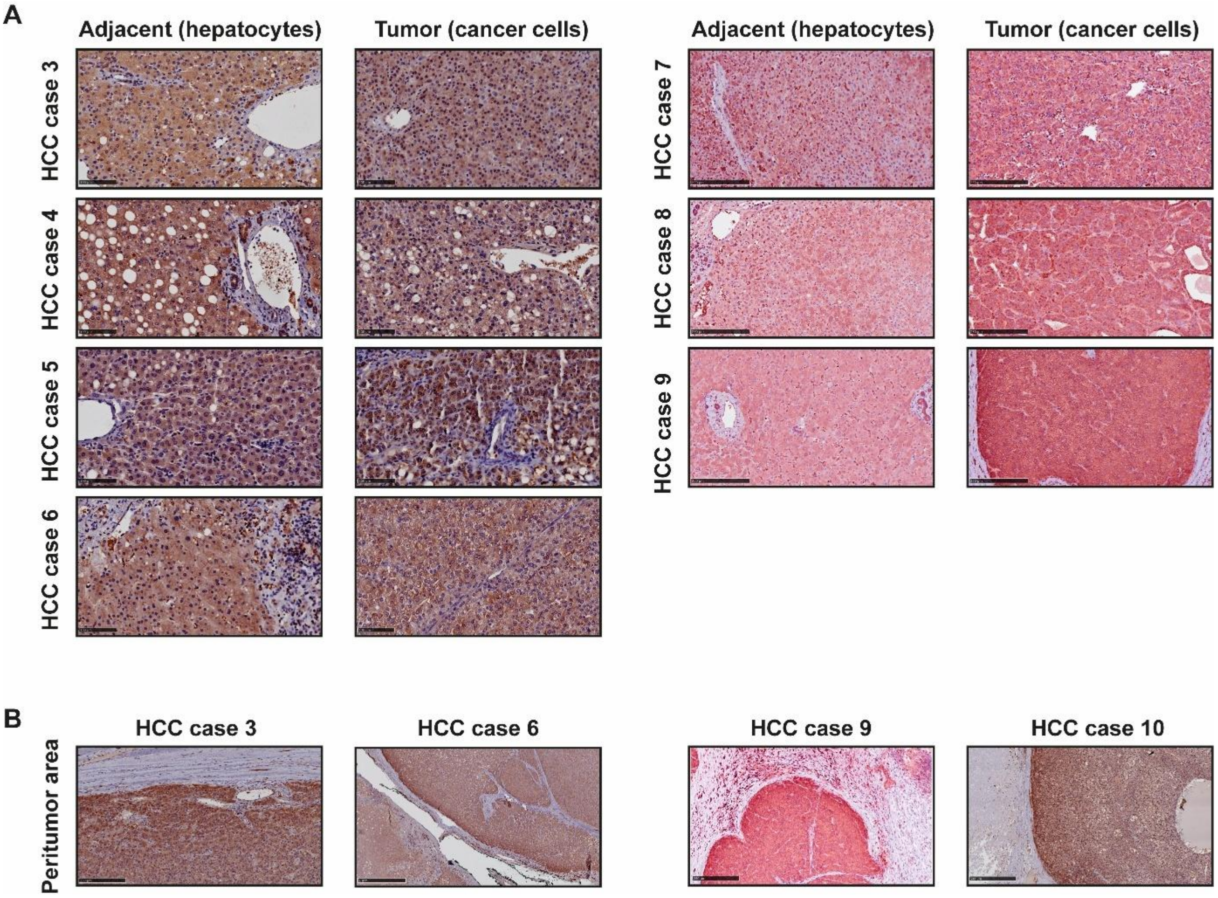

Supplementary Figure 2 (related to Figure 1F): PRDX2 expression in HCC patient tissues. **A**. Representative pictures of IHC analyses showing PRDX2 protein expression in adjacent non tumoral tissues and tumor tissues from HCC patients. Clinical information are available in **Supplementary Table 1**. Left panels: CRB Strasbourg tissue collection, scale bar 100 µm. Right panels: LivMod Strasbourg tissue collection, scale bar 250 µm. **B.** Representative images of the peritumor areas showing a gradient in PRDX2 expression (when peritumor area is well defined). The difference in color between the 2 cohorts are due to different scanner settings.

Supplementary Figure 3

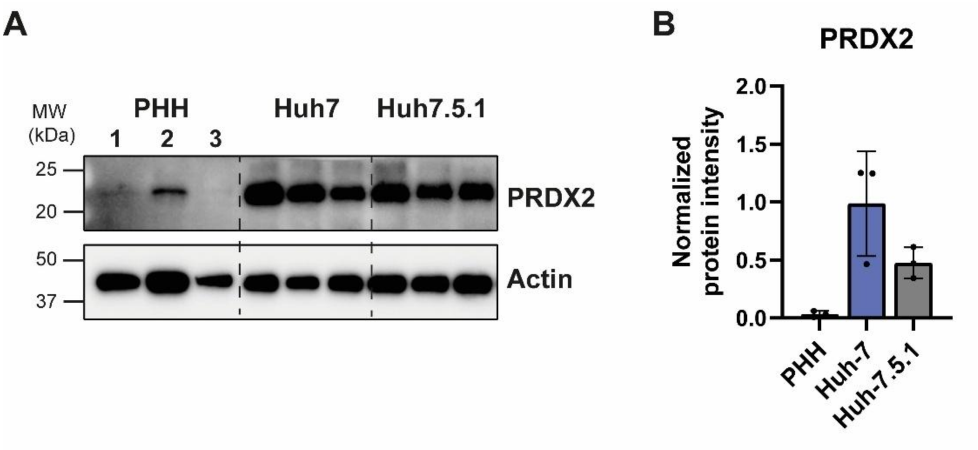

Supplementary Figure 3 (related to Figure 1): PRDX2 is overexpressed in HCC derived cell lines (Huh7) compared to primary human hepatocytes (PHH). A. PRDX2 expression was assessed in PHH (3 donors), Huh-7 and Huh-7.5.1 cells by Western blot analysis. **B.** The graph shows means +/- sd of normalized protein intensity in the different cell types.

Supplementary Figure 4

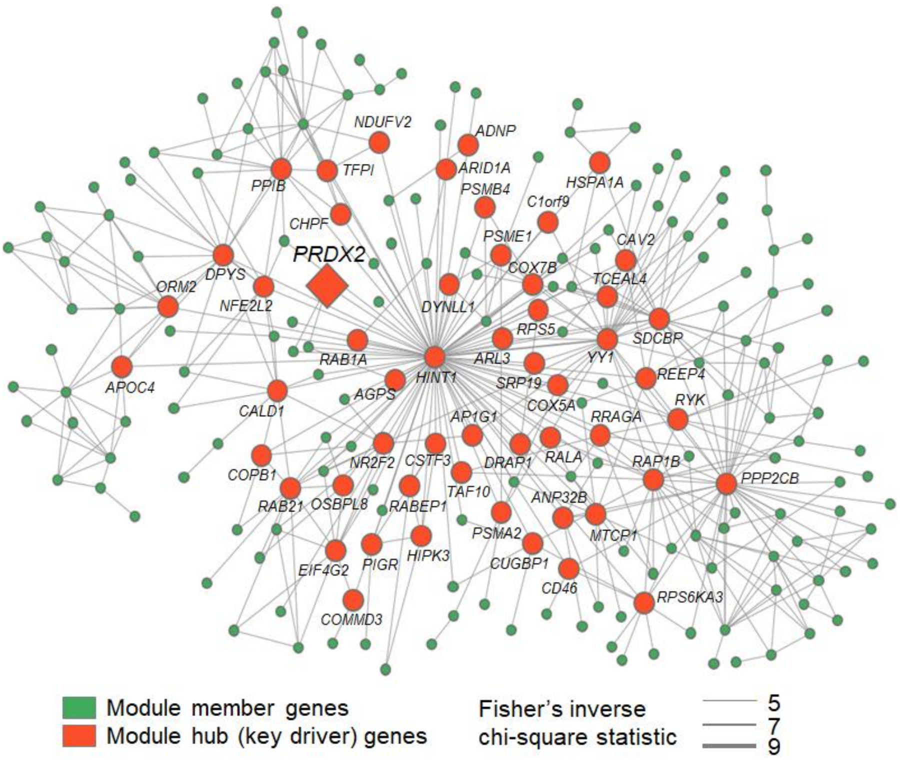

Supplementary Figure 4 (related to Figure 1): Co-regulatory cirrhosis gene module associated with elevated HCC risk in cirrhotic patients. Gene co-regulatory networks are visualized for the module no. 8 identified by the planar filtered network analysis (PFNA) algorithm (8). The module no. 8 is associated with increased HCC risk and HCC recurrence in patients, independently from cirrhosis (8). Each node represents a gene. Width of the edges indicates magnitude of co-regulation (co-expression). Gene network was visualized by using Cytoscape software (ver. 3.9.1) (9).

Supplementary Figure 5

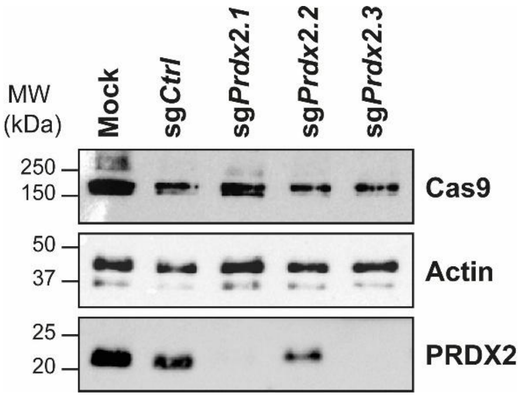

Supplementary Figure 5 (related to Figure 2A). Selection of single guide RNA (sgRNA) targeting mouse *Prdx2* in Hepa 1-6 Cas9 cells. *Prdx2* KO was performed in Hepa 1-6 Cas9 cells using Lentivirus coding for *Prdx2*-specific sgRNAs or sg*Ctrl* targeting GFP. *Prdx2* KO was assessed by Western-blot analysis. sg*Prdx2.3* was selected for *in vivo* experiments.

Supplementary Figure 6

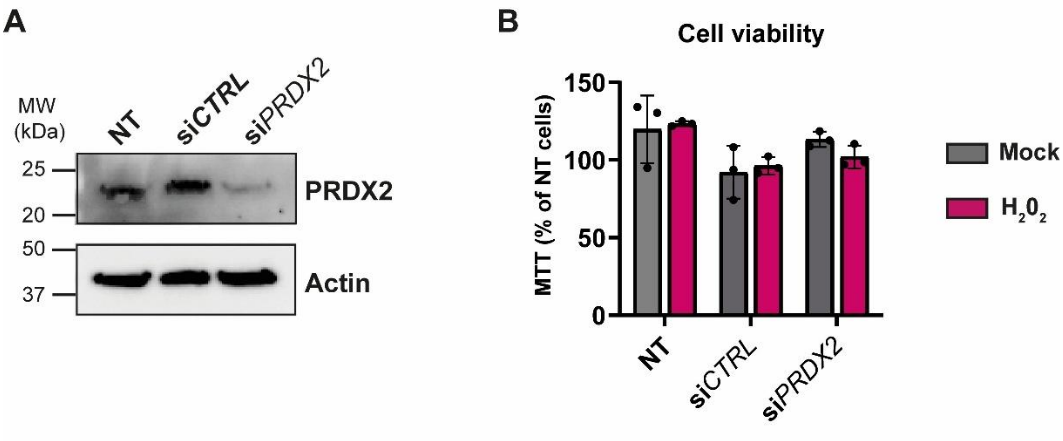

Supplementary Figure 6 (related to Figure 2). ***PRDX2*** **knock-down (KD) in primary human hepatocytes (PHH) showed no cytotoxic effect. A.** Western blot analysis showing *PRDX2* KD in PHH. NT = non-transfected. **B.** MTT assay was performed to measure cell viability in non-treated (mock) and H_2O_2 treated PHH to induce oxidative stress (300 µM, 6 hours). The graph shows means +/- sd of cell viability in % to non-transfected cells of one experiment performed in triplicate.

Supplementary Figure 7

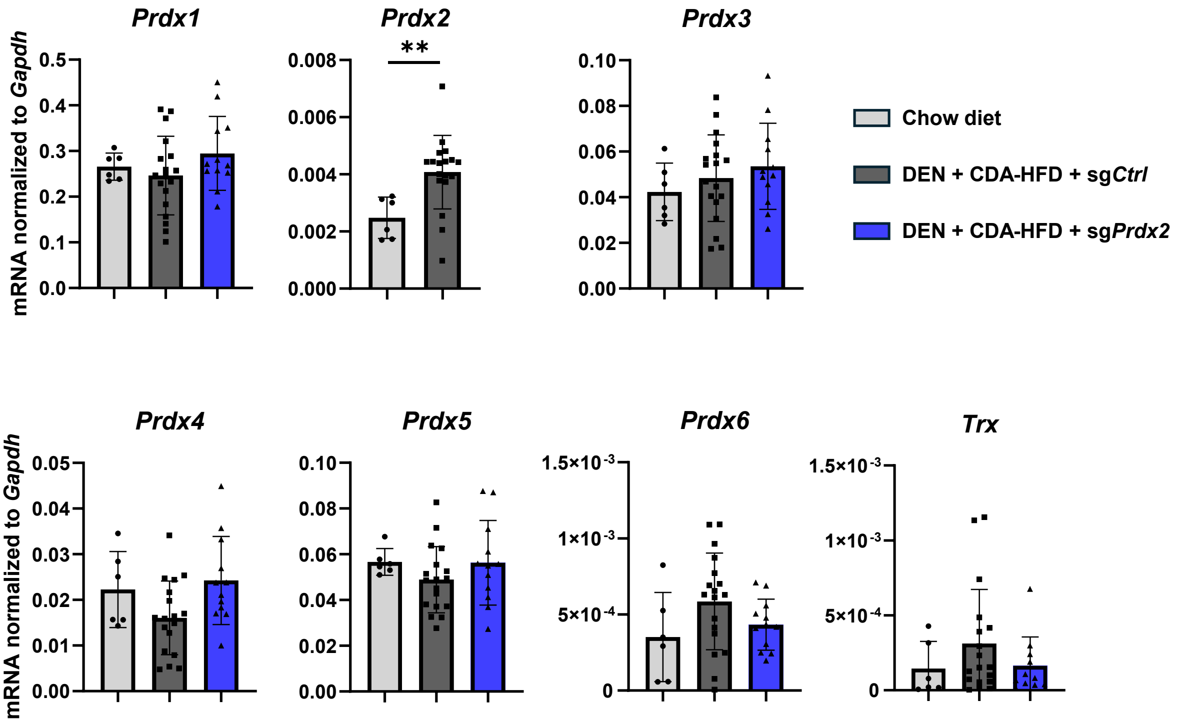

Supplementary Figure 7 (related to Figure 2B). *Prdx2* KO does not significantly affect expression of the other *Prdx* family members in mouse livers. *Prdx* 1 to 6 and thioredoxin (*Txn*) expression was measured by qRT-PCRs in mouse livers. The graph shows means +/- sd of *Prdx* mRNAs normalized to *Gapdh* (Chow, n = 6; sg*Ctrl* n = 18; sg*Prdx2,* n = 16). ** $p < 0.005$ (Mann-Whitney test for *Prdx2*; Kruskal-Wallis test for other *Prdx*).

Supplemental Figure 8

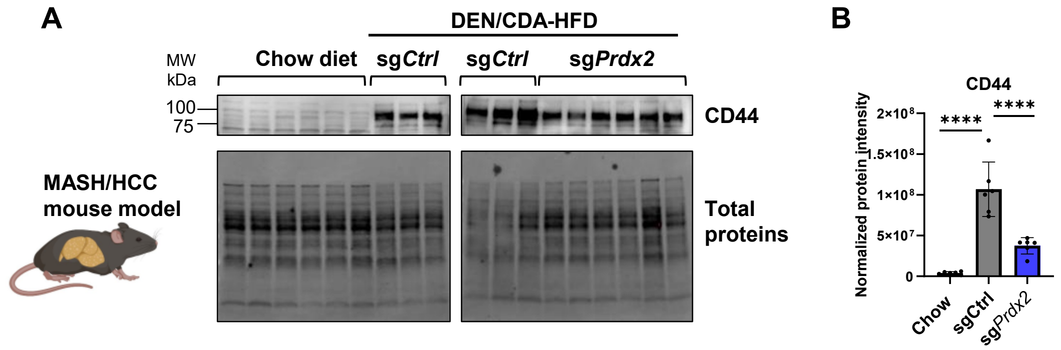

Supplementary Figure 8 (related to Figure 3B): *Prdx2* KO decreases CD44 expression in a MASH/HCC mouse model. A. CD44 expression was assessed in mouse liver tissues by Western blot analysis (6 animals per group). **B.** The graph shows means +/- sd of protein intensity normalized to total proteins (stain free technology). **** $p < 0.0001$ (One way ANOVA followed by Tukey's multiple comparisons test).

Supplemental Figure 9

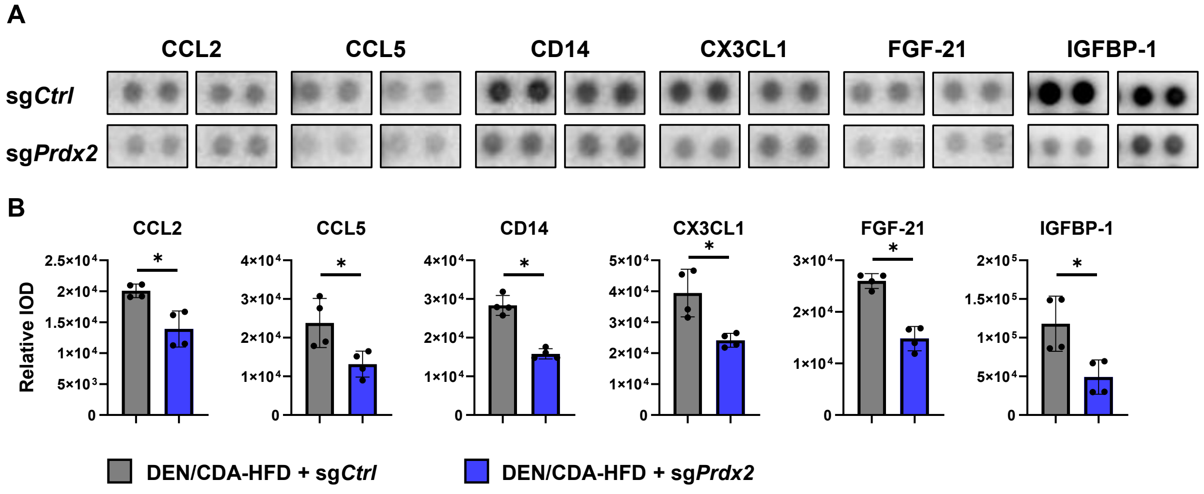

Supplementary Figure 9 (related to Figure 3C): *Prdx2* KO decreases pro-inflammatory cytokine secretion in a MASH/HCC mouse model. A. Cytokine array analysis of mouse sera (2 animals per group, in duplicate). The dot plots show expression of secreted cytokines significantly decreased by *Prdx2* KO. B. The graphs show means +/- sd of relative integrated optical density of the proteins (IOD). * $p < 0.05$ (Mann-Whitney test). CCL2 = C-C motif chemokine ligand 2; CCL5 = C-C motif chemokine ligand 5; CD14 = monocyte differentiation antigen CD14; CX3CL1 = C-X3-C Motif Chemokine Ligand 1; FGF-21 = fibroblast growth factor 21; IGFBP-1 = insulin like growth factor binding protein 1.

Supplementary Figure 10

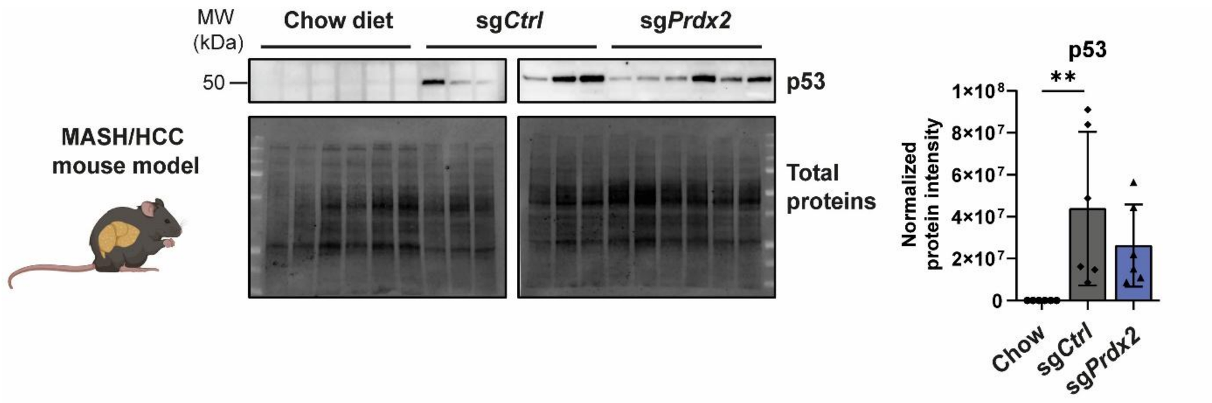

Supplementary Figure 10 (related to Figure 3D). Effect of *Prdx2* KO on p53 expression. P53 expression was assessed by Western blot analysis in mouse livers (MASH/HCC model, 6 animals per group)**.** The graph shows means +/- sd of protein intensity normalized to total proteins. ** $p < 0.005$ (Kruskal-Wallis test followed by Dunn's multiple comparisons test).

Supplementary Figure 11

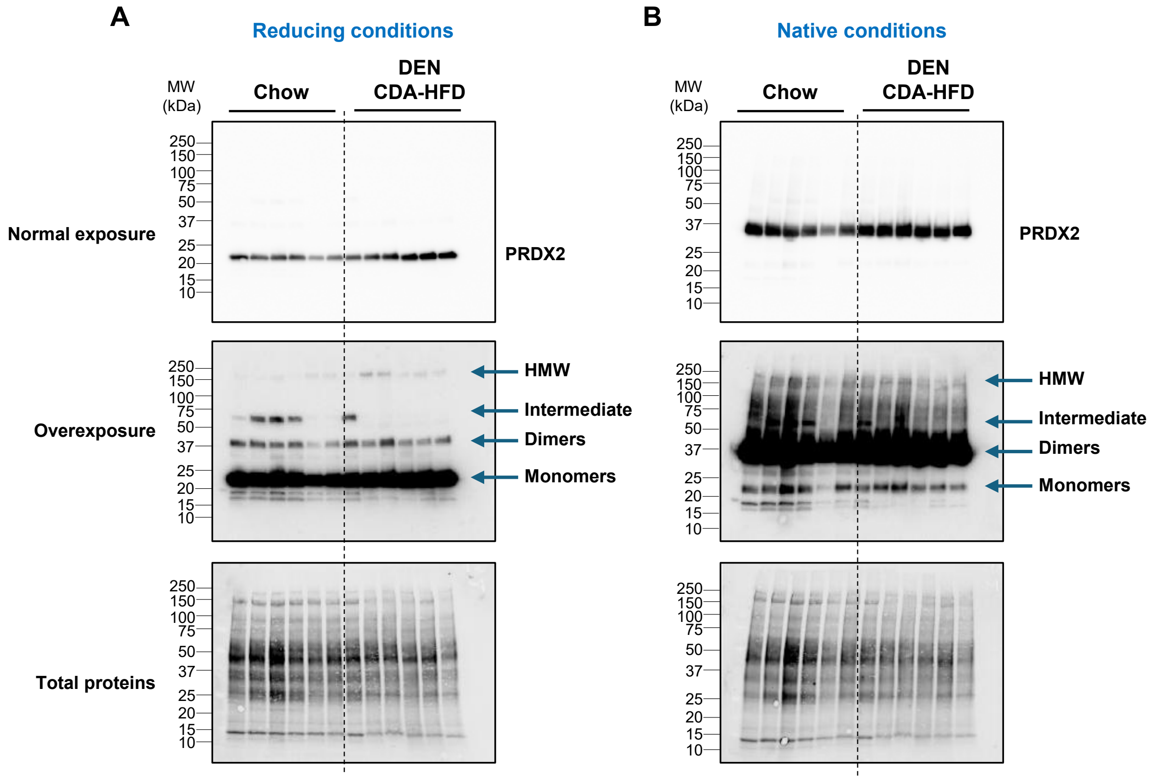

Supplementary Figure 11 (related to Figure 3): PRDX2 expression and conformation in mouse livers. PRDX2 expression and conformation were analyzed in mouse livers (Chow diet and DEN/CDA-HFD, 6 animals per group) by Western blot analysis (A) in reducing conditions and (B) non-denaturing polyacrylamide gel electrophoresis (native conditions). Arrows show the different PRDX2 conformation: monomers, dimers, intermediate forms and high molecular weight forms (HMW). The full-length Western blots are shown in this figure.

Supplementary Figure 12

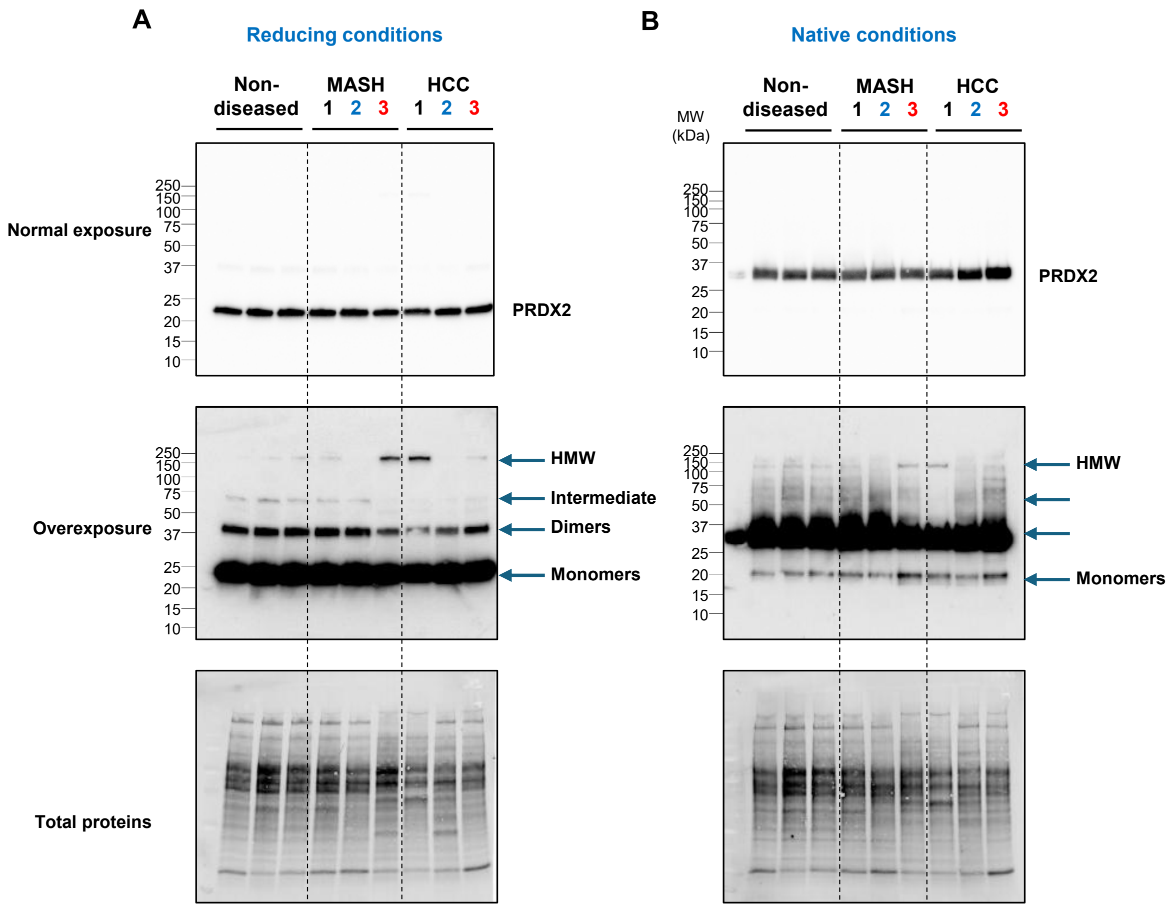

Supplementary Figure 12 (related to Figure 3): PRDX2 expression and conformation in human livers. PRDX2 expression and conformation were analyzed in patient livers (non-diseased, MASH and HCC as paired samples, 3 patients per group) by Western blot analysis (A) in reducing conditions and (B) non-denaturing polyacrylamide gel electrophoresis (native conditions). Arrows show the different PRDX2 conformation: monomers, dimers, intermediate forms and high molecular weight forms (HMW). The full-length Western blots are shown in this figure.

Supplementary Figure 13

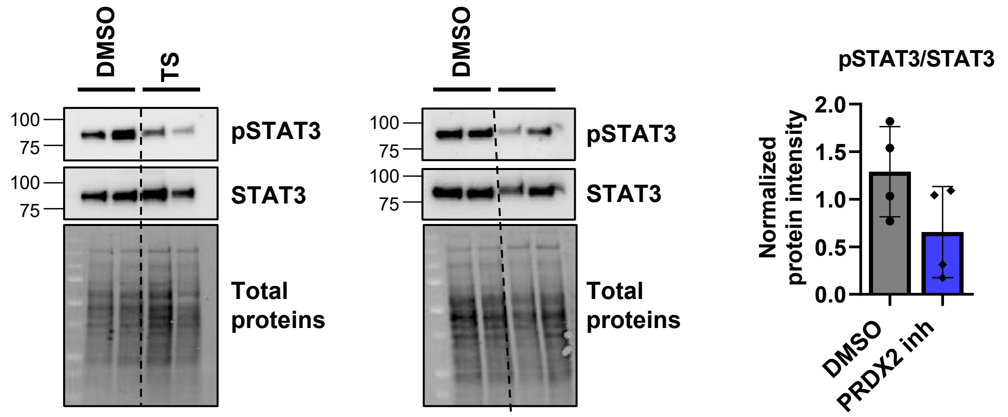

Supplementary Figure 13 (related to Figure 4C). Inhibition of PRDX2 decreases STAT3 activation in PHH. PHH isolated from 2 different donors were treated with thiostrepton or DMSO (control). The effect on STAT3 activation was assessed by Western blot analysis. The graph shows means +/- sd of protein intensity normalized to total proteins and expressed as a ratio (pSTAT3/STAT3). n = 4, Mann-Whitney test ns.

Supplementary Figure 14

A

Ctrl

*Sequence**	5'-UGG UUU ACA UGU CGA CUA ATT -3'	
3' Overhang	*sense (5'-3')*	dTdT
	antisense (5'-3')	dTdT

Prdx2

*Sequence**	5'-AAA UCA AGC UUU CGG ACU ATT -3'	
3' Overhang	*sense (5'-3')*	dTdT
	antisense (5'-3')	dTdT

B

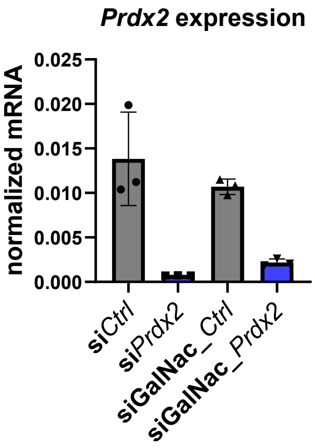

Supplementary Figure 14 (related to Figure 5A): Validation of GalNac siRNA targeting *Prdx2* efficacy. A. Sequence and design of GalNac siRNAs targeting *Prdx2* expression and non-targeting *Ctrl*. **B.** *Prdx2* expression was measured by qRT-PCR in Hepa1.6 cells after transfection with regular siRNAs or GalNac siRNAs . The graph shows means +/- sd of mRNAs normalized to *Gapdh* of one representative experiment (out of 2) performed in triplicate (n = 3).

Supplementary Figure 15

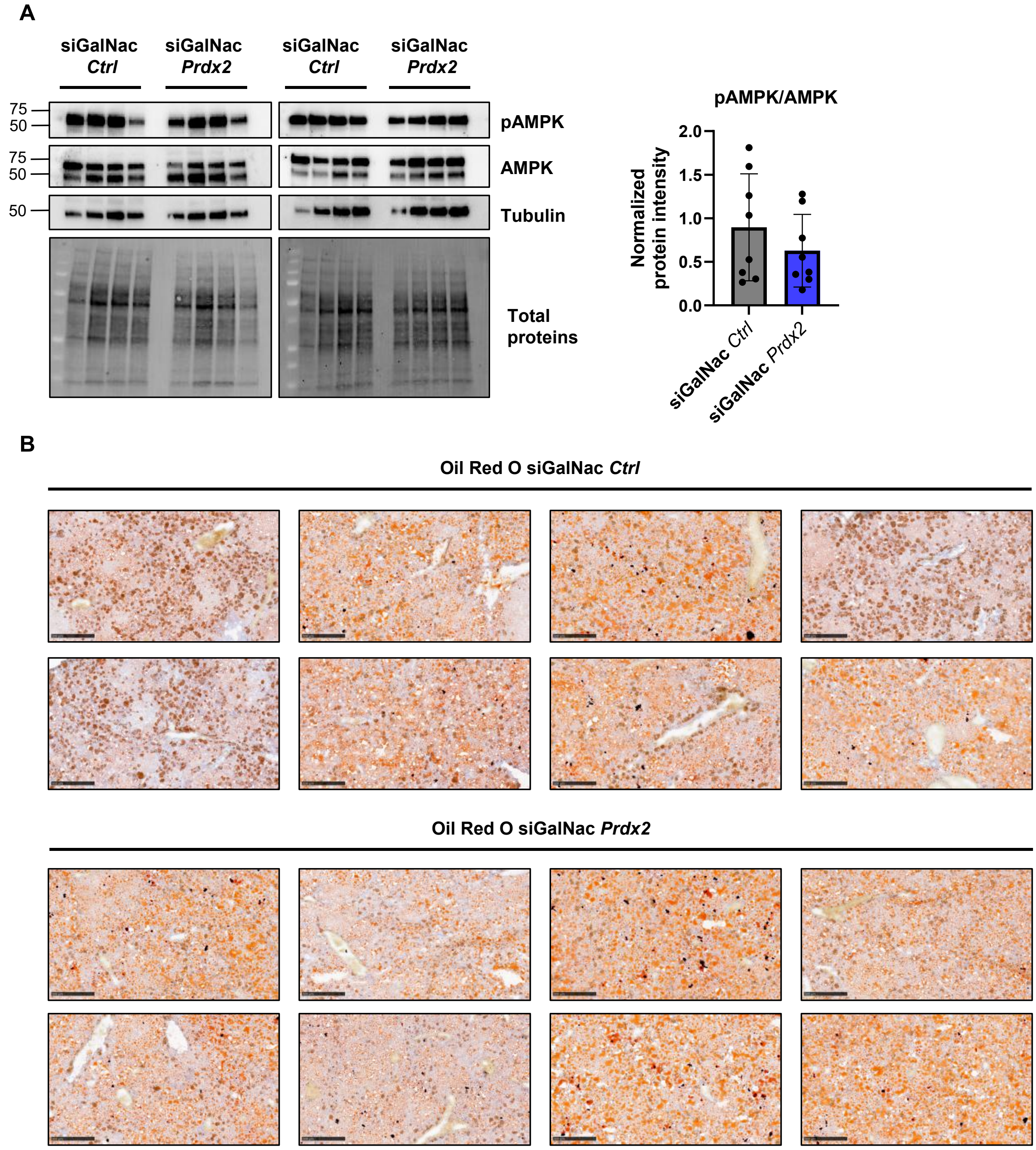

Supplementary Figure 15 (related to Figure 5B-C): AMPK expression, activation and lipid accumulation in mouse livers treated with GalNac siRNAs. A. AMPK expression and activation was assessed in liver tissues by Western blot analysis (8 animals per group). The graph shows means +/- sd of protein intensity normalized to total proteins (stain free technology). **B.** Representative images of Oil Red O stainings performed on animal livers. One picture corresponds to one animal. Scale bar = 500 µm.

Supplementary Figure 16

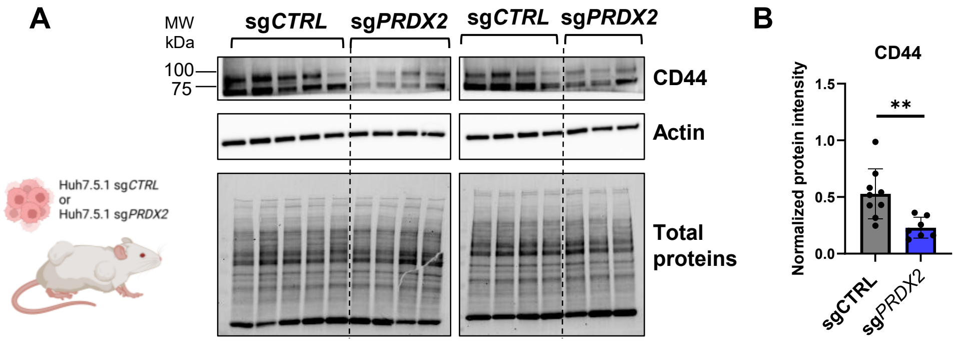

Supplementary Figure 16 (related to Figure 6E): *PRDX2* KO decreases CD44 expression in a CDX mouse model. A. CD44 expression was assessed in tumors by Western blot analysis (sg*CTRL*, n = 9; sg*PRDX2*, n = 7). **B.** The graph shows means +/- sd of protein intensity normalized to total proteins (stain free technology). ** $p < 0.001$ (Mann-Whitney test).

Supplementary Figure 17

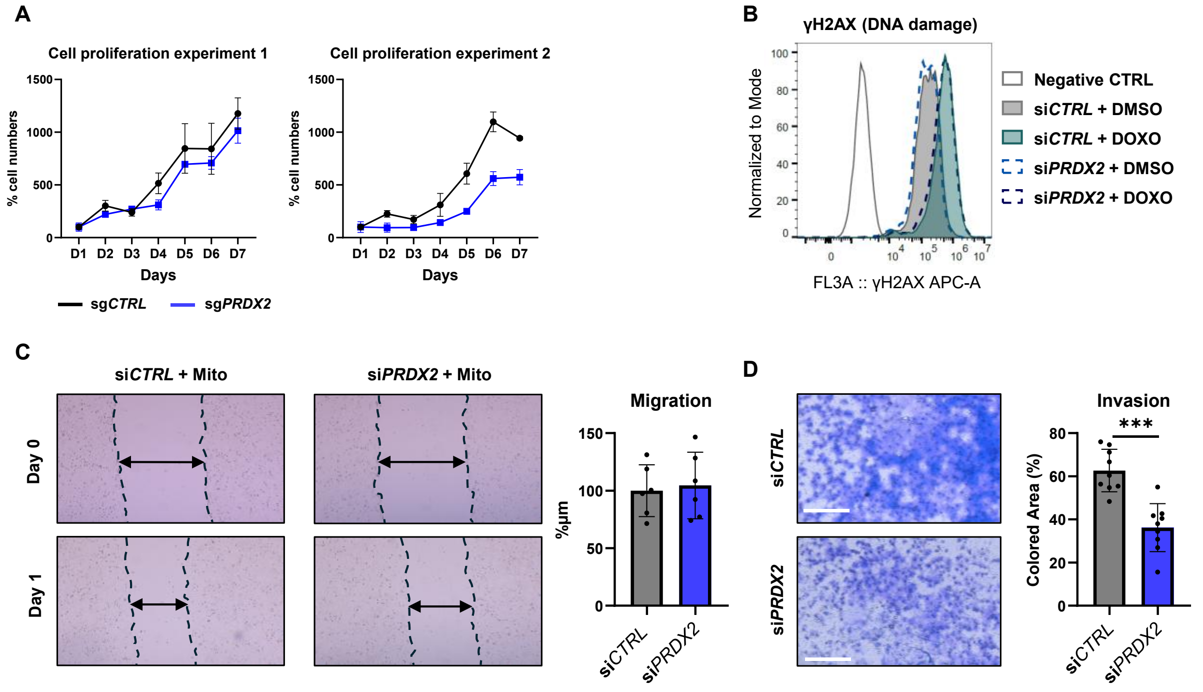

Supplementary Figure 17 (related to Figure 8A-B): Role of PRDX2 in cancer development and progression. A. *PRDX2* KO decreases Huh7 cancer cell growth. The graphs show means +/- sd of cell numbers (in percentage to input) over a time course of 7 days. Two representative experiments performed in triplicate (out of 4) are shown. **B.** *PRDX2* KD has no impact on DNA damage in Huh7 cells. DNA damages were measured using the histone H2AX phosphorylated (γH2AX) marked detected by flow cytometry after stimulation of Huh7 cells with doxorubicin (DOXO) to induce oxidative stress and cell senescence, or DMSO control. One representative histogram out of 3 is shown. **C.** *PRDX2* KD has no impact on Huh7 cancer cell migration. Cell migration was assessed by a wound healing assay in presence of mitomycin (mito) to block cell proliferation. Representative pictures are shown. The graphs show means +/- sd of cell migration from three independent experiments performed in duplicate (n = 6). **D.** *PRDX2* KD decreases Huh7 cancer cell invasion. Cell invasion was assessed by a transwell assay. Representative pictures are shown. The graphs show means +/- sd of cell invasion from three independent experiments performed in triplicate (n = 9). *** $p < 0.0001$ (Mann-Whitney test).

Supplementary Figure 18

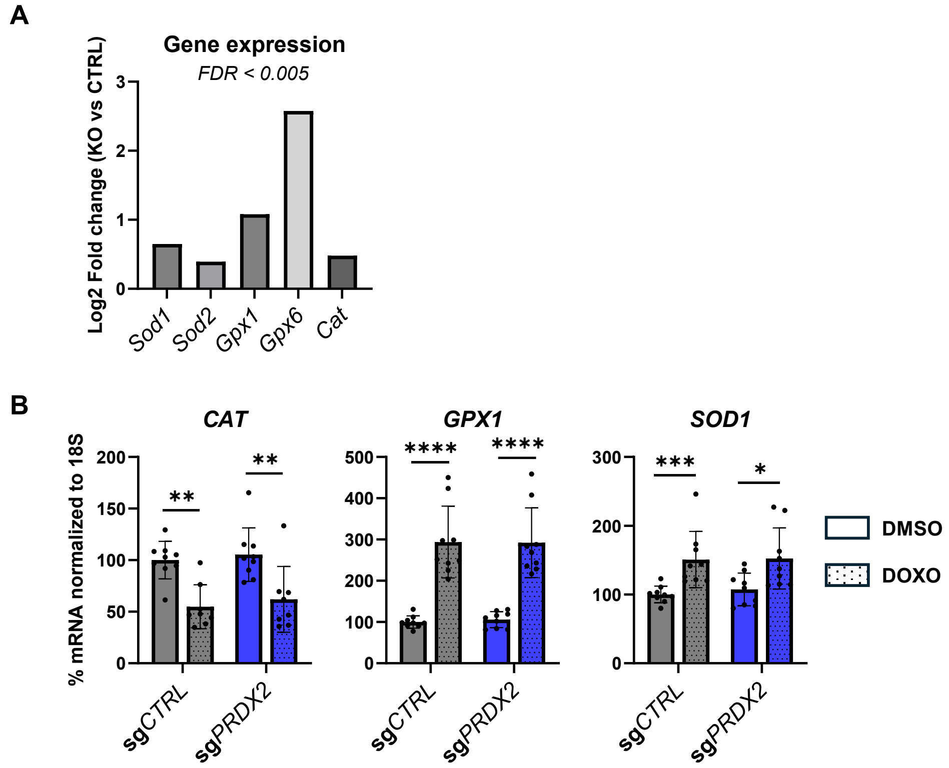

Supplementary Figure 18 (related to Figures 8E-F): Expression of the main antioxidant defense systems in mouse livers and in *PRDX2* KO cancer cells. A. *Prdx2* KO in hepatocytes increases expression of other antioxidant enzymes, catalase (*Cat*), glutathione peroxidase (*Gpx*) and super oxide dismutase (*Sod*) (RNA-Seq data, expressed in log2 fold change). False discovery rates (FDR) < 0.005. **B.** *PRDX2* KO in cancer cells does not impact expression of antioxidant enzymes, even under oxidative stress induced by doxorubicin (DOXO). Gene expression was measured by qRT-PCRs in *PRDX2* KO and *CTRL* Huh7.5.1 cells. The graph shows means +/- sd of mRNAs normalized to *18S* of three independent experiments performed in triplicate (n =9).

SUPPLEMENTARY TABLES:

Supplementary Table 1 (related to supplementary Figure 1): Clinical information of the HCC cases. The column “PRDX2” indicate the level of expression of PRDX2 in the tumor tissue compared to adjacent non tumoral tissue.

Case ID	Number	Stage	Phenotype	Disease	PRDX2
HCC case 1	557 tumor 1	pT2	Well differentiated	No fibrosis, steatosis	+
HCC case 2	557 tumor 2	pT2	Well differentiated	No fibrosis, steatosis	+
HCC case 3	544	pT1b	Well differentiated	Cirrhosis, steatosis	-
HCC case 4	559	pT1	Moderately to well	Cirrhosis, steatosis	-
HCC case 5	576	pT1	Well differentiated	Fibrosis, steatosis	+
HCC case 6	577	pT3	Well differentiated	Cirrhosis, steatosis	=
HCC case 7	558	pT1b	Well differentiated	Fibrosis, steatosis	+
HCC case 8	608	pT1b	Moderately differentiated	Cirrhosis	+
HCC case 9	615	pT3	Moderately differentiated	No fibrosis	+
HCC case 10	551	pT4	Well differentiated	Vascular invasion, micronodules	NA

Supplementary Table 2 (related to Figure 1H): The 32 gene PLS. List of poor-and good-prognosis associated genes and of housekeeping genes used for normalization (8).

**Poor-prognosis genes**		
**Gene ID**	**Gene Symbol**	**Description**
3983	ABLIM1	Actin binding LIM protein 1
1293	COL6A3	Collagen, type VI, alphae 3
9170	EDG4	Endothelial differentiation lysophosphatidic acid G-protein-coupled
1950	EGF	Epidermal growth factor (beta-urogast)
2043	EPHA4	EPH receptor A4
2326	FMO1	Flavin containing monooxygenase 1
2488	FSHB	Follicle stimulating hormone beta polypeptide
2877	GPX2	Glutathione peroxidase 2 (gastrointestinal)
3680	ITGA9	Integrin , alpha 9
4316	MMP7	Matrix metallopeptidase 7 (matrilysin, uterine)
23397	NCAPH	Non-SMC condensin I complex, subunit H
4843	NOS2A	Nitric oxide synthase 2A (inducible, hepatocytes)
4922	NTS	Neurotensin
5593	PRKG2	Protein kinase, cGMP-dependent type II
23029	RBM34	RNA binding motif protein 34
5055	SERPINB2	Serpin peptidase inhibitor, clade B (ovalbumin), member 2
6456	SH3GL2	SH3-domain GFB2-like 2
6672	SP100	SP100 nuclear antigen
7204	TRIO	Triple functional domain (PTPRF interacting)
**Good-prognosis genes**		
6296	ACSM3	Acyl-CoA synthetase medium-chair family member 3
151	ADRA2B	Adrenergic, alpha-2B- receptor
223	ALDH9A1	Aldehyde dehydrogenase 0 family, member A1
3612	IMPA1	Inositol(myo)-1(or 4)-monophosphatase 1
5207	PFKFB1	6-phosphofructo-2-kinase/fructose-2,6-biphosphatase 1
5313	PKLR	Pyruvate kinase, liver and RBC
5502	PPP1R1A	Protein phosphatse 1, regulatory (inhibitor) submunit 1A
5691	PSMB3	Proteasome (promosome, macropain) subunit, beta type, 3
5771	PTPN2	Protein tyrosine phosphatase, non-receptor type 2
6018	RLF	Rearranged L-myc fusion
9252	RPS6KA5	Ribosomal protein S6 kinase, 90 kDa, polypeptide 5
27346	TMEM97	Transmembrane protein 97
7276	TTR	Transthyretin (prealbumin, amyloidosis type I)
**Housekeeping genes**		
506	ATP5B	ATP synthase F1 subunit beta
7917	BAT3	BAG cochaperone 6
1351	COX8A	Cytochrome c oxidase subunit 8A
3094	HINT1	Histidine triad nucleotide binding protein 1
3181	HNRNPA2B1	Heterogeneous nuclear ribonucleoprotein A2/B1
4695	NDUFA2	NADH:ubiquinone oxidoreductase subunit A2

Supplementary Table 3 (related to Figure 3): Gene set enrichment analysis (GSEA) of RNA-Seq from mouse liver tissues (MASH/HCC mouse model). Refer to the excel table "GSEA analysis of RNA-Seq from mouse liver tissues". The pathways are ranked according to the normalized enrichment score (NES) (sg*Prdx2* vs sg*Ctrl*). FDR < 0.05 are considered as significant enriched pathways(10). RNA-Seq data were deposited in the NCBI Gene Expression Omnibus database with the accession number: GSE199320.

Supplementary Table 4: Reagents and resources

**Oligonucleotides used for gene expression assay**			
**Species**	**Gene**	**5'-3' Sequence**	
		**Forward**	**Reverse**
**Mouse**	*Abca1*	5'-AACAGTTTGTGGCCCTTTTG-3'	5'-AGTTCCAGGCTGGGGTACTT-3'
	Gapdh	5'-TTCACCACCATGGAGAAGGC-3'	5'-TAAGCAGTTGGTGGTGCAGG-3'
	Prdx2	5'-GGGCCACGCATAAAAGGTTC-3'	5'-CCATGACTGCGTGAGCAAGA-3'
	Abcg5	Mm00446241_m1 (ThermoFischer)	
	Abcg8	Mm00445980_m1 (ThermoFischer)	
	Acat2	Mm00782408_s1 (ThermoFischer)	
	Col1a1	Mm00801666_g1 (ThermoFischer)	
	Gapdh	4351309 (Applied Biosystems)	
	Scarb1	Mm00450234_m1 (ThermoFischer)	
	Tgfb1	Mm01178820_m1 (ThermoFischer)	
	Timp1	Mm01341361_m1 (ThermoFischer)	
	Prdx1	Mm01621996_s1 (ThermoFischer)	
	Prdx3	Mm00545848_m1 (ThermoFischer)	
	Prdx4	Mm00450261_m1 (ThermoFischer)	
	Prdx5	Mm00465365_m1 (ThermoFischer)	
	Prdx6	Mm07306454_mH (ThermoFischer)	
	Txn1	Mm00726847_s1 (ThermoFischer)	

**Human**	*CAT*	Hs00156308_m1 (ThermoFischer)		
	GPX1	Hs00829989_gH (ThermoFischer)		
	PRDX2	Hs00853603_s1 (ThermoFischer)		
	SOD1	Hs00533490_m1 (ThermoFischer)		
	GAPDH	10555385 (Applied Biosystems)		
	18S	4319413E (Applied Biosystems)		
**Oligonucleotides for mouse *Prdx2* KO**				
**Target**	**5'-3' Sequence**			
CTRL	5’-CACCGGTGAACCGCATCGAGCTGA-3’	5’-AAACTCAGCTCGATGCGGTTCACC-3’		
Prdx2-1	5’-CACCGTCCGATTTGCGCGTTGCCGG-3’	5’-AAACCCGGCAACGCGCAAATCGGAC-3’		
Prdx2-2	5’-CACCGGCCTCCGGCAACGCGCAAAT-3’	5’-AAACATTTGCGCGTTGCCGGAGGCC-3’		
Prdx2-3	5’-CACCGCAACGCGCAAATCGGAAAGT-3’	5’-AAACACTTTCCGATTTGCGCGTTGC-3’		
**sgRNA sequence for *PRDX2* KO**				
**Target**	**5'-3' Sequence**			
CTRL	5’-GGTGAACCGCATCGAGCTGA-3’			
PRDX2-1	5’-GTGAAGCTGTCGGACTACAA-3’			
PRDX2-2	5’-GGCGCCATCAACCACCGCTG-3’			
**siRNA used for KD experiment**				
**Name**		**Source**	**Reference**	
ON-TARGETplus Non-targeting Control Pool		Dharmacon	D-001810-10-20	
ON-TARGETplus Human PRDX2 siRNA – SMART POOL		Dharmacon	L-008178-01-0005	
ON-TARGETplus Mouse Prdx2 siRNA SMART POOL		Dharmacon	L-060550-02-0020	
**GalNac siRNAs**				
**Name**		**Source**	**Reference**	
Ctrl 5’-UGG UUU ACA UGU CGA CUA ATT-3’		MicroSynth	Custom	
Prdx2 5’AAA UCA AGC UUU CGG ACU ATT-3’		MicroSynth	Custom	
**Antibody used for immunohistochemistry**				
**Target**	**Host**	**Reference**	**Dilution**	
PRDX2	Rabbit	10545-2-AP ProteinTech	1:400	

MCM-2	Rabbit	Ab240933 Abcam	1:500
**Antibody used for immunoblotting**			
**Target/clone**	**Host**	**Reference**	**Dilution**
Akt (pan) (C67E7)	Rabbit	4691 Cell Signaling	1:1000
AMPKα (polyclonal)	Rabbit	2532 Cell signaling	1:1000
CRISPR/Cas9 (4G10)	Mouse	C15200216-100 Diagenode	1 :5000
Caspase 3 (polyclonal)	Rabbit	9662 Cell Signaling	1:1000
Cleaved caspase 3 (Asp175)	Rabbit	9661 Cell Signaling	1:1000
Erk (1/2) (216703)	Mouse	MAB1576 R&D systems	1:1000
STAT3 (79D7)	Rabbit	4904 Cell Signaling	1:2000
Phospho Akt (Ser473) (193H12)	Rabbit	4058 Cell Signaling	1:1000
Phospho AMPKα (Thr172) (40H9)	Rabbit	2535 Cell Signaling	1:1000
Phospho Erk1(Thr202/Tyr204) /Erk2(Thr185/Tyr187) (polyclonal)	Rabbit	AF1018 R&D systems	1:2000
Phospho STAT3 (Tyr705) (D3A7)	Rabbit	9145 Cell Signaling	1:2000
PRDX2 (EPR5154)	Rabbit	Abcam ab109367	1:1000
GRP78/BIP (polyclonal)	Rabbit	Abcam ab21685	1:1000
β-actin (AC-15)	Mouse	A5441 Sigma-Aldrich	1:2000
β-tubulin (polyclonal)	Rabbit	GTX101279 GeneTex	1:1000
rabbit IgG conjugated to HRP	Goat	111-035-144 Jackson-immunoresearch	1:10 000
mouse IgG conjugated to HRP	Sheep	NA931 GE Healthcare	1:5000

**Chemicals, Peptides, and Recombinant Proteins**		
**Name**	**Source**	**Reference**
Culturex Basement Membrane Extract	R&D systems	Cat# 3432-005-01
DMSO	Sigma-Aldrich	Cat#41640
H_2O_2	Merck	Cat#1.07209.0250
Clarity WB ECL reagent	Biorad	Cat#170-5061
TRIzol	ThermoFischer Scientific	Cat#15596026
Lipofectamine RNAi Max	Invitrogen	Cat#13778-150

Protease inhibitor EDTA free	Roche	Cat#11873580001
Phosphatase inhibitor cocktail 2	Sigma-Aldrich	Cat#P5726
Phosphatase inhibitor cocktail 3	Sigma-Aldrich	Cat#P0044
Hematoxyline	Bio Optica	Cat#05-M06004
Eosin	Sigma	Cat#HT110116
PicroSirusRed Direct Red 80	Sigma-Aldrich	Cat#365548
OilRed O	Merck	Cat#1.05230.0025
DEN	Sigma-Aldrich	Cat#N0756
iScript™ RT-qPCR Sample Preparation Reagent	Biorad	Cat#170-5061
Hoechst	Invitrogen	Cat#C10337G
Crystal violet	Sigma	Cat# C0775
**Critical Commercial Assays**		
**Name**	**Source**	**Reference**
Corning Costar Transwell cell culture inserts	Corning	Cat#CLS3464-48EA
CellEvent® Caspase-3/7 Green Detection Reagent	Invitrogen	Cat#C10423
TUNEL Assay kit HRP-DAB	Abcam	Cat#ab206386
Cell titer Glo	Promega	
OptiMEM	Gibco	Cat#31985-062
Vectastain Mouse	Vector	Cat#PK-6102
Vectastain Rabbit	Vector	Cat#PK-6101
Click-iT EdU Flow Cytometry Assay Kit	Invitrogen	Cat#C10425
Hydroxyproline Assay kit	Sigma Aldrich	Cat#MAK-0008
FxCycle™ Far Red Stain	Invitrogen™	Cat# F10348
HCS LipidTOX™ Deep Red Neutral Lipid Stain	Invitrogen™	Cat# H34477
DCFDA / H2DCFDA - Cellular ROS Assay Kit	Abcam	Cat# ab113851

SUPPLEMENTARY REREFENCES:

1. Crouchet E, et al. A human liver cell-based system modeling a clinical prognostic liver signature for therapeutic discovery. *Nat Commun*. 2021;12(1):5525.

2. Pietschmann T, et al. Construction and characterization of infectious intragenotypic and intergenotypic hepatitis C virus chimeras. *Proc Natl Acad Sci U S A*. 2006;103(19):7408–7413.

3. Hoshida Y. Nearest Template Prediction: A Single-Sample-Based Flexible Class Prediction with Confidence Assessment. *PLOS ONE*. 2010;5(11):e15543.

4. Reich M, et al. GenePattern 2.0. *Nat Genet*. 2006;38(5):500–501.

5. Juehling F, et al. Targeting clinical epigenetic reprogramming for chemoprevention of metabolic and viral hepatocellular carcinoma. *Gut*. 2021;70(1):157–169.

6. Aizarani N, et al. A human liver cell atlas reveals heterogeneity and epithelial progenitors. *Nature*. 2019;572(7768):199–204.

7. Ramachandran P, et al. Resolving the fibrotic niche of human liver cirrhosis at single cell level. *Nature*. 2019;575(7783):512–518.

8. Nakagawa S, et al. Molecular Liver Cancer Prevention in Cirrhosis by Organ Transcriptome Analysis and Lysophosphatidic Acid Pathway Inhibition. *Cancer Cell*. 2016;30(6):879–890.

9. Shannon P, et al. Cytoscape: A Software Environment for Integrated Models of Biomolecular Interaction Networks. *Genome Res*. 2003;13(11):2498–2504.

10. Subramanian A, et al. Gene set enrichment analysis: a knowledge-based approach for interpreting genome-wide expression profiles. *Proc Natl Acad Sci U S A*. 2005;102(43):15545–15550.
