# Supplementary material for: Targeting peroxiredoxin 2 prevents hepatocarcinogenesis in metabolic liver disease models
Source: J Clin Invest. 2025 Sep 11;135(21):e169395. doi: 10.1172/JCI169395 (PMC12578407; doi:10.1172/JCI169395)

Figure 1G

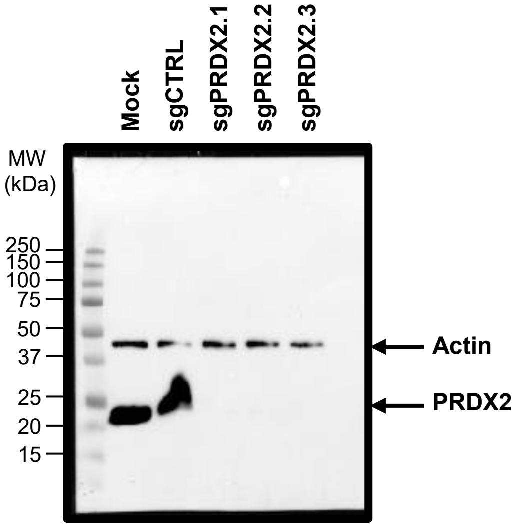
Mock
sgCTRL
sgPRDX2.1
sgPRDX2.2
sgPRDX2.3
MW (kDa)
250
150
100
75
50
37
25
20
15
Actin
PRDX2

Figure 3D (1/4)

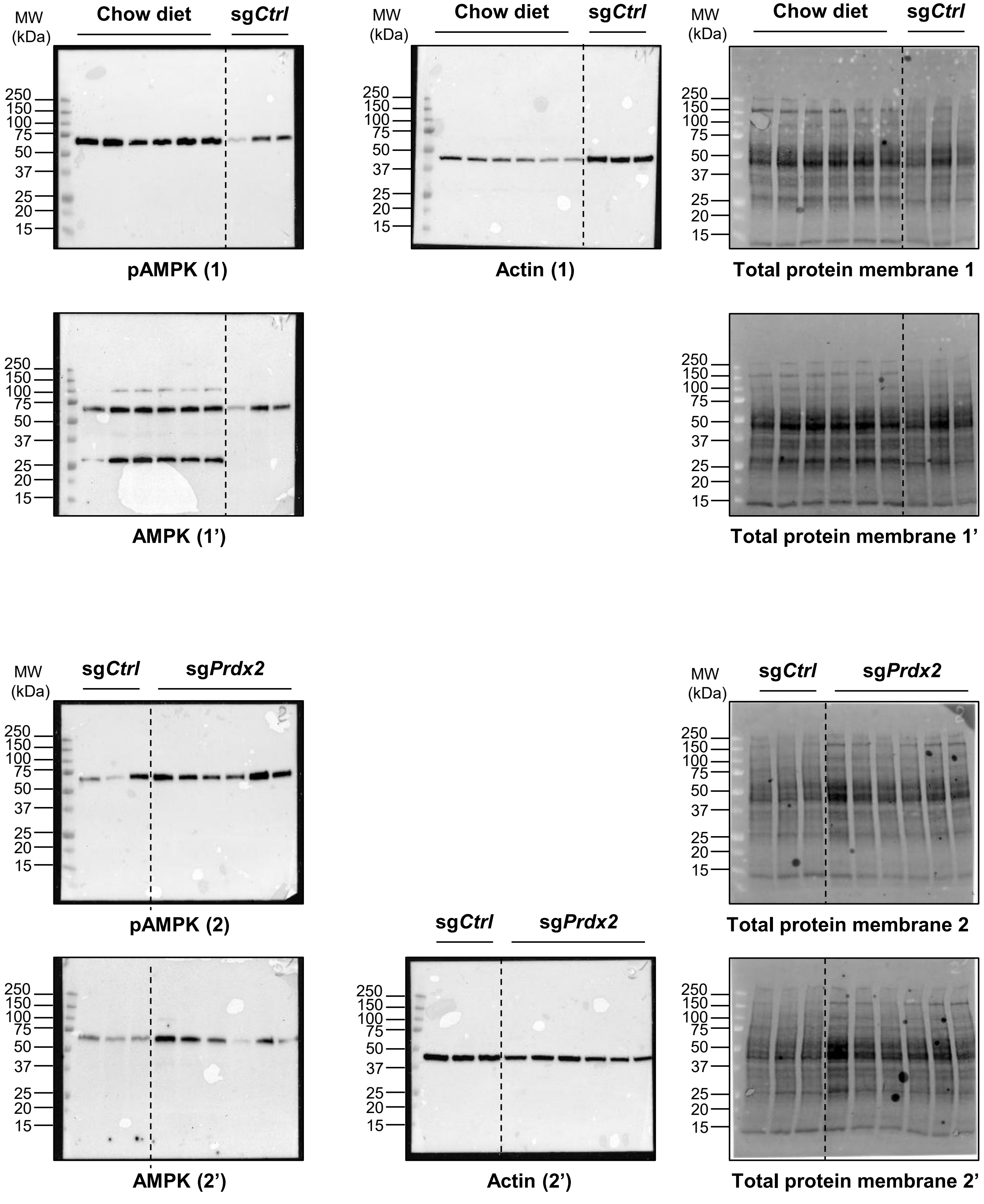

MW (kDa)
Chow diet
sgCtrl
250
150
100
75
50
37
25
20
15
pAMPK (1)
Actin (1)
Total protein membrane 1
AMPK (1')
Total protein membrane 1'
sgPrdx2
pAMPK (2)
Total protein membrane 2
AMPK (2')
Actin (2')
Total protein membrane 2'

Figure 3D (2/4)

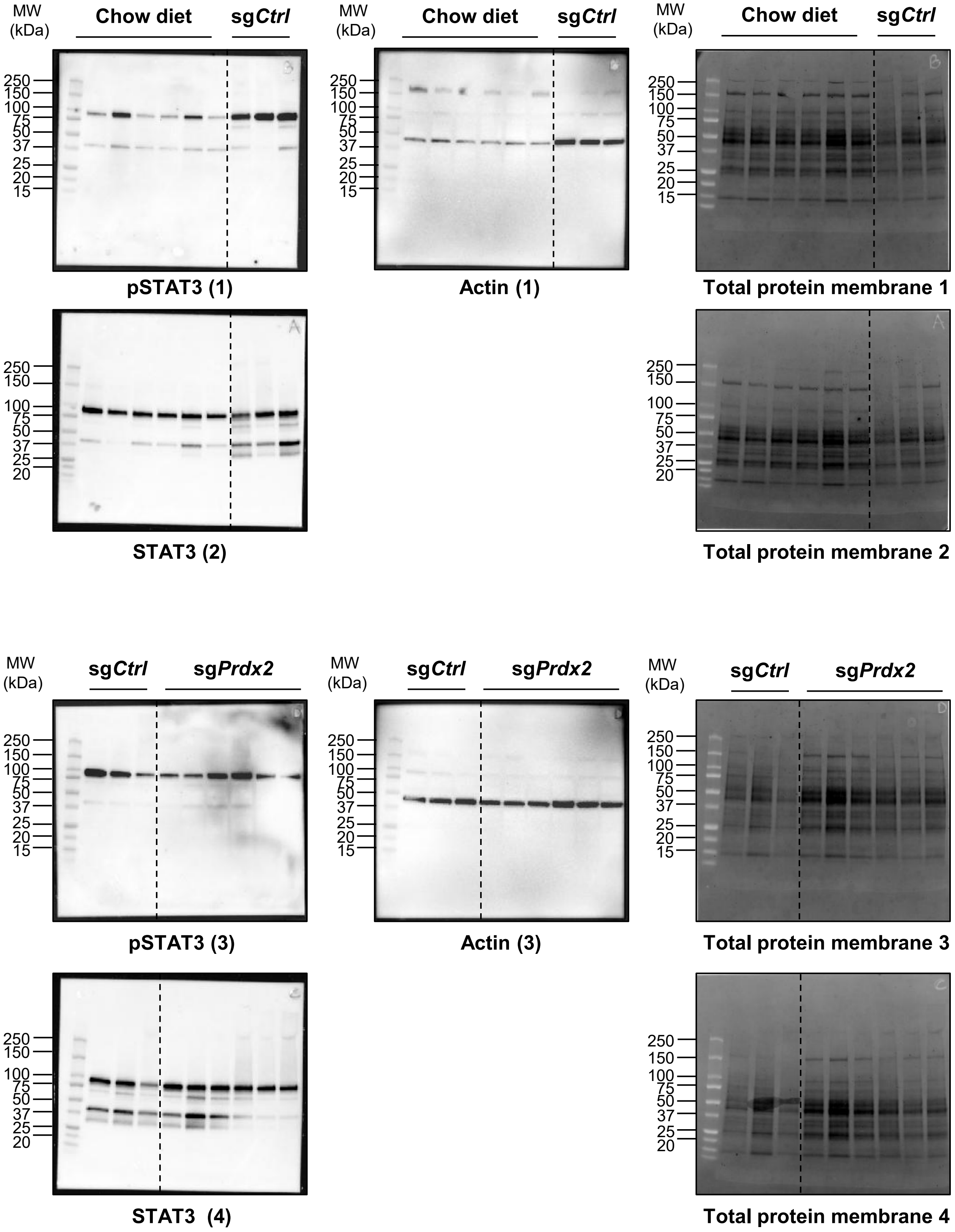
MW (kDa)
Chow diet
sgCtrl
250
150
100
75
50
37
25
20
15
pSTAT3 (1)
Actin (1)
Total protein membrane 1
STAT3 (2)
Total protein membrane 2
sgPrdx2
pSTAT3 (3)
Actin (3)
Total protein membrane 3
STAT3 (4)
Total protein membrane 4

Figure 3D (3/4)

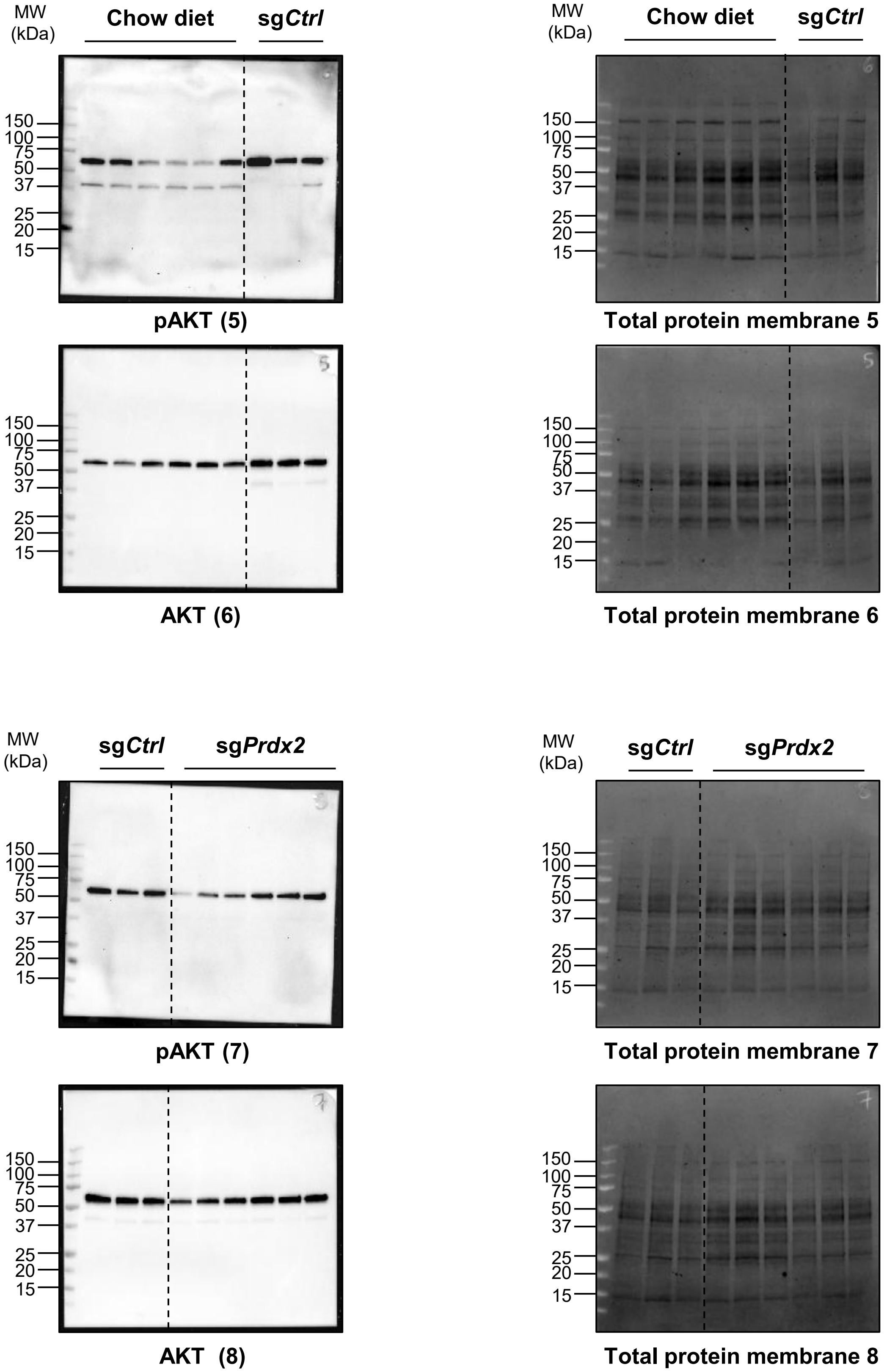
MW (kDa)
Chow diet
sgCtrl
150
100
75
50
37
25
20
15
pAKT (5)
Total protein membrane 5
AKT (6)
Total protein membrane 6
MW (kDa)
sgCtrl
sgPrdx2
pAKT (7)
Total protein membrane 7
AKT (8)
Total protein membrane 8

Figure 3D (4/4)

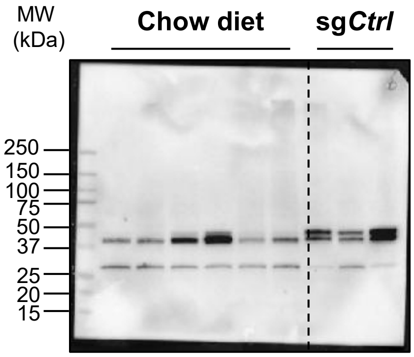

pERK (9)

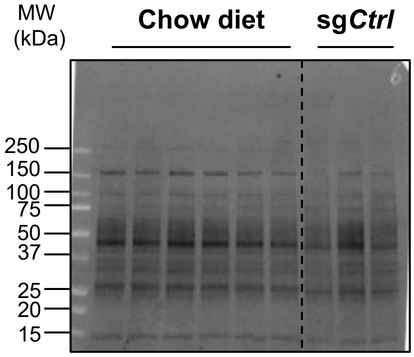

Total protein membrane 9

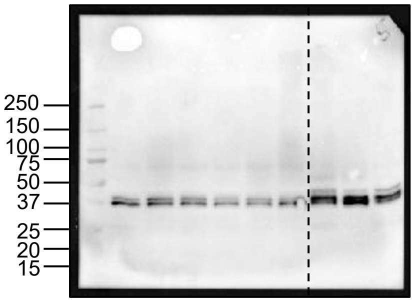

ERK (10)

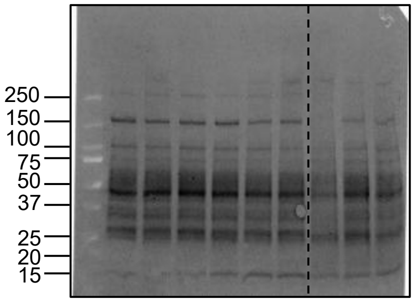

Total protein membrane 10

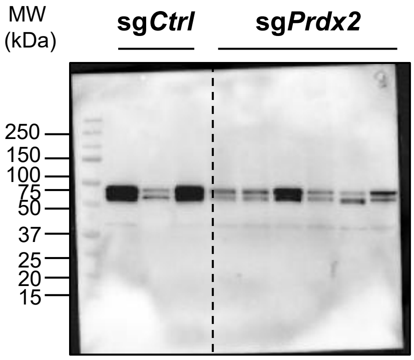

pERK (11)

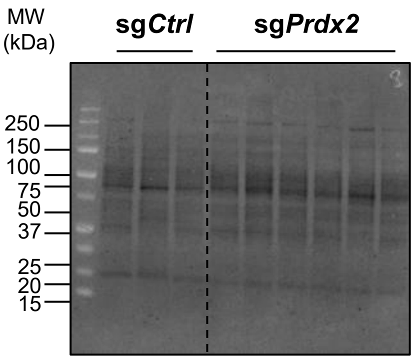

Total protein membrane 11

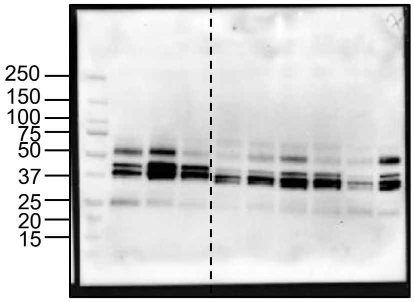

ERK (12)

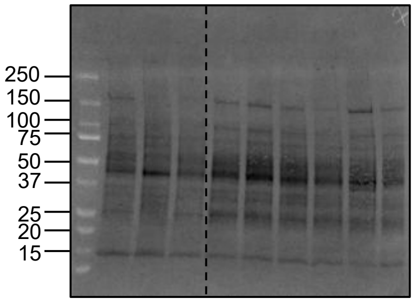

Total protein membrane 12

Figure 4A

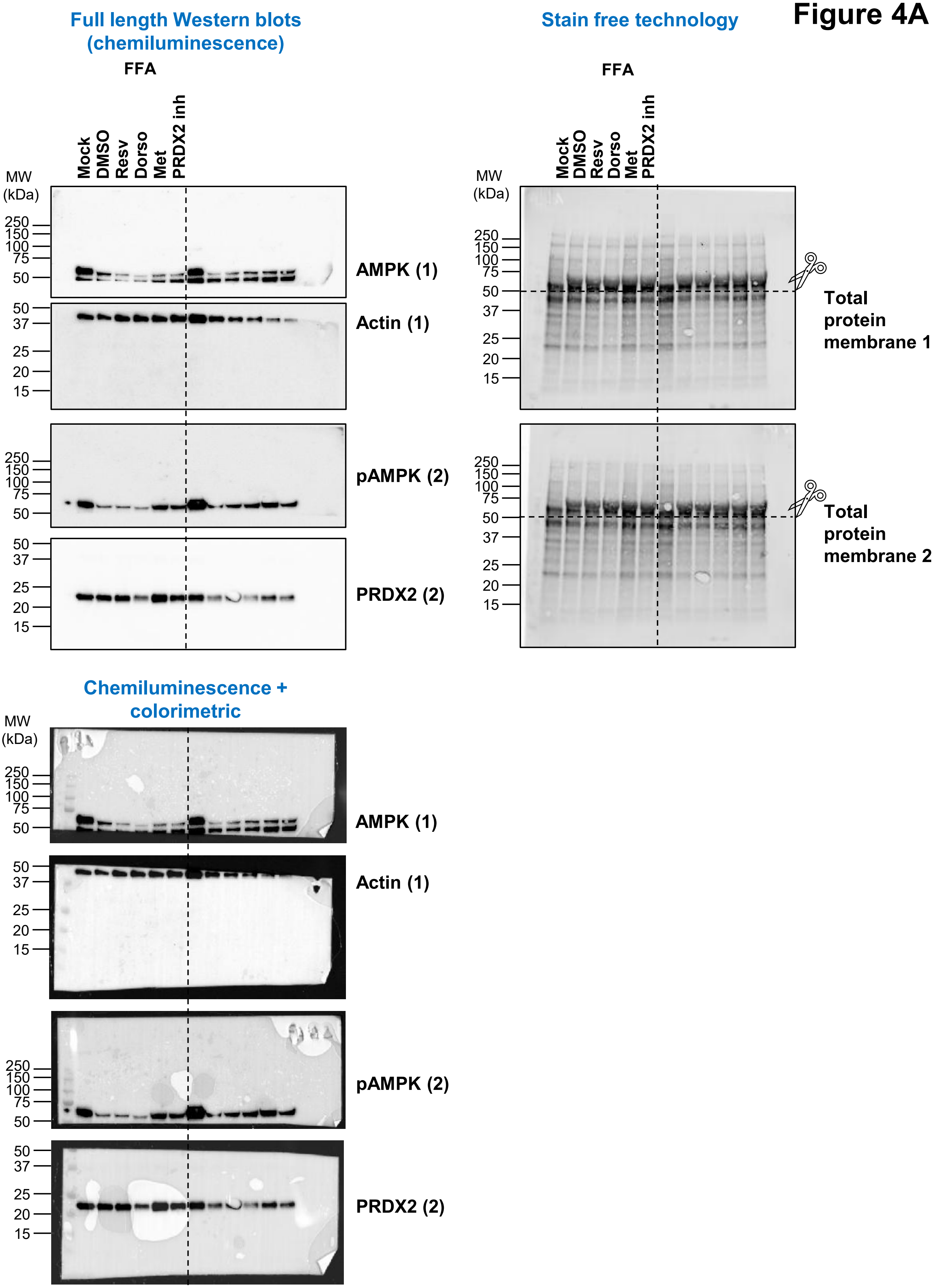
Full length Western blots (chemiluminescence)
FFA
Mock
DMSO
Resv
Dorso
Met
PRDX2 inh
MW (kDa)
250
150
100
75
50
37
25
20
15
AMPK (1)
Actin (1)
pAMPK (2)
PRDX2 (2)
Stain free technology
Total protein membrane 1
Total protein membrane 2
Chemiluminescence + colorimetric

Figure 4C
And Supplementary Figure 13

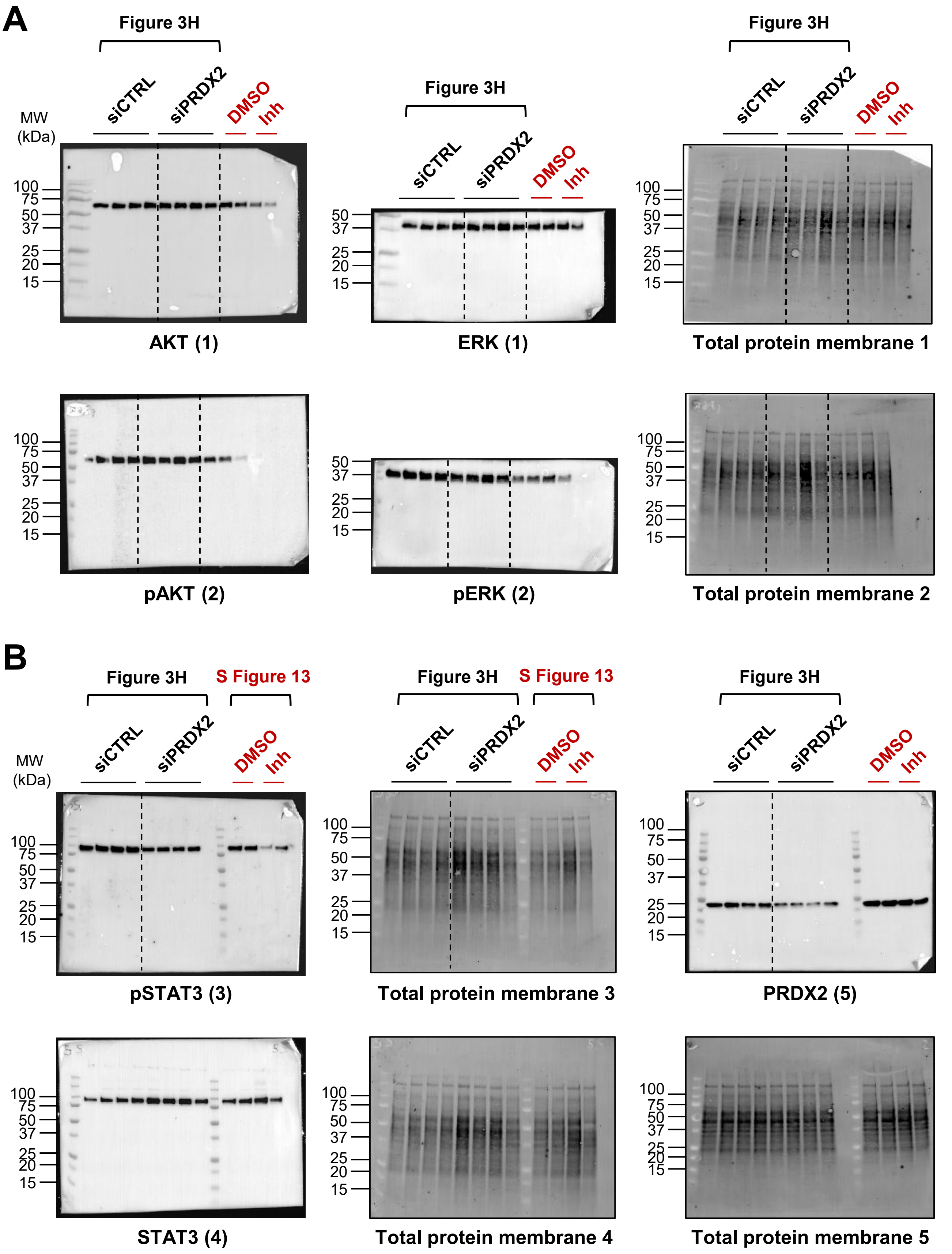

Figure 6B

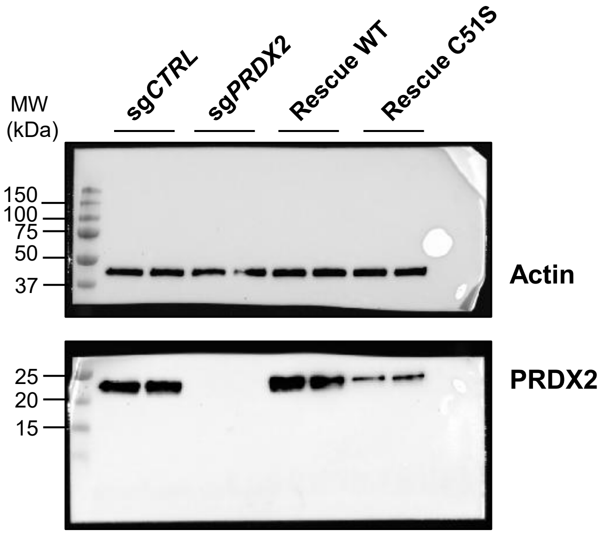

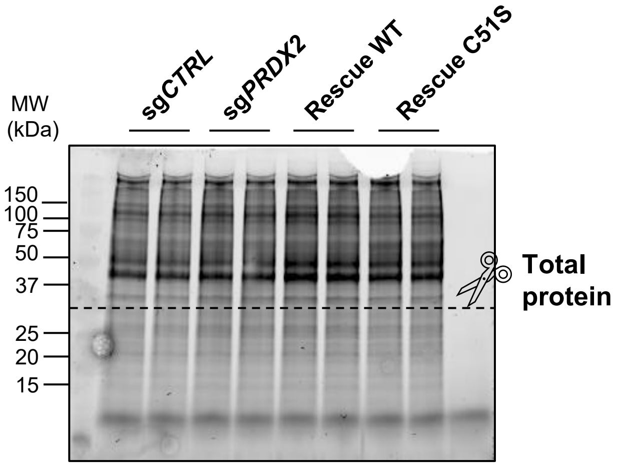

Figure 5E

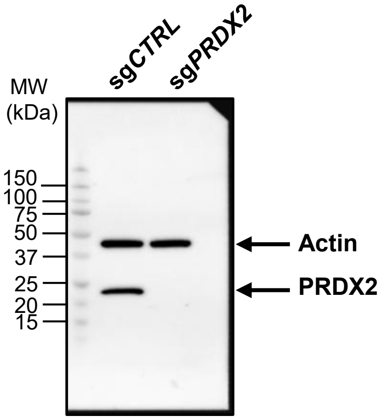

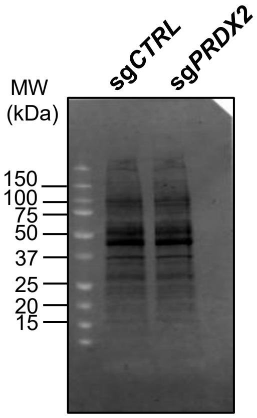

Figure 7A

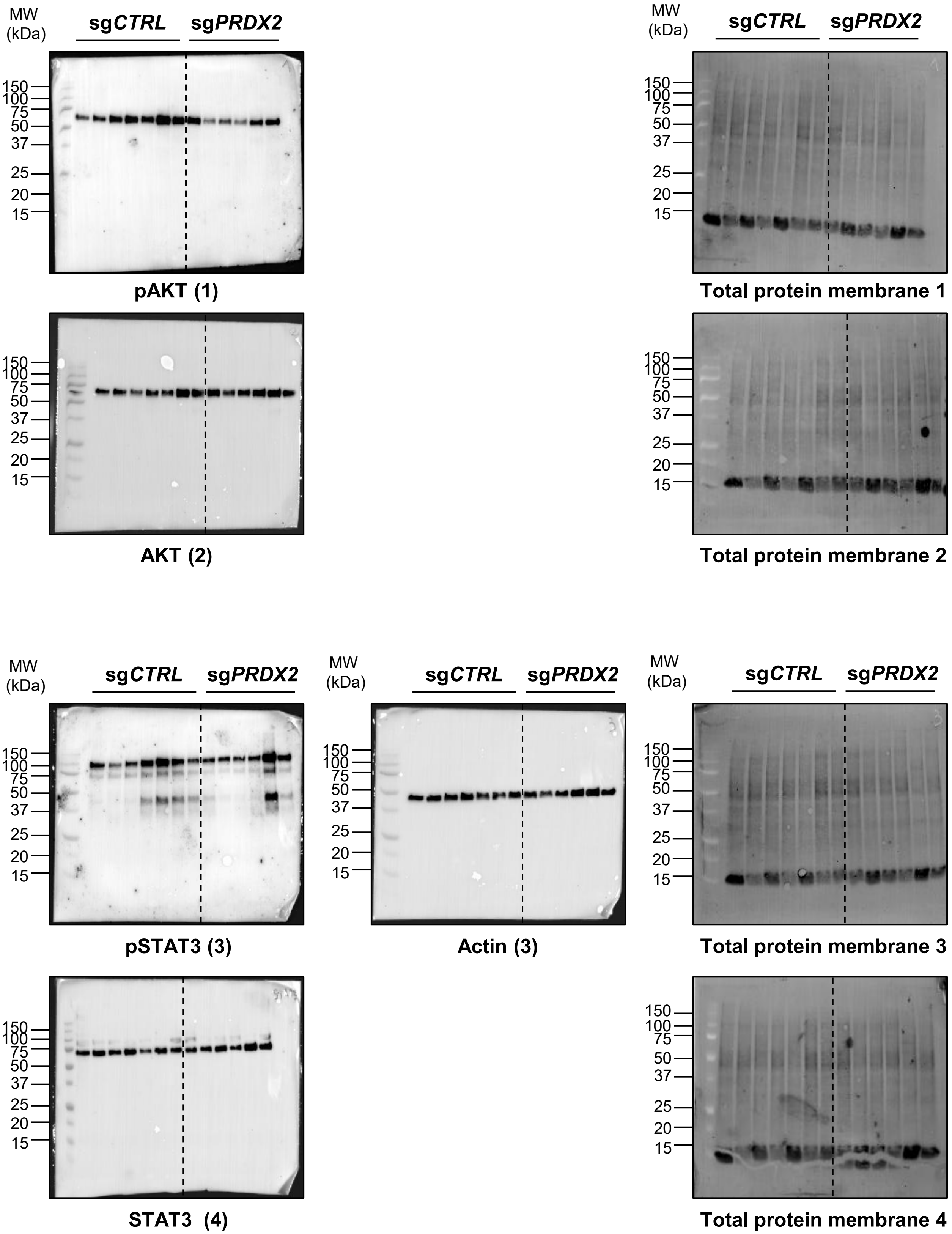
MW (kDa)
sgCTRL
sgPRDX2
150
100
75
50
37
25
20
15
pAKT (1)
AKT (2)
Total protein membrane 1
Total protein membrane 2
pSTAT3 (3)
Actin (3)
Total protein membrane 3
STAT3 (4)
Total protein membrane 4

Figure 7B

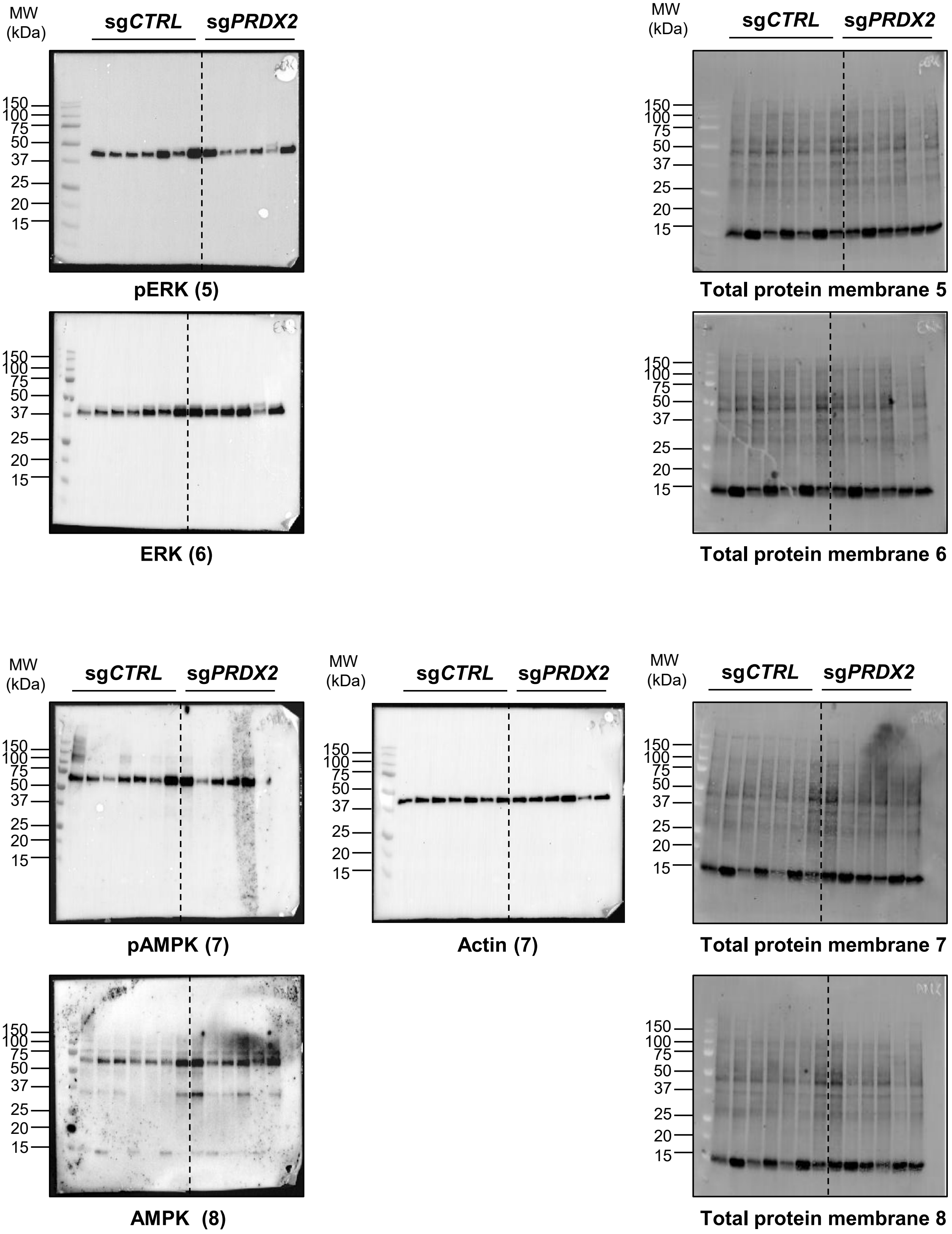
MW (kDa)
sgCTRL
sgPRDX2
150
100
75
50
37
25
20
15
pERK (5)
Total protein membrane 5
ERK (6)
Total protein membrane 6
pAMPK (7)
Actin (7)
Total protein membrane 7
AMPK (8)
Total protein membrane 8

Figure 8D

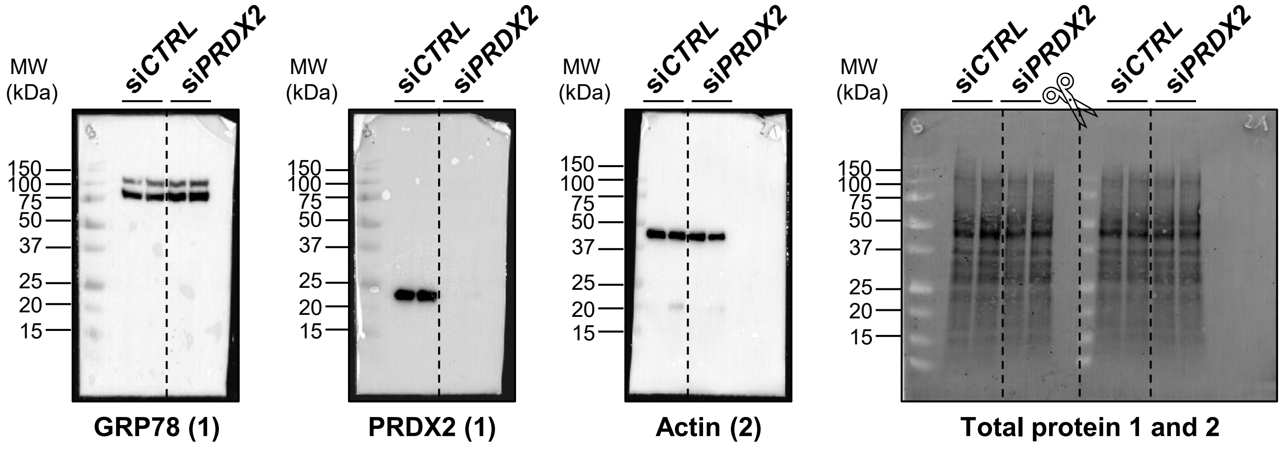

Figure 8E

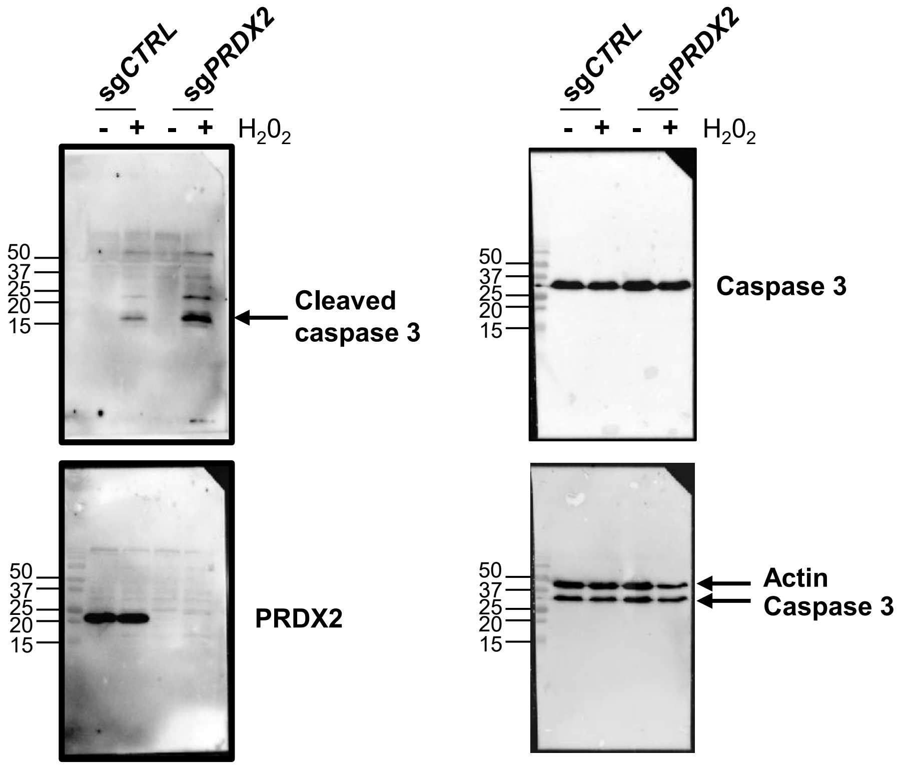

Figure 9A

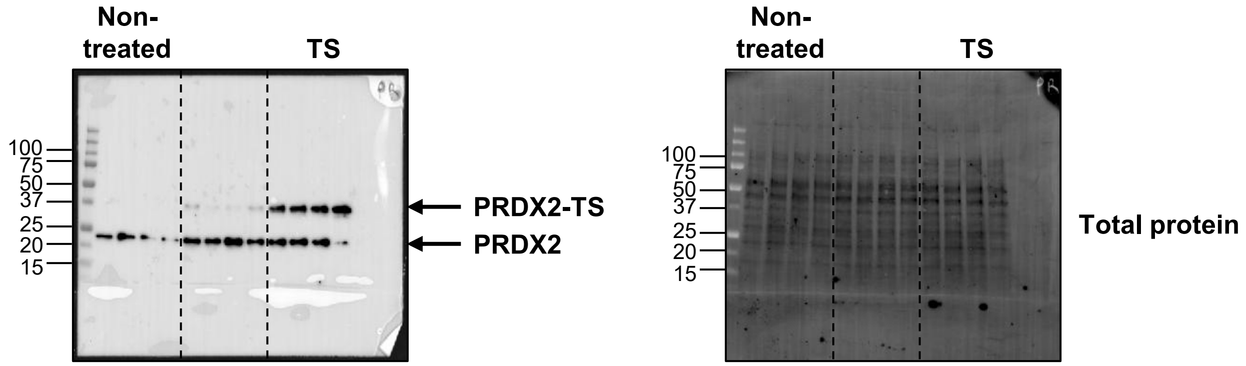

Figure 9B

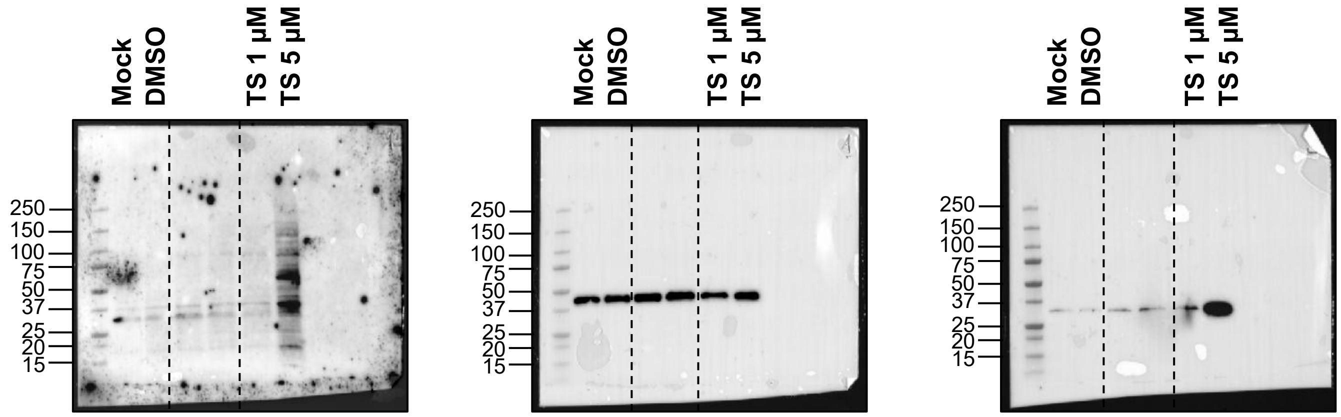

Supplementary Figure 3

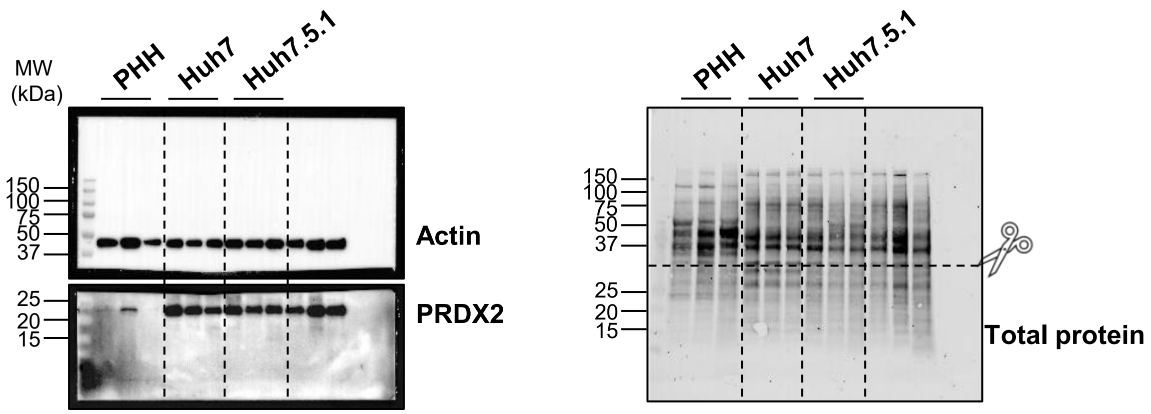

Supplementary Figure 5

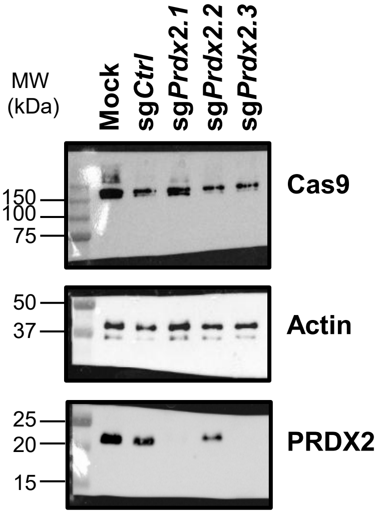

Supplementary Figure 6

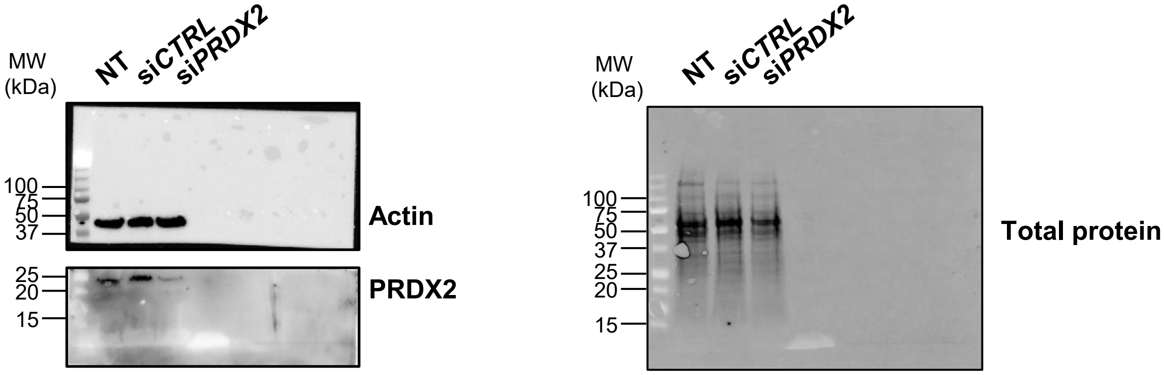

Supplementary Figure 8

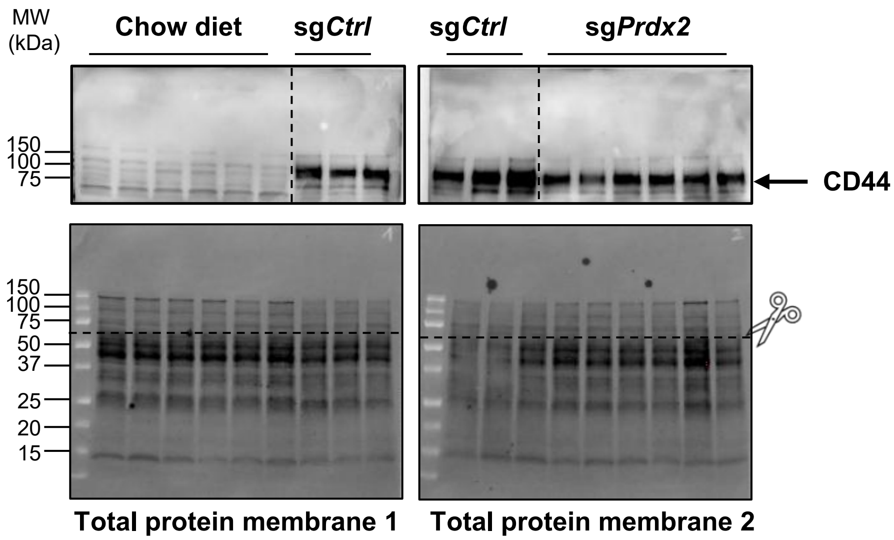

Supplementary Figure 9

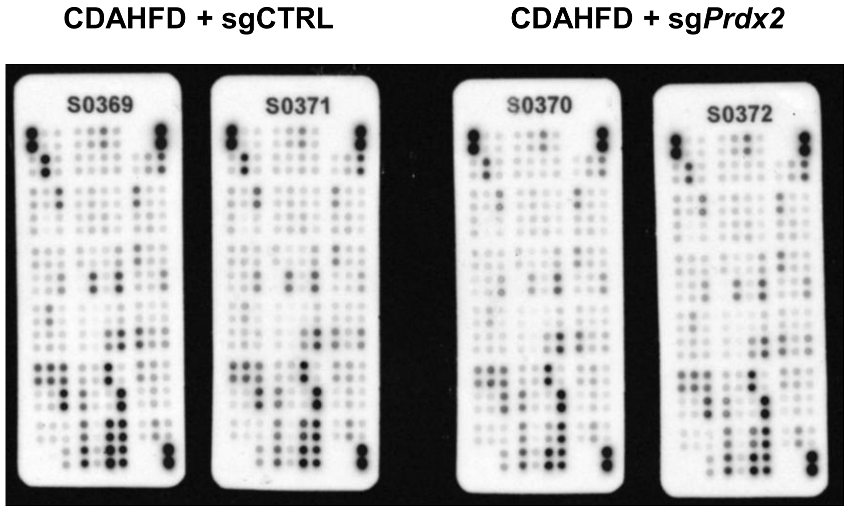

Supplementary Figure 10

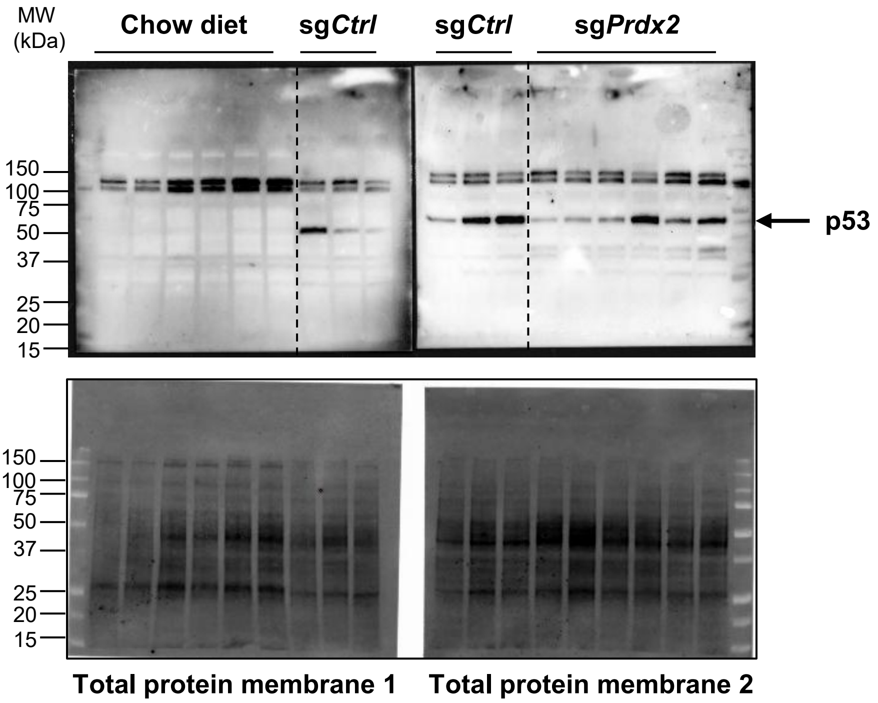

Supplementary Figure 13 second experiment

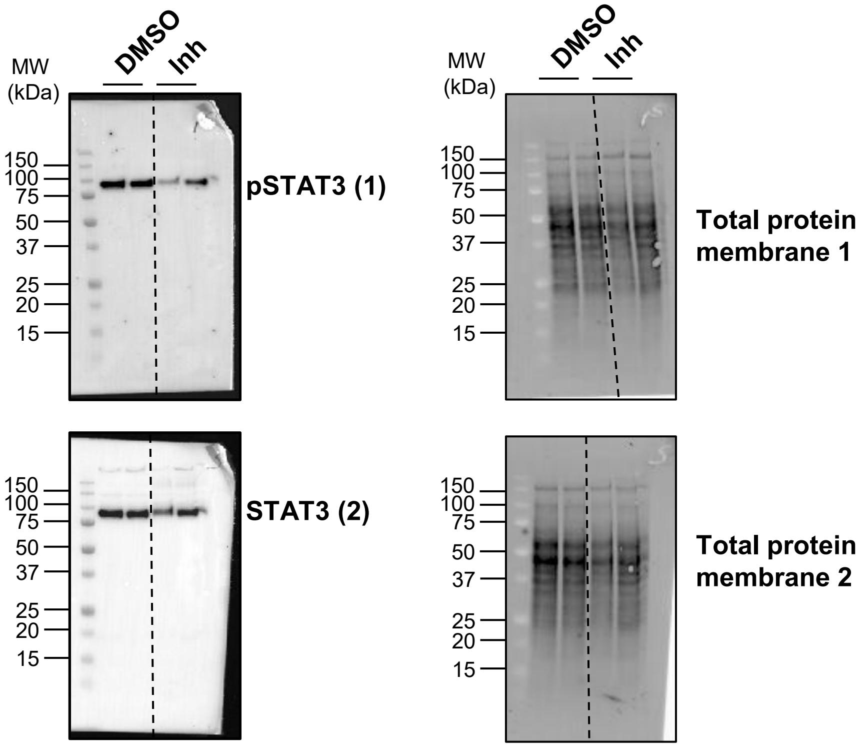

Supplementary Figure 15

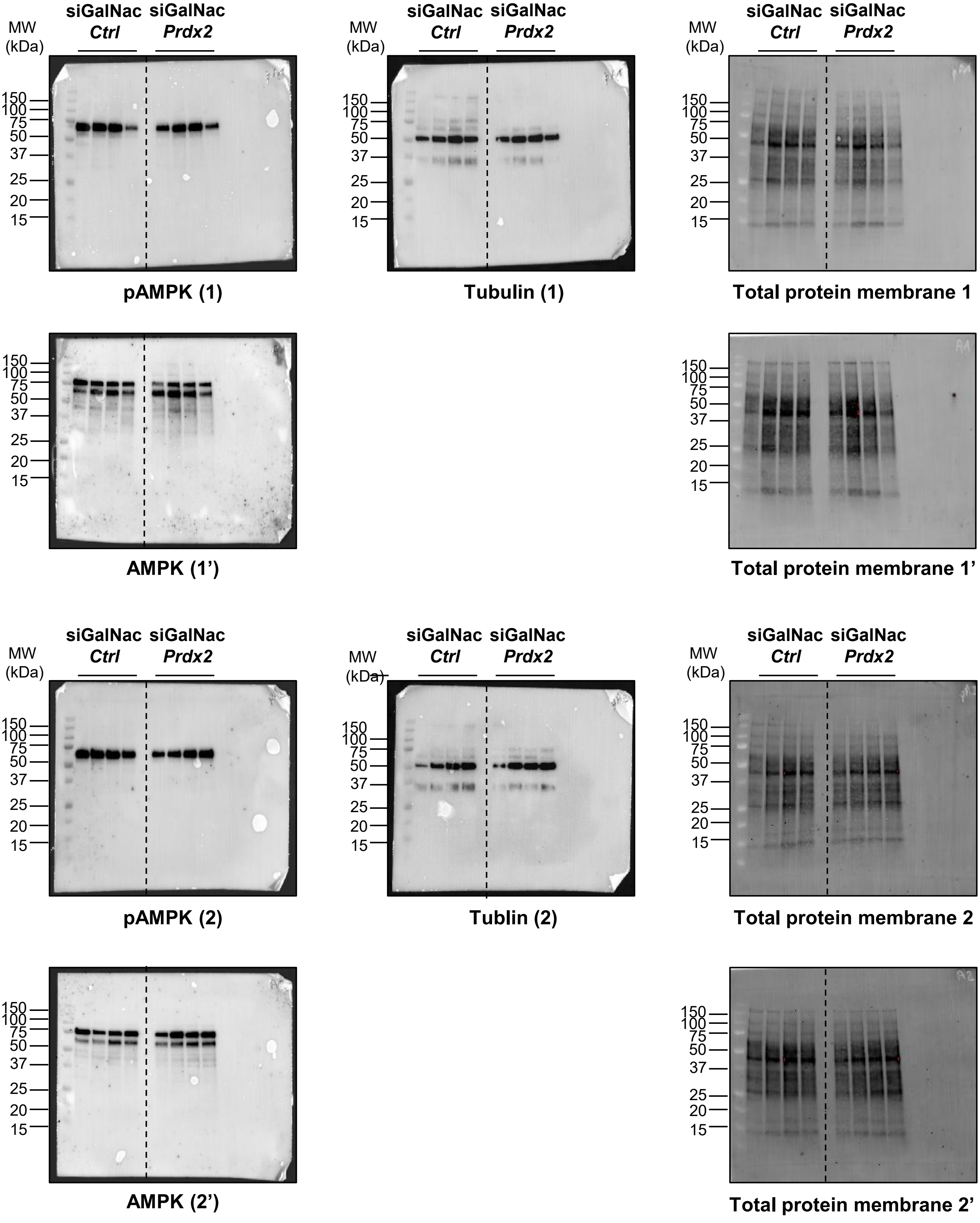

Supplementary Figure 16

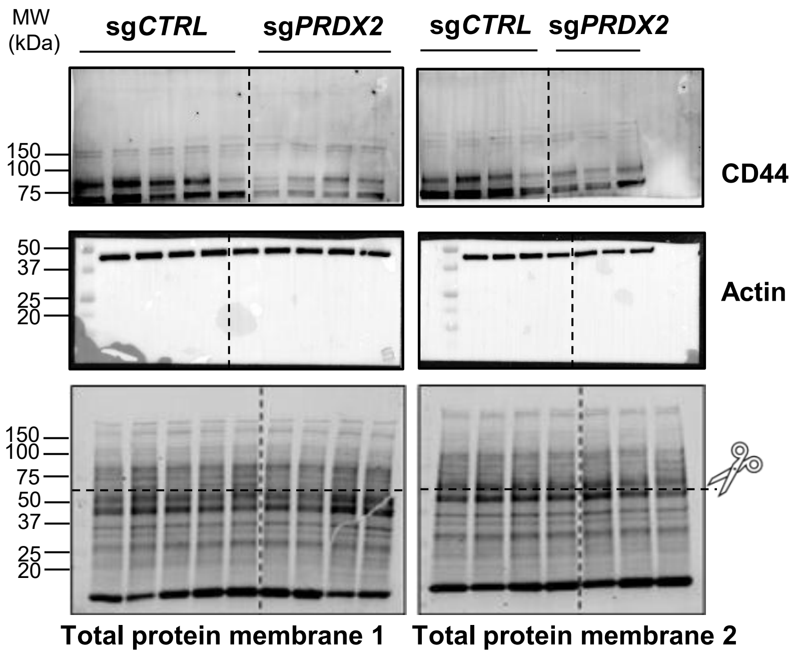

Supplement: Unedited blot and gel images [file jci-135-169395-s247.pdf]
